# Lanthanide–tetrazine probes for bio-imaging and click chemistry†

**DOI:** 10.1039/d4sc02335h

**Published:** 2025-01-22

**Authors:** Benjamin Woolley, Yue Wu, Li Xiong, Ho-Fai Chau, Junhui Zhang, Ga-Lai Law, Ka-Leung Wong, Nicholas J. Long

**Affiliations:** a Department of Chemistry, Imperial College London Molecular Sciences Research Hub, 82 Wood Lane, White City Campus London W12 0BZ UK n.long@imperial.ac.uk; b Department of Chemistry, Hong Kong Baptist University Kowloon Tong Hong Kong SAR China; c Department of Applied Biology and Chemical Technology, The Hong Kong Polytechnic University Hung Hom Hong Kong SAR China ga-lai.law@polyu.edu.hk klwong@hkbu.edu.hk

## Abstract

The blood–brain-barrier prevents many imaging agents and therapeutics from being delivered to the brain that could fight central nervous system diseases such as Alzheimer's disease and strokes. However, techniques such as the use of stapled peptides or peptide shuttles may allow payloads through, with bioconjugation achieved *via* bio-orthogonal tetrazine/norbornene click chemistry. A series of lanthanide–tetrazine probes have been synthesised herein which could be utilised in bio-orthogonal click chemistry with peptide-based delivery systems to deliver MRI agents through the blood–brain-barrier. The Gd complexes show higher relaxivities than the clinical standard of Gd(DOTA) at 1.4 T and phosphorescence is observed from the Eu and Tb complexes *via* tetrazine sensitization, with supporting *in vitro* cytotoxicity and cell imaging. A bio-orthogonal click reaction between a Gd-tetrazine complex and a cyclic-RGD-norbornene conjugate was successful and the resulting clicked probe demonstrated enhanced relaxivity and could potentially act as a peptide shuttle for the Gd MRI agent.

## Abstract 2

Lanthanide–tetrazine complexes were synthesised and evaluated *via* optical spectroscopy and relaxivity. A bio-orthogonal click reaction with a cyclic-RGD-peptide was successful and the resulting Gd complex demonstrated enhanced relaxivity.

## Graphical abstract

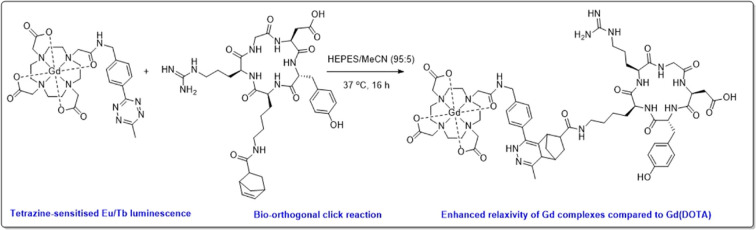

## Introduction

Central nervous system (CNS) diseases such as brain cancers, Alzheimer's disease, and strokes have increased dramatically in the 21st century, corresponding to 13% of the global health burden, exceeding other cancers and cardiovascular diseases.^1^ In general, attempting to deliver imaging agents or therapeutics to the brain is challenging and faces a low success rate due to the blood–brain barrier (BBB).^2,3^ The BBB is a semipermeable membrane made from endothelial, astrocyte and pericyte cells, forming tight-junctions that restrict cell-to-cell transport from blood to neural tissues to protect the brain from neurotoxins (and *vice versa*).^4–6^ This means that most macromolecules, molecular imaging probes, and therapeutics are unable to pass through the BBB.^7^ Given this difficulty in penetrating the BBB safely, several methods have been developed for passing through the BBB. Molecules can pass through the BBB naturally *via* passive diffusion; however, this is limited by molecular weight (<500 Da) as well as factors such as the lipophilicity and hydrogen-bonding ability.^8,9^ Alternative approaches involve the cyclisation of a peptide structure to enhance permeability (such as stapled peptides) or the use of ‘trojan horse’ peptides which can be recognised by specific receptors on the BBB surface and carry through molecules which would not penetrate the BBB by themselves (peptide shuttles). Cyclic peptides can show increased cell penetration and binding affinities due to their enforced bioactive α-helical structure, reduced conformational freedom, and higher resistance to proteolysis.^10,11^ The cyclic-RGD peptide has been used as a targeting group for the integrin α_v_β_3_ receptor which is up-regulated on endothelial cells when angiogenesis occurs and overexpressed on the surface of cancer cells.^12,13^ Hence, it is an attractive molecular target for the early detection and treatment of cancers. The cyclic RGD peptide has also been shown to be able to cross the BBB and deliver drug payloads to malignant glioma tumours *in vivo*, as well as many radiolabelled-cyclic RGD peptides being tested *in vivo* and in clinical trials.^11,14–16^

Click chemistry has proven to be a versatile and efficient way of linking two molecular entities together under facile conditions. The tetrazine/norbornene click reaction has gained much interest over recent years due to its exceptional kinetics, ability to proceed without metal catalysts, and its bio-orthogonality.^17^ The bio-orthogonal reaction between an electron-poor 1,2,4,5-tetrazine and strained alkene proceeds *via* an inverse-electron demand Diels–Alder (IEDDA) reaction, featuring a [4 + 2] cycloaddition before retro-[4 + 2] cycloelimination and release of dinitrogen to generate a 1,4-dihydropyridazine.^18^ This type of click reaction has found application in local prodrug activation,^19^ ‘turn-on’ optical imaging probes utilising FRET,^20,21^ or in radiochemistry with a number of PET and SPECT probes being developed for a pre-targeting approach or radiolabelling of peptides with a number of macrocyclic-tetrazine ligands featuring different linkers being reported in the literature.^22–27^ However, this type of click chemistry has seldom been applied to lanthanide imaging probes *e.g.* Gd-containing MRI probes.

Lanthanide complexes can be used for a wide range of imaging applications. For example, Gd complexes can be used as MRI contrast agents due to their strongly paramagnetic nature.^28^ Eu and Tb complexes can be used as optical imaging agents for time-resolved microscopy due to their long-lived phosphorescent nature, large Stokes' shifts, and sharp emission profiles which can be separated from biological autofluorescence.^29,30^ Despite these promising imaging properties of lanthanide probes, they have seldom found application for CNS diseases, where they would have to cross the BBB, due to their large molecular weight.

Herein, the synthesis of a series of Gd, Eu, and Tb lanthanide–tetrazine complexes is described, which can be used in bio-orthogonal click chemistry with peptide BBB delivery vectors. The relaxivity of the Gd complexes is evaluated and compared to the clinical standard of Gd(DOTA). The analogous Eu and Tb complexes are studied *via* UV-vis, fluorescence, and phosphorescence spectroscopy. Bio-orthogonal tetrazine–norbornene click reactions using a cyclic-RGD peptide are then reported and the relaxivity of the clicked probes evaluated.

## Results and discussion

### Synthesis of lanthanide complexes

**Scheme 1:**
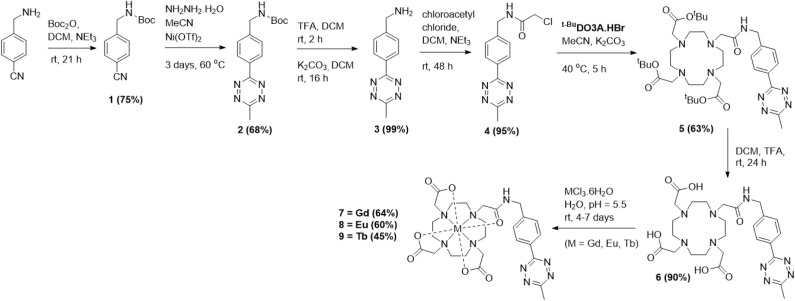
Synthesis of target complexes 7–9.

**Scheme 2:**
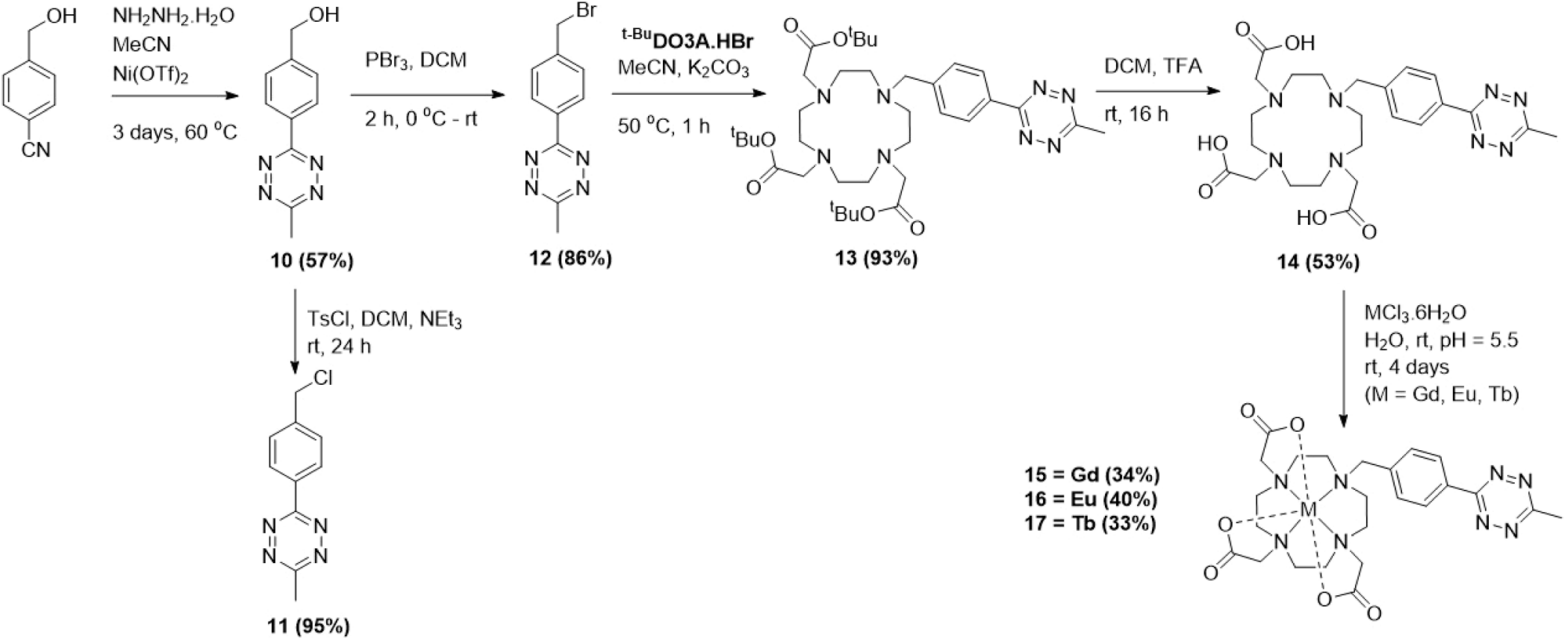
Synthesis of target complexes 15–17.

Two sets of lanthanide complexes were synthesised, containing either an acetyl linker or direct attachment between the tetrazine and macrocycle. To synthesise complexes 7–9, 4-(aminomethyl)benzonitrile hydrochloride was first Boc-protected following the procedure by Hernández-Gil *et al.* to give 1 (Scheme 1).^31^ The formation of the tetrazine scaffold was accomplished following the one-pot, Ni-catalysed procedure reported by Yang *et al.* to give 2 as a bright pink solid.^32^ The Boc-group in 2 was then deprotected using trifluoroacetic acid (TFA) to give the TFA salt of 3, before the free-amine tetrazine 3 was produced after stirring with saturated potassium carbonate solution. The amino-tetrazine 3 was reacted with chloroacetyl chloride to give 4 as a dark pink solid in quantitative yield. Next, 4 was attempted to be attached to the *^t^*^-Bu^DO3A·HBr macrocycle, which had been synthesised following the procedure by Jagadish *et al.*^33^ Initially, this was attempted at reflux in acetonitrile with potassium carbonate or caesium carbonate. However, this caused decomposition of the tetrazine, with the loss of the aromatic protons at 8.5 ppm in the ^1^H-NMR spectrum. This decomposition was most likely thermally induced, with several related examples in the literature involving the entropically-favoured release of dinitrogen gas and generation of organic nitrile species.^34,35^ A successful reaction occurred when using potassium carbonate at 50 °C for 4 hours, where 5 was obtained as a dark pink solid. To produce the free-ligand 6, 5 was deprotected using TFA before the lanthanide complexations for either Gd, Eu, or Tb were performed following a general procedure (Scheme 1). The free ligand 6 and a slight excess of the lanthanide chloride hexahydrate salt (1.1 equivalents) were dissolved in water and the pH adjusted to 5.5 using 0.1 M sodium hydroxide and allowed to stir at room temperature. After 24 hours, an additional equivalent of the lanthanide salt was added, and the pH readjusted to 5.5. After 3 further days, the solvent was removed *in vacuo* before purification was achieved *via* reverse-phase column chromatography. After removing the solvents *via* lyophilisation, the lanthanide–tetrazine complexes 7–9 were isolated as pink solids in good yields. The expected molecular ions were found *via* ES^+^ mass spectrometry (MS) for complexes 7–9, with the expected isotope patterns being observed. Purity was confirmed by LCMS, with a single peak in the UV-trace for complexes 7–9, showing that no free-ligand 6 remained. A xylenol orange test was also performed on each sample to confirm that no free-lanthanide ions were present. The ^1^H-NMR spectrum of the Eu complex 8 in D_2_O showed the expected large range of broad signals due to the paramagnetic nature of the complex, with 4 main peaks being observed in the 30–35 ppm region, indicating that the square-antiprism (SAP) isomer is dominant in solution, with no peaks being observed in the twisted-square-antiprism (TSAP) region of 10–14 ppm. The same effect is observed in the ^1^H-NMR spectrum of the Tb complex 9, with no peaks observed in the TSAP region. A similar synthetic strategy was employed to synthesise the complexes 15–17 (Scheme 2). Firstly, the hydroxy-tetrazine 10 was synthesised following the procedure by Yang *et al.*^32^ Initially, 10 was attempted to be tosylated using toluenesulfonyl chloride following the procedure by Da Pieve *et al.*^36^ However, the chloro-tetrazine 11 was always obtained instead (details in the ESI†).^37^ Tetrazine 10 was instead reacted with phosphorous tribromide to give the bromo-tetrazine 12. Reactions of 11 or 12 with *^t^*^-Bu^DO3A·HBr using potassium carbonate at room temperature overnight were unsuccessful, with tetrazine decomposition observed, potentially decomposing *via* photolysis.^34^ Successful reactions were achieved by heating 12 at 50 °C for one hour to give 13. The free ligand 14 was then obtained following TFA deprotection and the lanthanide complexation reactions for 15–17 with Gd, Eu, and Tb were then performed following the same standard procedure as for 7–9 (Scheme 2). After 4 days, the solvent was removed *in vacuo* and product formation in the crude samples was confirmed for 15–17 with the expected molecular ions and isotope patterns being observed by ES^+^ MS. However, after purifying *via* reverse-phase column chromatography, only the free ligand 14 was obtained for each sample, with the lanthanide ions dissociating from the chelate on the column due to the acidic pH. The complexes 15–17 were then successfully isolated *via* reverse-phase chromatography under neutral conditions. Purity was established *via* LCMS with one single peak being observed in the UV-trace for each complex, showing that no free-ligand 14 remained. A xylenol orange test was also performed on these complexes, showing that no free-lanthanide ions remained.

### Absorbance and emission spectroscopy

**Fig. 1:**
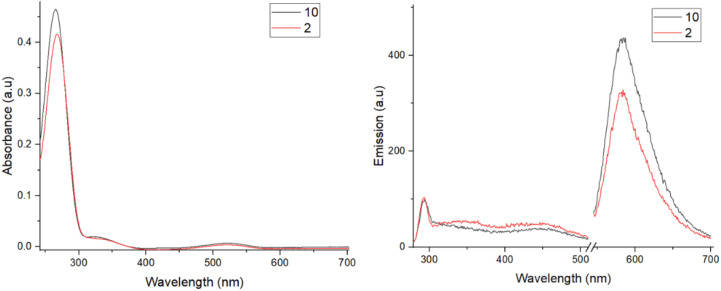
Absorbance (left) and emission (right) spectra for tetrazines 2 and 10 (20 μM, slits 10 nm, PBS buffer, *x*-axis cut to remove 2*λ*_ex_ absorbance).

**Fig. 2:**
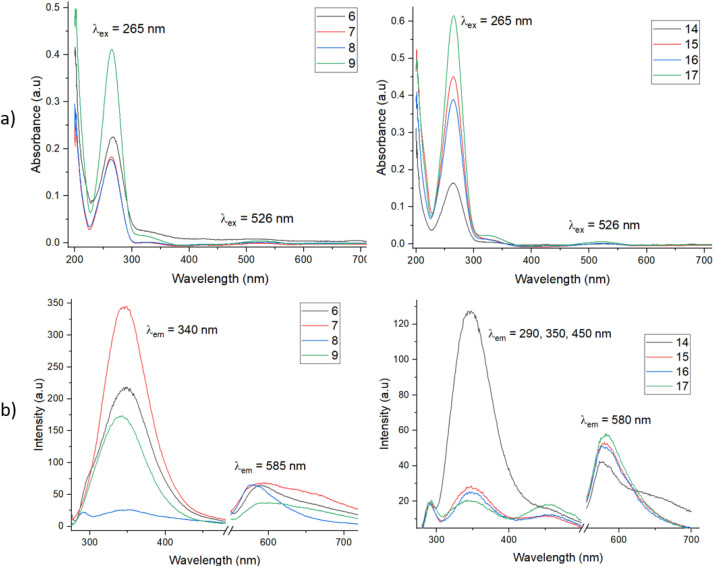
(a) Absorbance spectra for compounds 6–9 (left) and 14–17 (right). (b) Fluorescence emission spectra for compounds 6–9 (left) and 14–17 (right) (20 μM, slits 10 nm, PBS, *x*-axis cut to remove 2*λ*_ex_ absorbance).

To study the photophysical properties of the lanthanide–tetrazine complexes, exemplar tetrazines 2 and 10 were investigated by absorbance and emission spectroscopy (Fig. 1). The tetrazines were dissolved in DMSO/H_2_O mixtures (1 : 1 for 2 and 1 : 9 for 10) before being diluted in PBS (pH 7.4) to make 20 μM solutions. Both tetrazines show similar absorbance profiles, with the major excitation present at around 265 nm, corresponding to a π–π* transition (S_0_–S_*n*_ transition, where *n* is an excited state greater than 1, which differs for different tetrazines depending on their substituents).^38^ It is known in the literature that the substituents on the tetrazine can dramatically affect their absorbance and emission profiles, with the S_0_–S_*n*_ absorbance being particularly affected.^38,39^ An additional, much weaker π–π* transition is also seen at around 330 nm as a shoulder. Finally, a weak absorption is observed at around 528 nm, corresponding to a n–π* transition (S_0_–S_1_), which is responsible for the pink colour of the tetrazines. This transition is formally forbidden, hence the weak oscillator strength.^38^ This type of n–π* transition is observed in only three other organic molecules: 1,2,4-triazines, 1,2-diketones, and azobenzenes.^39^ The extinction coefficients for the 265 nm absorbance for both tetrazines are similar at around 20 000 M^−1^ cm^−1^ and are roughly 80–100 times greater than the extinction coefficient for the 528 nm absorption (around 250 M^−1^ cm^−1^), matching related examples in the literature.^38^ Solutions (20 μM) of free-ligands 6 and 14, and complexes 7–9 and 15–17 were then prepared in PBS. All ligands and complexes showed similar absorption profiles, matching the simpler tetrazine profiles (Fig. 2). The extinction coefficients for the main absorption range from around 8000 to 30 000 M^−1^ cm^−1^, with complexes 15–17 demonstrating higher values than complexes 7–9. The fluorescence emission spectra for 2 and 10 were then recorded (Fig. 1). Following excitation at 265 nm, four main emission peaks are seen with a sharp, weak emission at 290 nm, broad weak emission at around 350 and 450 nm, and a much stronger emission at around 580 nm. The emission at 580 nm is due to the S_1_–S_0_ transition, while the emission peaks at lower wavelengths are due to the S_*n*_–S_0_ transitions. The emission from the S_1_ state at 580 nm is clearly stronger in intensity, showing effective internal conversion from higher excited states.

The fluorescence emission spectra were then recorded for the free-ligands and lanthanide complexes (Fig. 2). For species 6, 7 and 9, the major emission peak is now at 350 nm, demonstrating effective fluorescence emission from the higher excited states (S_*n*_–S_0_) and reduced internal conversion to the S_1_ state, with a much weaker intensity peak observed at 580 nm. Since the oscillator strength for the S_0_–S_*n*_ transition is approximately 100 times stronger than that for the S_0_–S_1_ transition, the radiative decay rate from S_*n*_–S_0_ is now comparable to the internal conversion rate from S_*n*_–S_1_.^38^ For Eu complex 8, the trend is reversed, with a weaker emission at 350 nm than 580 nm, demonstrating significant internal conversion. While the emission intensities of the 350 nm emission (S_*n*_–S_0_) differ significantly between each species, the intensities of the S_1_–S_0_ transition at 580 nm are consistent. For ligand 14, the S_*n*_–S_0_ emission at 350 nm is much higher in intensity than the S_1_–S_0_ 580 nm emission. However, for the complexes 15–17, a different profile is observed. Weak emission is seen at 290, 350, and 450 nm, with a more intense emission observed for the S_1_–S_0_ transition at 580 nm. This emission profile closely matches tetrazine 10. It is likely that the weak emissions at 290 and 450 nm are also present for other species, however they are not clearly observed when the emission at 350 nm is much stronger in intensity. Once again, while the emission intensities of the S_*n*_–S_0_ transition vary between species, the intensities of the S_1_–S_0_ emission at 580 nm are similar. Clearly the emission intensities and kinetics of internal conversion and fluorescence are strongly influenced by not only the tetrazine substituents but also the particular lanthanide ion.

### Phosphorescence spectra

**Fig. 3:**
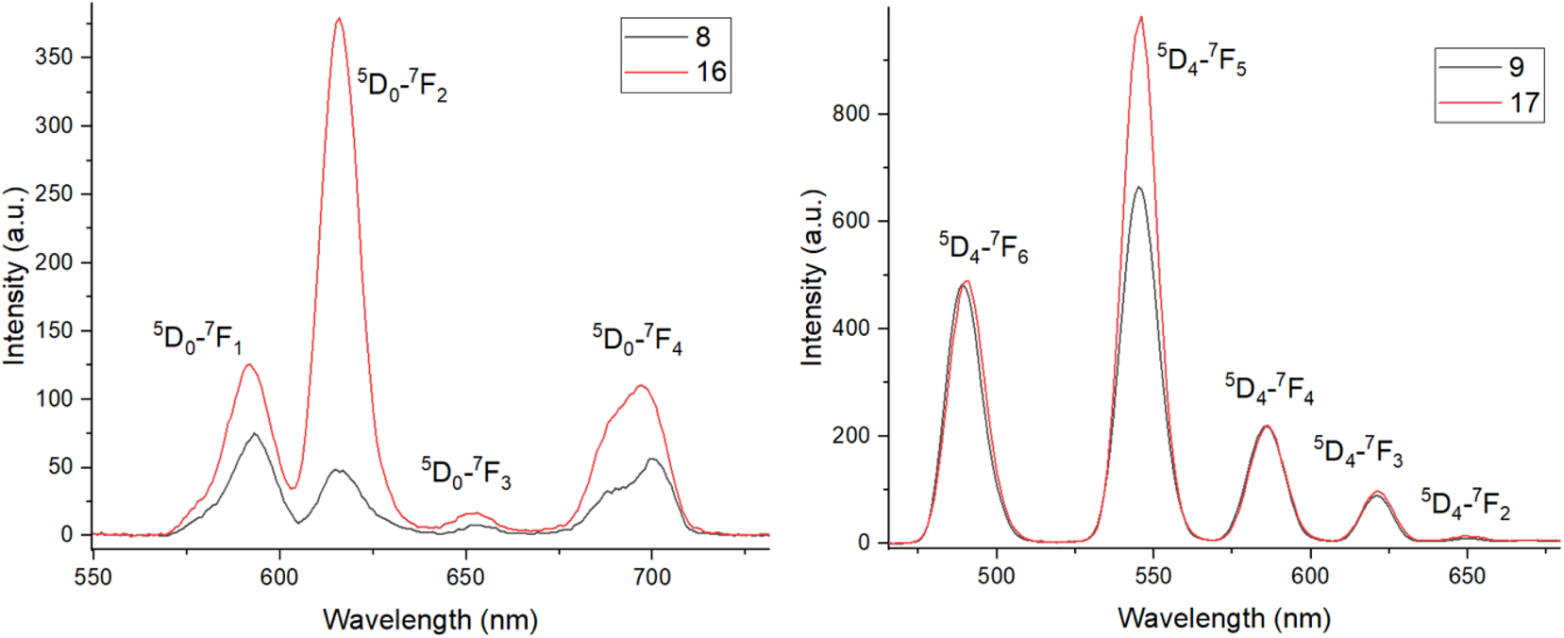
Phosphorescence spectra for Eu complexes 8 and 16 (left) and Tb complexes 9 and 17 (right) (20 μM, slits 10 nm, PBS, 0.1 ms delay).

The phosphorescence spectra for the Eu complexes 8 and 16 and Tb complexes 9 and 17 were then recorded, following a 0.1 ms delay for 20 μM solutions in PBS (Fig. 3). This removed the short-lived organic tetrazine fluorescence signals and standard sharp phosphorescence emission peaks were observed for both the Eu and Tb complexes *via* the antenna effect following excitation of the tetrazine fluorophore antenna at 265 nm. The emission was significantly stronger for the Tb complexes compared to the Eu complexes, most likely due to the better energy match between the tetrazine triplet excited state and the typical Tb excited state energy. While the tetrazine triplet state energy was not determined, the singlet excited state lies at around 28 500 cm^−1^, hence the triplet excited state is likely to lie much closer to the typical Tb excited state than Eu. The phosphorescence emission from the Eu complex 8 is much weaker than that of Eu complex 16, most likely due to the reduced distance between the tetrazine antenna and Eu centre. This effect is much less apparent in the Tb complexes 9 and 17, however, with the emission intensities being similar for both complexes. No phosphorescence was observed when the Eu and Tb complexes were excited at 520 nm or for the free-ligands or Gd complexes when excited at 265 nm, as expected. While some related examples in the literature have not found evidence of long-lived triplet states for tetrazines, they are clearly present here due to lanthanide phosphorescence being observed.^38^ To the best of our knowledge, this is the first example of lanthanide sensitization from a tetrazine antenna which is not directly coordinated to the lanthanide ion.

### Relaxivity data of tetrazine complexes

**Fig. 4:**
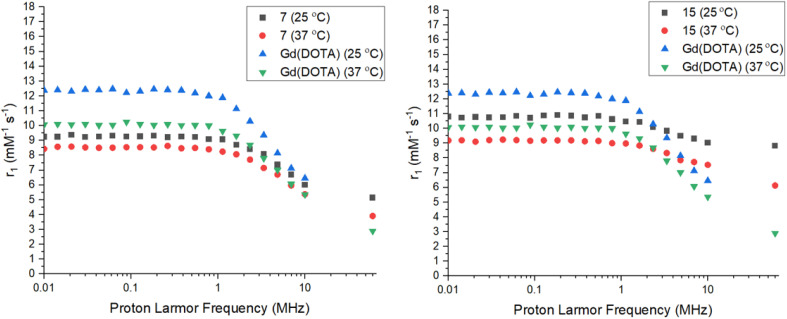
NMRD profiles for Gd complex 7 (left) and 15 (right) *vs.* Gd(DOTA) at 25 and 37 °C.^40,41^

The relaxivity (*r*_1_) of the Gd complexes 7 and 15 were investigated using a 0.25 T Fast Field Cycling NMR relaxometer at frequencies from 0.01 to 10 MHz, with the effective Gd^3+^ ion concentration determined using the Evans' NMR method. The *R*_1_ values (1/*T*_1_) were acquired at 25 °C and 37 °C and converted into *r*_1_ values using the equation in the ESI.† The *r*_1_ measurements at 60 MHz (1.4 T) were performed *via* a *T*_1_-inversion recovery experiment (details in the ESI†). The NMRD profiles are shown in Fig. 4 and the key data summarized in Table 1. Both Gd complexes 7 and 15 display the typical shape for a small molecular-weight complex, with a constant relaxivity below 1 MHz, and a decrease in relaxivity with ^1^H Larmor frequencies above 1 MHz. The relaxivity for 15 is higher than 7 at all frequencies, due to the increased hydration number of the complex. From hydration number calculations (details in the ESI†) complexes 7–9 are *q* = 1, while complexes 15–17 are *q* = 2. A much less intense decay in relaxivity at higher frequencies is observed for 15 compared to 7 too, most likely due to the difference in hydration number for these complexes. The complexes show lower relaxivities at higher temperature as expected due to the faster rotational correlation time due to their increased kinetic energy.^40^ The relaxivities for 7 and 15 are comparable at lower frequencies, but differ more drastically as the frequency increases, with the relaxivity for 15 being almost twice the relaxivity of 7 at 60 MHz. The value of relaxivity for 15 is not exactly twice the value for 7 at 60 MHz due to the effect of the outer-sphere water molecules also affecting the value. The relaxivities were also compared to the clinical standard of Gd(DOTA) which is a *q* = 1 complex of similar molecular weight and size to the tetrazine complexes 7 and 15.^40,41^ At lower frequencies, the relaxivity of Gd(DOTA) is higher than complexes 7 and 15, however at higher frequencies the relaxivity becomes comparable to complex 7. Gd(DOTA) shows a higher relaxivity at lower frequencies due to the higher symmetry of the complex and hence, slower electronic relaxation.^42^ The relaxivity for complex 15 is higher than Gd(DOTA) at higher frequencies due to the increased hydration number. At the clinically-relevant frequency of 60 MHz (1.4 T), the relaxivities for both 7 and 15 are higher than Gd(DOTA) at 37 °C.^41^ The presence of an amide arm has been shown to lower relaxivity, however this effect does not seem to be significant in complex 7.^43^ Both Gd(DOTA) and 7 are *q* = 1 complexes of similar molecular weight and hence, the increase in relaxivity could be due to the decrease in rotational correlation time of the slightly larger molecular weight complex 7. The presence of the tetrazine could also hydrogen bond to water molecules, affecting the exchange kinetics or definition of the second hydration sphere. There also appears to be less temperature dependence on relaxivity for complexes 7 and 15 compared to Gd(DOTA), demonstrating that the rotational motion of the Gd(DOTA) chelate is affected more significantly by temperature.

**Table 1:** Summary of relaxivity data for complexes 7, 15, and 19 at 25 and 37 °C

Complex	*r*_1_ 0.01 MHz (mM^−1^ s^−1^)	*r*_1_ 10 MHz (mM^−1^ s^−1^)	*r*_1_ 60 MHz (mM^−1^ s^−1^)
7 (25 °C)	9.24	6.00	5.14
7 (37 °C)	8.44	5.36	3.91
15 (25 °C)	10.80	9.02	8.82
15 (37 °C)	9.18	7.53	6.13
19 (25 °C)	10.77	7.80	9.87
19 (37 °C)	10.24	7.18	8.29

### Click chemistry

**Scheme 3:**
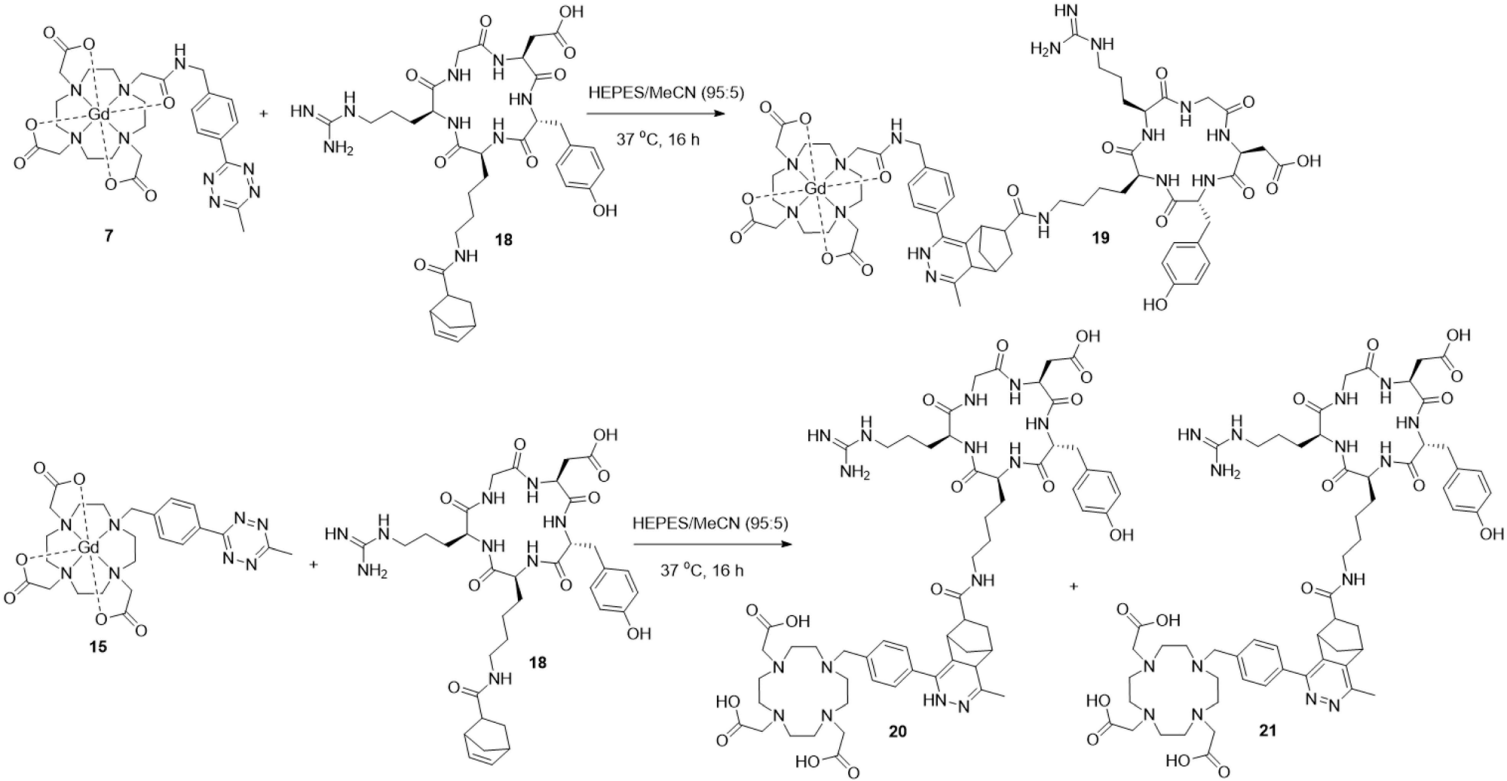
Successful bio-orthogonal click reaction between complex 7 and cyclic-RGD-norbornene 18 to give the clicked product 19 (top) and unsuccessful click reaction between complex 15 and cyclic-RGD-norbornene 18 to give free-ligand clicked products 20 and 21 (bottom).

**Fig. 5:**
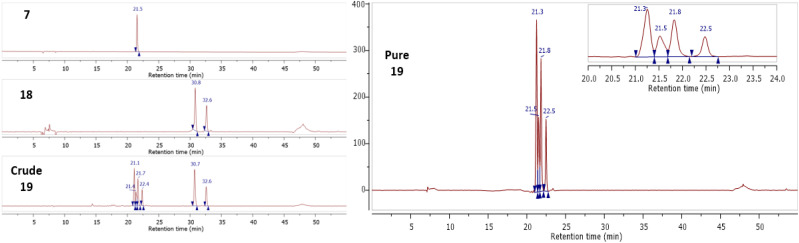
HPLC traces for 7, 18, and crude 19 mixture (left) and purified clicked product 19 showing 4 major diastereoisomers (right).

Following the successful synthesis of Gd complexes 7 and 15, bio-orthogonal click reactions between a cyclic-RGD-norbornene conjugate 18 and Gd complexes 7 and 15 were attempted. The cyclic RGD peptide is known to be a cell-penetrating-peptide (CPP) which could allow it to act as a peptide shuttle for allowing the Gd complexes through the BBB. The cyclic-RGD-norbornene conjugate 18 was synthesised following a standard amide coupling reaction and product formation was confirmed by ES^+^ MS with 2 isomers detected *via* HPLC due to the *exo*/*endo* isomers of the norbornene (details in the ESI†). Complex 7 (0.2 mM) and 18 (1 mM) were mixed in HEPES buffer and acetonitrile (95 : 5 v/v) at 37 °C (Scheme 3). After 16 hours, the complete conversion of 7 was observed by HPLC, with the introduction of 4 new peaks, corresponding to 4 diastereoisomers of the clicked product 19, as observed for related literature examples.^44^ The clicked product 19 was successfully isolated *via* HPLC and confirmed by MALDI MS, with the expected molecular ion and Gd isotope pattern observed (Fig. 5). No molecular ion for 7 was observed in the mass spectrum, showing complete consumption of 7. From the UV-trace over time, the loss of the tetrazine absorption at 265 nm and the appearance of a new absorption at 310 nm is clearly observed (Fig. S73, ESI†). A similar click reaction was also attempted between Gd complex 15 and 18, however the expected clicked product was not observed by MS, with only isomers for the free-ligand clicked product 20 being observed (Scheme 3, details in the ESI†). It is clear that the Gd is being lost from the chelate during the reaction, most likely due to the reduced denticity of the 7-coordinate ligand in 15 and hence, reduced stability of the Gd complex. It appears that the loss of Gd occurs during the click reaction, as there is no difference in the retention time of the clicked product peaks in the crude sample compared to after HPLC purification. A second group of isomers were also observed in the click reaction between 15 and 18 with a slightly longer retention time, corresponding to the free-ligand clicked product 21 with a fully aromatised pyridazine ring (Scheme 3, details in the ESI†).^45^ It is unclear why this fully aromatised product is not observed for the clicked product 19, but it is clearly related to the Gd de-coordination.

**Fig. 6:**
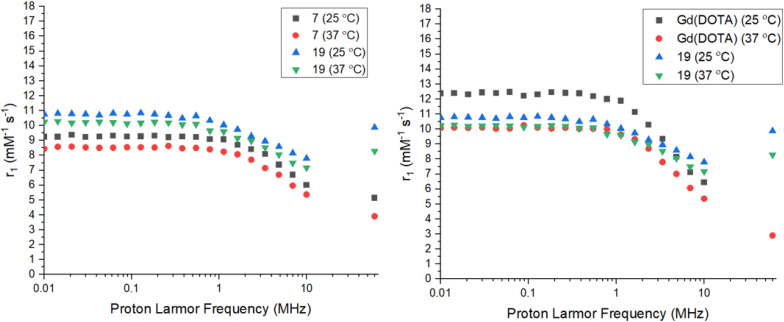
NMRD profiles for complex 7*vs.* clicked product 19 (left) and 19*vs.* Gd(DOTA) (right) and 25 and 37 °C.

The relaxivity of the clicked product 19 was assessed using the same methods as the Gd complexes 7 and 15 (Fig. 6 and Table 1). An enhanced relaxivity is observed for the clicked product 19 compared to the Gd-tetrazine complex 7 with a 1.3-fold increase at lower field strengths, most likely due to the increase in molecular weight and hence, reduced rotational correlation time (Table 1). At the clinically relevant frequency of 60 MHz, a larger 2-fold increase in relaxivity is observed compared to complex 7, with an increase of relaxivity observed beyond 10 MHz, which is typical for larger molecular weight probes with significantly reduced rotational correlation time.^46^ The clicked product 19 also shows higher relaxivity than the clinical standard of Gd(DOTA) at the clinically relevant frequency of 60 MHz at 37 °C with a near 3-fold increase in relaxivity of 8.29 compared to 2.90 mM^−1^ s^−1^. The pH dependent *r*_1_ relaxivity measurements for complex 7 and the clicked product 19 were also conducted to examine the performance of these potential contrast agents under various pH. For both the non-clicked complex 7 and the clicked product 19, their pH profiles are fairly flat over pH 2 to 12. No significant changes were observed in acidic or basic environments, indicating good stabilities in different pH conditions (Fig. S8, details in the ESI†). In addition, protein titrations with 4.5% HSA were also performed to mimic physiological conditions. Both complexes 7 and 19 gave significant enhancements with 21% and 13% increasement in their *r*_1_ relaxivities, respectively (Table S2, details in the ESI†). This shows there are some protein-complex interactions between the Gd-tetrazine complexes and the HSA proteins. These results highlight the excellent performance of both the Gd-tetrazine complex 7 and clicked product 19 under physiological conditions compared to Gd(DOTA).^41^

### *In vitro* cytotoxicity and cell imaging

**Fig. 7:**
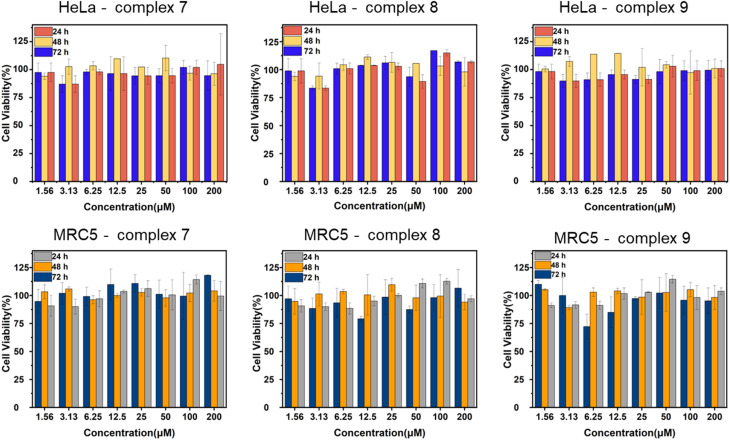
Cytotoxicty test results pf complexes 7, 8, 9 in HeLa and MRC5 cells at 24, 48, 72 h incubation.

**Fig. 8:**
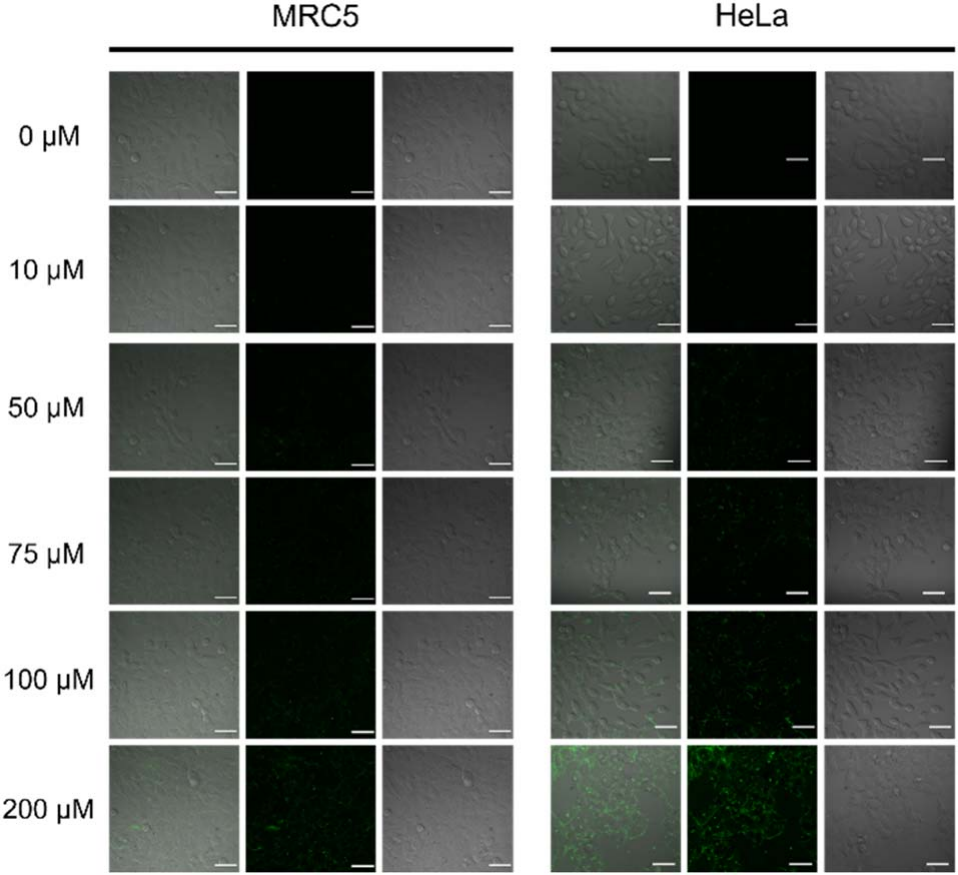
Confocal images of HeLa and MRC5 cells with complex 9 incubation for 24 h, *λ*_ex_ = 488 nm, scale bar = 50 μm.

The cytotoxicity of complexes 7, 8 and 9 have been studied in two cell lines: HeLa and MRC5 cells. The samples had been incubated of the complexes with the cells in 24, 48 and 72 h. Results showed that the three complexes showed no cytotoxic effects towards the two tested cell lines up to 200 μM (Fig. 7). Therefore, *in vitro* confocal imaging of complex 9 in the two cell lines was conducted. Since the safety of the complexes was illustrated up to 200 μM. After 24 h cell incubation with complex 9 of different concentrations, significant fluorescence signal was observed in the cell images starting from 75 μM upon 488 nm excitation which should be referred to the emission of Tb in complex 9 (Fig. 8).

## Conclusions

In conclusion, a series of Gd, Eu, and Tb tetrazine–lanthanide complexes were synthesised which could be used in bio-orthogonal click chemistry with peptide species for BBB penetration. The relaxivities of the Gd complexes were measured, with complex 7 showing higher values compared to Gd(DOTA) at the clinically relevant 1.4 T frequency and complex 15 demonstrating higher values than 7 or Gd(DOTA) at higher frequencies. The optical properties of the analogous Eu and Tb complexes were investigated, with standard tetrazine-based absorption and fluorescence observed, with the absorption and emission intensities varying depending on the tetrazine substituent. The Eu and Tb complexes demonstrated phosphorescence *via* tetrazine sensitization. The hydration numbers were also determined, with complexes 7–9 being *q* = 1 and complexes 15–17 being *q* = 2, as expected from their coordination geometries. A bio-orthogonal click reaction with Gd complex 7 and a cyclic-RGD-norbornene conjugate 18 was successful with the clicked probe 19 demonstrating enhanced relaxivity. The attempted click reaction between Gd complex 15 and norbornene 18 gave clicked products without Gd present, due to the reduced denticity and stability of the tetrazine ligand. In future work, the clicked probe 19 will have its BBB-penetration assessed to determine if the cyclic RGD peptide can act as a peptide shuttle to carry the Gd MRI agent through the BBB. The tetrazine probes will also be clicked to other BBB-peptide-delivery species, such as stapled peptides, and their ability to allow a large molecular weight MRI agent through the BBB assessed.

## Data availability

I can confirm that all the relevant research data is contained with the manuscript and ESI.† No databases have been used and no references to such databases are contained in the manuscript or ESI.†

## Author contributions

BW and YW synthesised the compounds. YW conducted the analysis and purification of the click reactions. BW performed the optical spectroscopy. BW, LX, JZ, and HFC performed the relaxivity measurements. KLW, GLL and NJL supervised the research and designed the experiments, and all the authors contributed to the preparation and writing the manuscript.

## Conflicts of interest

There are no conflicts to declare.

## Supplementary Material

SC-OLF-D4SC02335H-s001
