# Supplementary material for: Lanthanide–tetrazine probes for bio-imaging and click chemistry
Source: Chem Sci. 2025 Jan 22;16(8):3588–97. doi: 10.1039/d4sc02335h (PMC11759059; doi:10.1039/d4sc02335h)
Supplement: SC-016-D4SC02335H-s001 [file SC-016-D4SC02335H-s001.pdf]

Supplementary Information (SI) for Chemical Science.
This journal is © The Royal Society of Chemistry 2025

Supporting Information

Lanthanide-Tetrazine Probes for Bio-Imaging and Click Chemistry

Benjamin Woolley,[a] Yue Wu,[b] LI Xiong,[b] Ho-Fai Chau,[c] Junhui Zhang,[c] Ga-Lai Law,*[c] Ka-Leung Wong,*[c] and Nicholas Long*[a]

[a] Department of Chemistry, Imperial College London, Molecular Sciences Research Hub, 82 Wood Lane, White City Campus, London, W12 0BZ, UK.

[b] Department of Chemistry, Hong Kong Baptist University, Kowloon Tong, Hong Kong SAR, China.

[c] Department of Applied Biology and Chemical Technology, The Hong Kong Polytechnic University, Hung Hom, Hong Kong SAR, China.

Contents

1 - Experimental 2
1.1 - General: 2
1.2 - Absorption Spectroscopy 2
1.3 - Emission Spectroscopy 2
1.4 - Relaxivity Measurements: 2
2 - Synthetic Procedures: 3
3 - General Procedure for Metal Complexation Reactions: 11
4 - Relaxivity Data and NMRD Profiles 12
5 - UV-Vis Absorbance and Fluorescence Emission Spectra: 16
6 - Phosphorescence Emission Spectra: 21
7 - Lifetime and Hydration Number (q) Data: 22
7.1 - General Procedure for Calculating Hydration Numbers (q):[8] 23
8 - ^{1}H- and ^{13}C-NMR Spectra: 24
9 - Mass Spectra and LCMS Traces: 38
10 - Bio-orthogonal Click Reactions: 48
11 - References 52

1 - Experimental

1.1 - General:

All commercially available reagents were used as received from suppliers without further purification. Solvents used were laboratory grade. Thin-layer chromatography was performed on silica (Merk Art 5554) and visualised under UV radiation. Automated flash column chromatography was executed using a Biotage Isolera Four unit and KP-SIL silica cartridges (10 g or 25 g). ^{1}H (400 MHz) and $^{13}C\{H\}$ (100 MHz) NMR spectra were recorded on either a Bruker AV-500 or AV-400 spectrometer at room temperature at Imperial College London using standard pulse programs. Chemical shifts (δ) are quoted in parts per million (ppm) and referenced to the appropriate residual solvent signal, coupling constants in Hertz (Hz). Peak multiplicities are abbreviated as; s = singlet, m = multiplet, d = doublet, and br = broad. Mass spectrometry analysis (ES and APCI MS) was conducted by the Mass Spectrometry Service, Imperial College London. NMRD profiles were recorded using a SMARTracer 0.25 T (0.01-10 MHz) bench-top fast field cycling NMR relaxometer (King's College London-Imperial College London CDT in Smart Medical Imaging).

1.2 - Absorption Spectroscopy:

UV-visible absorption spectra were measured using an Agilent Technologies Cary 60 Spectrophotometer operating with WinUV software. The sample was held in a quartz cuvette with a path length of 1 cm. Absorption spectra were recorded against a baseline of pure solvent in an optically matched cuvette with a scan rate of 300 nm min^{-1} and a data interval of 0.5 nm. Extinction coefficients were calculated from the Beer Lambert Law (A=εcl) where A = the absorbance at a particular wavelength, ε is the extinction coefficient, c is the concentration, and l is the path length (width of the quartz cuvette, 1 cm).

1.3 - Emission Spectroscopy:

Fluorescence emission spectra were acquired on an Agilent Technologies Carry Eclipse Fluorescence Spectrophotometer, in quartz cuvettes with a path length of 1 cm, excitation and emissions slits set at 10 nm and with a scan rate of 600 nm min^{-1}. Fluorescence spectra were collected using 20 µM solutions in PBS buffer (pH 7.4). Emission spectra after excitation at the tetrazine fluorophore (265 nm) are presented with an axis break to remove $2\lambda_{ex}$ light from the fluorescence emission spectra for clarity. Phosphorescence spectra were recorded on an Agilent Technolgies Carry Eclipse Fluorescence Spectrophotometer in phosphorescence mode, in quartz cuvettes with a path length of 1 cm. Phosphorescence spectra were collected using 20 µM solutions in PBS buffer (pH 7.4) with the excitation and emission slits set at 10 nm, a gate time of 0.1 ms and delay time of 0.1 ms.

1.4 - Relaxivity Measurements:

The NMRD profiles were measured at 1H Larmor frequencies from 0.01 to 10 MHz using a 0.25 T Fast Field Cycling NMR relaxometer (SMARtracer, Stelar), equipped with a VTC90 temperature control unit. Each point was measured 4 times and the average value taken, with all values having percentage errors of less than 1%. The R_1 values were acquired at 25 °C and 37 °C and converted into r_1 values using the below equation, where R_1 is the observed relaxation rate, R_{1d} the diamagnetic constant of the solvent and [CA] the concentration of Gd^{3+} (determined using the Evans' Method).[1]

$$R_1 = r_1[CA] + R_{1d}$$

The data points at 60 MHz were acquired from the gradient of a T_1-inversion recovery experiment of R_1 against concentration of Gd^{3+} (determined either using the Evans' method or *via* ICPMS measurements). The relaxivity values for Gd(DOTA) for comparison from 0.01-10 MHz at 25 and 37

°C were obtained from Chabloz *et al*. while the relaxivity value at 60 MHz at 37 °C was obtained from Rohrer *et al*.[2,3]

1.5 – *In vitro* viability assay

The cytotoxicity of complexes were assessed by MTT viability assay. Cells (5 x 10^3 cells per well) were seeded onto 96-well plates and then incubated at 37 ^oC with 5% CO_2 in the dark for 24 h before adding samples. The cells were then incubated with samples for 24 h, 48h, and 72h. The medium was removed on the detection day, then incubated with 100 mL medium with 5% v/v Thiazolyl Blue (MTT) solution (5 mg/mL) at 37 ^oC for 3 h. 75 mL of solution was removed, and 125 mL of Dimethyl sulfoxide (DMSO) was added to dissolve the formazan crystals. The absorbance of the formazan crystal was measured at 540 nm and 650 nm by a dual-wavelength Azure microplate reader after 1h of shaking.

1.6 – *In vitro* cell confocal imaging

Cells (1 x 10^4 cells per well) were seeded onto confocal dishes and then incubated at 37 ^oC with 5% CO_2 in the dark for 24 h before adding samples. Complex are different concentrations (0, 10, 50, 75, 100 and 200 μM) were added into the cells with subsequent 24 h incubation. Before the imaging, the cells were washed with PBS and re-filled with culture medium for imaging. The confocal images were taken by Nikon AXR Laser Confocal Microscope.

2 - Synthetic Procedures:

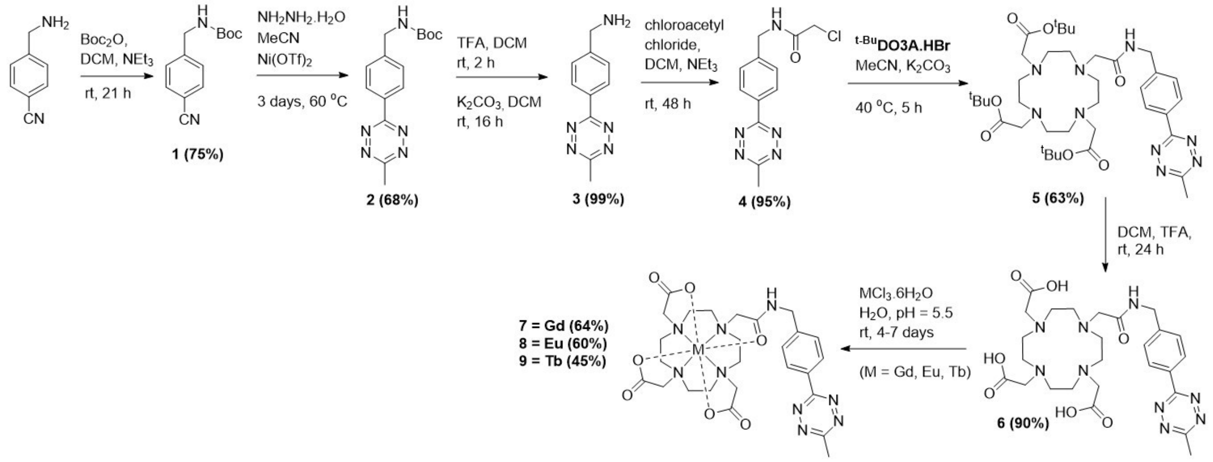

Scheme S1 - Reaction scheme for the synthesis of ***1-9****.*

1,4,7-Tris(*tert*-butoxycarbonylmethyl)-1,4,7,10-tetraazacyclododecane hydrobromide ($^{t\text{-Bu}}$DO3A.HBr):[4]

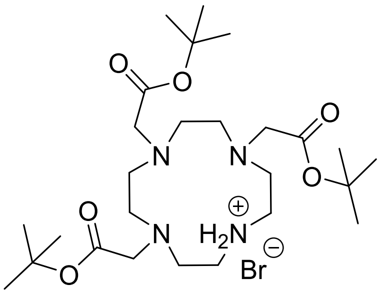

$^{t\text{-Bu}}$DO3A.HBr

To a suspension of cyclen (4.95 g, 29 mmol, 1 eq) and sodium acetate (7.87 g, 96 mmol, 3.3 eq) in *N,N*-dimethylacetamide (60 mL) at -20 °C, a solution of *tert*-butyl bromoacetate (18.9 g, 96 mmol, 3.3 eq) in *N,N*-dimethylacetamide (20 mL) was added dropwise over 30 minutes. The reaction mixture was then allowed to warm to room temperature and stirred for 24 hours. The reaction mixture was then poured into water (300 mL) and solid potassium hydrogen carbonate (15.00 g, 150 mmol, eq) was added portion wise. The resulting white precipitate was collected *via* filtration and redissolved in chloroform (250 mL). The clear solution was washed with water (100 mL), dried over sodium sulphate, and concentrated *in vacuo*. Diethyl ether (250 mL) was then added and the product precipitated out as a white fluffy solid, which was collected *via* filtration (14.2 g, 24 mmol, 83%).

^{1}H-NMR (400 MHz, $CDCl_3$) δ_H (ppm): 1.45 (9H, s), 1.46 (18H, s), 2.84-2.96 (12H, br m), 3.08-3.12 (4H, br m), 3.29 (s, 2H), 3.38 (s, 4H), 10.06 (1H, br s). ^{13}C-NMR (100 MHz, $CDCl_3$) δ_C (ppm): 28.3, 28.4, 47.7, 48.8 49.3, 51.3, 51.4, 58.3, 81.8, 81.9, 169.8, 170.7. HRMS (ES$^+$): Anal. For $C_{26}H_{51}N_4O_6^{+1}$(M^++1) Calcd.: 515.3809, Found: 515.3782.

Tert-butyl (4-cyanobenzyl)carbamate (1):[5]

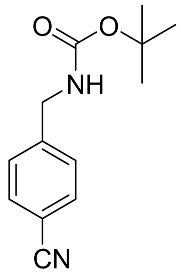

1

4-(Aminomethyl)benzonitrile hydrochloride (5.00 g, 30 mmol, 1 eq) was dissolved in dichloromethane (5 mL) and triethylamine (11 mL, 75 mmol, 2.5 eq). Di-*tert*-butyl dicarbonate (7.10 g, 33 mmol, 1.1 eq) was then added and the resultant mixture stirred at room temperature for 21 hours. The solvent was removed *in vacuo* before water (100 mL) was added and the organic phase extracted with dichloromethane (3 x 100 mL). The combined organic layers were then washed with water (2 x 100 mL) and the solvent removed *in vacuo* to yield the product **1** as a white powder (5.28 g, 23 mmol, 75%).

^{1}H-NMR (400 MHz, $CDCl_3$) δ_H (ppm): 1.45 (9H, s), 4.36 (2H, d, $^3J_{HH}$ = 6.1 Hz), 4.99 (1H, br s), 7.38 (2H, d, $^3J_{HH}$ = 8.4 Hz), 7.61 (2H, d, $^3J_{HH}$ = 8.4 Hz). ^{13}C-NMR (100 MHz, $CDCl_3$) δ_C (ppm): 28.5, 44.3, 80.2, 111.2, 118.9, 127.9, 132.5, 144.8, 156.0. HRMS (ES$^+$): Anal. For $C_{13}H_{17}N_2O_2^{+1}$(M^++1) Calcd.: 233.1290, Found: 233.1294.

Tert-butyl (4-(6-methyl-1,2,4,5-tetrazin-3-yl)benzyl)carbamate (2):[6]

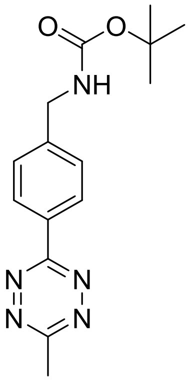

2

1 (0.60 g, 2.58 mmol, 0.1 eq), acetonitrile (1350 µL, 25.8 mmol, 1 eq), nickel(II) trifluoromethanesulfonate (0.46 g, 1.29 mmol, 0.05 eq), and hydrazine monohydrate (6.3 mL, 129 mmol, 5 eq) were added to a high pressure reaction tube which was sealed and heated at 60 °C for 72 hours. The solution was allowed to cool and water (10 mL) and sodium nitrite (3.56 g, 51.7 mmol, 2 eq) were added. Hydrochloric acid (1 M) was added dropwise to the mixture until the pH reached 3 and gases has stopped evolving. The bright red mixture was then extracted with ethyl acetate (3 x 50 mL), and the combined organic layers were washed with water (3 x 40 mL) and dried over sodium sulphate. The solvent was then removed *in vacuo* to yield a bright pink crude solid which was subsequently purified by silica column chromatography (hexane/ethyl acetate 2:1) to yield the product **2** as a bright pink solid (0.53 g, 1.75 mmol, 68%).

^{1}H-NMR (400 MHz, $CDCl_3$) δ_H (ppm): 1.48 (9H, s), 3.10 (3H, s), 4.44 (2H, d, J_{HH} = 5.8 Hz), 4.97 (1H, br s), 7.50 (2H, d, $^3J_{HH}$ = 8.4 Hz), 8.56 (2H, d, $^3J_{HH}$ = 8.4 Hz). ^{13}C-NMR (100 MHz, $CDCl_3$) δ_C (ppm): 21.3, 28.5, 44.5, 79.9, 128.2, 128.3, 130.9, 144.0 156.1, 164.0, 167.3. HRMS (APCI): Anal. For $C_{15}H_{19}N_5O_2^{+1}$(M^++1) Calcd.: 302.1617, Found: 302.1627.

(4-(6-Methyl-1,2,4,5-tetrazin-3-yl)phenyl)methanamine (3):

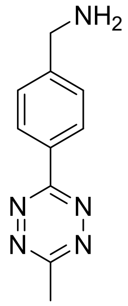

3

2 (199 mg, 0.66 mmol, 1 eq) was dissolved in a mixture of dichloromethane and trifluoroacetic acid (1:1 v/v, 5 mL) and left to stir at room temperature for 2 hours. The reaction mixture was then concentrated *in vacuo* before being redissolved in a mixture of dichloromethane and saturated potassium carbonate (1:1 v/v, 20 mL) and vigorously stirred for 16 hours. The organic phase was then extracted with dichloromethane (3 x 20 mL) before the organic layers were combined and dried over sodium sulphate. The solvent was then removed *in vacuo* to yield **3** as a bright pink solid (132 mg, 0.66 mmol, 99%).

^{1}H-NMR (400 MHz, $CDCl_3$) δ_H (ppm): 3.09 (3H, s), 4.01 (2H, s), 7.55 (2H, d, $^3J_{HH}$ = 8.4 Hz), 8.57 (2H, d, $^3J_{HH}$ = 8.4 Hz). ^{13}C-NMR (100 MHz, $CDCl_3$) δ_C (ppm): 21.3, 46.4, 128.0, 128.3, 130.5, 148.3, 164.2, 167.3. HRMS (ES$^+$): Anal. For $C_{10}H_{12}N_5^{+1}$(M$^+$+1) Calcd.: 202.1093, Found: 202.1098

2-Chloro-*N*-(4-(6-methyl-1,2,4,5-tetrazin-3-yl)benzyl)acetamide (4):

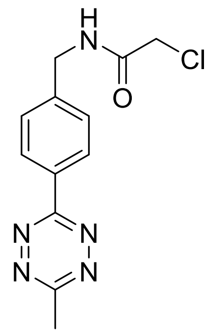

4

3 (106 mg, 0.53 mmol, 1 eq) and triethylamine (0.18 mL, 1.32 mmol, 2.5 eq) were dissolved in dichloromethane (15 mL) at room temperature under a nitrogen atmosphere before being cooled to -10 °C. Chloroacetyl chloride (50 µL, 0.63 mmol, 1.2 eq) was added and the pink solution allowed to warm to room temperature. After 16 hours, the pink solution was washed with water (3 x 10 mL) and dried over sodium sulphate. After being filtered, the solvent was removed *in vacuo* to yield the product **4** as a bright pink solid (140 mg, 0.50 mmol, 95%).

^{1}H-NMR (400 MHz, $CDCl_3$) δ_H (ppm): 3.10 (3H, s), 4.16 (2H, s), 4.63 (2H, d, J_{HH} = 6.0 Hz), 7.00 (1H, br s) 7.52 (2H, d, $^3J_{HH}$ = 8.4 Hz), 8.59 (2H, d, $^3J_{HH}$ = 8.4 Hz). ^{13}C-NMR (100 MHz, $CDCl_3$) δ_C (ppm): 21.3, 42.8, 43.6, 128.5, 128.6, 131.5, 142.2, 163.8, 166.2, 167.5. HRMS (ES$^+$): Anal. For $C_{12}H_{13}N_5OCl^{+1}$(M$^+$+1) Calcd.: 277.0730, Found: 319.1068 (M$^+$+MeCN+H).

Tri-*tert*-butyl 2,2',2''-(10-(2-((4-(6-methyl-1,2,4,5-tetrazin-3-yl)benzyl)amino)-2-oxoethyl)-1,4,7,10-tetraazacyclododecane-1,4,7-triyl)triacetate (5):

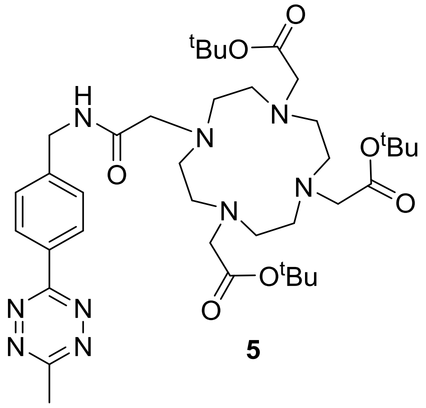

5

4 (0.16 g, mmol, 1 eq), **$^{t-Bu}$DO3A.HBr** (0.35 g, mmol, 1 eq) and potassium carbonate (0.33 g, mmol, 4 eq) were dissolved in acetonitrile (20 mL) and stirred at 50 °C. After 4 hours, the solution was allowed to cool and the inorganic salts were filtered before the solvent was removed *in vacuo* to give a dark pink solid. Purification was achieved via silica column chromatography (dichloromethane/methanol from 0 to 20%) to give the product **5** as a red solid (0.28 g, mmol, 63%).

^{1}H-NMR (400 MHz, $CDCl_3$) δ_H (ppm): 1.45 (18H, s), 1.46 (9H, s), 2.82 (4H, br s), 3.02 (8H, br s), 3.08 (3H, s), 3.35 (4H, br s), 3.78 (2H, s), 4.52 (2H br s), 4.56 (2H, d, $^3J_{HH}$ = 6.1 Hz), 7.61 (2H, d, $^3J_{HH}$ = 8.0

Hz), 8.53 (2H, d, $^3J_{HH}$ = 8.0 Hz). ^{13}C-NMR (100 MHz, $CDCl_3$) δ_C (ppm): 21.2, 28.0, 28.1, 28.3, 42.8, 47.7, 49.3, 51.5, 53.6, 55.8, 56.3, 58.4, 81.8, 82.1, 127.9, 128.8, 129.8, 145.56, 164.3, 167.1, 170.7, 172.4 HRMS (ES$^+$): Anal. For $C_{38}H_{62}N_9O_7^{+1}$(M$^+$+1) Calcd.: 756.4772, Found: 756.4783

2,2',2''-(10-(2-((4-(6-Methyl-1,2,4,5-tetrazin-3-yl)benzyl)amino)-2-oxoethyl)-1,4,7,10-tetraazacyclododecane-1,4,7-triyl)triacetic acid (6):[7]

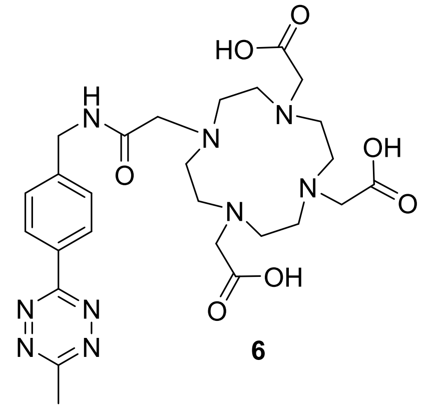

6

5 (0.24 g, 0.32 mmol, 1 eq) was dissolved in dichloromethane (10 mL) before trifluoroacetic acid (5 mL, excess) was added slowly and the resulting solution allowed to stir at room temperature, open to air. After 24 hours, the solvent was removed *in vacuo* to give a red oil, which was consecutively washed and reduced to dryness with dichloromethane (3 x 10 mL) and diethyl ether (3 x 10 mL) until a dark pink solid was obtained. Purification was achieved *via* reverse phase silica column chromatography (water/acetonitrile 0 to 95%) to give **6** as a red solid (0.17 g, 0.29 mmol, 90%).

^{1}H-NMR (400 MHz, D_2O) δ_H (ppm): 2.91 (3H, s), 2.98-3.02 (4H, br m), 3.17-3.20 (8H, m), 3.28-3.33 (4H, br m), 3.42 (4H, br s), 3.63 (4H, br s), 4.39 (2H, br s), 7.48 (2H, d, $^3J_{HH}$ = 8.2 Hz), 8.36 (2H, d, $^3J_{HH}$ = 8.2 Hz). ^{13}C-NMR (100 MHz, D_2O) δ_C (ppm): 20.0, 27.1, 42.2, 429, 47.7, 49.0, 51.8, 53.0, 55.0, 55.4, 128.2, 128.2, 130.4, 142.9, 164.0, 167.2, 169.3, 174.3. HRMS (ES$^+$): Anal. For $C_{26}H_{38}N_9O_7^{+1}$(M$^+$+1) Calcd.: 588.2894, Found: 588.2906

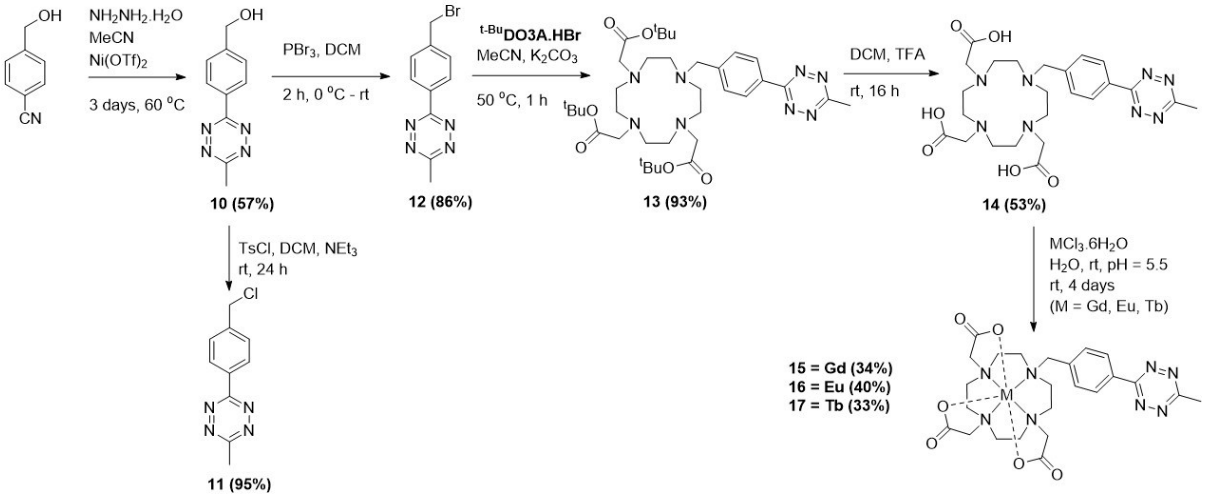

*Scheme S2 - Reaction scheme for the synthesis of **10-17**.*

(4-(6-Methyl-1,2,4,5-tetrazin-3-yl)phenyl)methanol (**10**):[6]

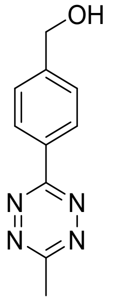

10

4-(Hydroxymethyl)benzonitrile (0.51 g, 3.83 mmol, 0.1 eq), acetonitrile (2.00 mL, 38.3 mmol, 1 eq), nickel(II) trifluoromethanesulfonate (0.68 g, 1.92 mmol, 0.05 eq), and hydrazine monohydrate (9.2 mL, 191 mmol, 5 eq) were added to a high pressure reaction tube which was sealed and heated at 60 °C for 72 hours. The solution was allowed to cool and water (10 mL) and sodium nitrite (5.25 g, 76.6 mmol, 2 eq) were added. Hydrochloric acid (1 M) was added dropwise to the mixture until the pH reached 3 and gases has stopped evolving. The bright pink mixture was then extracted with ethyl acetate (3 x 50 mL), and the combined organic layers were washed with water (3 x 40 mL) and dried over sodium sulphate. The solvent was then removed *in vacuo* to yield a dark pink crude solid which was subsequently purified by silica column chromatography (hexane/ethyl acetate 2:1) to yield the product **10** as a purple solid (0.44 g, 2.18 mmol, 57%).

^{1}H-NMR (400 MHz, $CDCl_3$) δ_H (ppm): 3.09 (3H, s), 4.83 (2H, d, J_{HH} = 5.8 Hz), 7.58 (2H, d, $^{3}J_{HH}$ = 8.4 Hz), 8.57 (2H, d, $^{3}J_{HH}$ = 8.4 Hz). ^{13}C-NMR (100 MHz, $CDCl_3$) δ_C (ppm): 21.3, 64.9, 127.5, 128.3, 131.1, 145.8, 164.1, 167.4. HRMS (ES$^+$): Anal. For $C_{10}H_{11}N_4O^{+1}$(M^++1) Calcd.: 203.0933, Found: 203.0941

3-(4-(Chloromethyl)phenyl)-6-methyl-1,2,4,5-tetrazine (**11**):

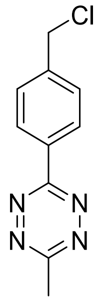

11

10 (75 mg, 0.37 mmol, 1 eq) and triethylamine (0.1 mL, 0.71 mmol, 1.9 eq) were dissolved in dichloromethane (10 mL) before p-toluenesulfonyl chloride (0.13 g, 0.67 mmol, 1.5 eq) was added. The resulting pink solution was allowed to stir at room temperature for 24 hours. The solvent was then removed *in vacuo* and the resulting pink solid purified by silica column chromatography (4:1 hexane/ethyl acetate) to yield **11** as a bright pink solid (78 mg, 0.35 mmol, 95%).

^{1}H-NMR (400 MHz, $CDCl_3$) δ_H (ppm): 3.11 (3H, s), 4.68 (2H, s), 7.62 (2H, d, $^{3}J_{HH}$ = 8.4 Hz), 8.60 (2H, d, $^{3}J_{HH}$ = 8.4 Hz). ^{13}C-NMR (100 MHz, $CDCl_3$) δ_C (ppm): 21.3, 45.6, 128.5, 129.5, 131.9, 142.1, 163.9, 167.5. HRMS (APCI): Anal. For $C_{10}H_{10}N_4Cl^{+1}$(M^++1) Calcd.: 221.0589, Found: 221.0591

3-(4-(Bromomethyl)phenyl)-6-methyl-1,2,4,5-tetrazine (12):

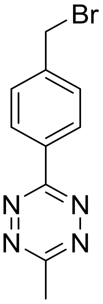

12

10 (0.12 g, 0.59 mmol, 1 eq) was dissolved in dichloromethane (4 mL) before PBr_3 (70 µL, 0.65 mmol, 1.1 eq) in dichloromethane (2 mL) was added dropwise at 0 °C. The resulting pink solution was then allowed to warm to room temperature. After 2 hours, the dark red solution was quenched with saturated sodium bicarbonate solution (20 mL), before the organic phase was washed with saturated sodium bicarbonate solution (2 x 20 mL), water (2 x 20 mL), and saturated sodium chloride solution (2 x 20 mL). The organic phase was dried over sodium sulphate and the solvent removed *in vacuo* to give **12** as a pink powder (0.13 g, 0.51 mmol, 86%).

^{1}H-NMR (400 MHz, $CDCl_3$) δ_H (ppm): 3.10 (3H, s), 4.57 (2H, s), 7.62 (2H, d, $^{3}J_{HH}$ = 7.7 Hz), 8.58 (2H, d, $^{3}J_{HH}$ = 7.7 Hz). ^{13}C-NMR (100 MHz, $CDCl_3$) δ_C (ppm): 21.3, 32.5, 128.5, 130.1, 131.9, 142.5, 163.8, 167.5. HRMS (APCI): Anal. For $C_{10}H_{10}N_4Br^{+1}$(M^++1) Calcd.: 265.0083, Found: 265.0083

Tri-*tert*-butyl 2,2',2''-(10-(4-(6-methyl-1,2,4,5-tetrazin-3-yl)benzyl)-1,4,7,10-tetraazacyclododecane-1,4,7-triyl)triacetate (13):

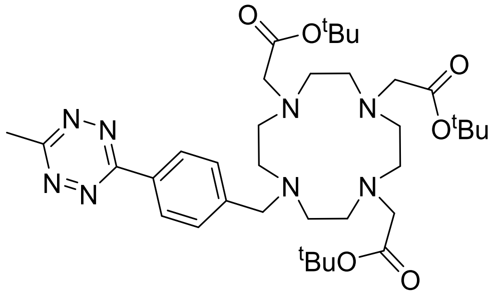

13

12 (87 mg, 0.33 mmol, 1 eq), ***t*-Bu**DO3A.HBr** (0.19 g, 0.33 mmol, 1 eq) and potassium carbonate (0.20 g, 1.44 mmol, 4 eq) were dissolved in MeCN (10 mL) and stirred at 50 °C. After 1 hour, the pink suspension had darkened, and the inorganic salts were filtered before the solvent was removed *in vacuo* to yield a red solid. The crude product was purified *via* silica column chromatography (dichloromethane/methanol 0 to 20%) to give **13** as a red sticky solid (0.21 g, 0.30 mmol, 93%).

^{1}H-NMR (400 MHz, $CDCl_3$) δ_H (ppm): 1.36 (18H, s), 1.40 (9H, s), 2.86-2.90 (4H, m), 2.96-3.00 (4H, m), 3.05 (3H, s), 3.16-3.20 (4H, m), 3.24-3.29 (4H, m), 3.36-3.40 (4H, m), 3.48 (2H, br s), 4.42 (2H, br s), 7.68 (2H, d, $^{3}J_{HH}$ = 8.0 Hz), 8.55 (2H, d, $^{3}J_{HH}$ = 8.0 Hz). ^{13}C-NMR (100 MHz, $CDCl_3$) δ_C (ppm): 21.2, 28.1, 28.2, 49.9, 50.5, 51.6, 55.7, 56.0, 57.6, 82.0, 82.4, 116.5, 128.3, 131.6, 132.2, 138.0, 163.6, 167,5, 169.1, 169.9. HRMS (ES^+): Anal. For $C_{36}H_{59}N_8O_6^{+1}$(M^++1) Calcd.: 699.4558. Found: 699.4557.

2,2',2''-(10-(4-(6-Methyl-1,2,4,5-tetrazin-3-yl)benzyl)-1,4,7,10-tetraazacyclododecane-1,4,7-triyl)triacetic acid (14):

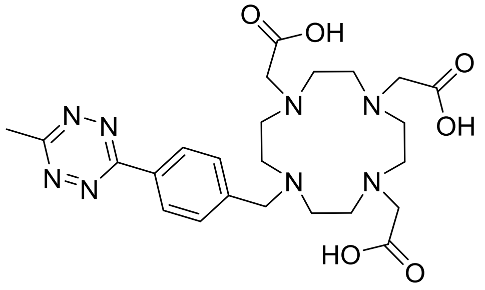

14

13 (0.20 g, 0.29 mmol, 1 eq) was dissolved in dichloromethane (5 mL) before trifluoroacetic acid (4 mL, excess) was added slowly and the resulting solution left to stir at room temperature, open to air. After 24 hours, the solvent was removed *in vacuo* to give a red oil, which was consecutively washed and reduced to dryness *in vacuo* with dichloromethane (3 x 5 mL) and diethyl ether (3 x 5 mL) to give a pink solid. Purification was achieved *via* reverse phase chromatography using a water/acetonitrile gradient (0 to 95%) to give **14** as a pink solid (80 mg, 0.15 mmol, 53%).

^{1}H-NMR (400 MHz, D_2O) δ_H (ppm): 3.06 (3H, s), 3.17-3.39 (20H, br m), 3.60-3.77 (6H, br m), 7.78 (2H, d, $^{3}J_{HH}$ = 8.1 Hz), 8.40 (2H, br s). ^{13}C-NMR (100 MHz, D_2O) δ_C (ppm): 20.1, 48.0, 48.6, 49.2, 51.2, 53.8, 57.0, 111.9, 114.8, 117.7, 128.8, 131.5, 162.8, 163.1, 163.8, 167.4. HRMS (ES$^+$): Anal. For $C_{24}H_{35}N_8O_6^{+1}$(M$^+$+1) Calcd.: 531.2680. Found: 531.2674.

Cyclic-RGD-Norbornene (18):

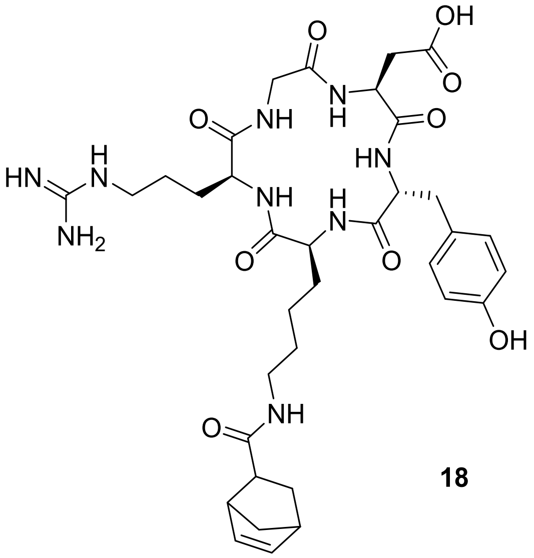

18

A mixture of cyclo(RGDyK) peptide (mono TFA salt, 30 mg, 0.04 mmol, 1 eq), 5-norbornene-2-carboxylic acid (mixture of *endo* and *exo*, predominantly *endo*, 14 mg, 0.1 mmol, 2.5 eq), PyBOP (52 mg, 0.1 mmol, 2.5 eq) and DIPEA (35 µL, 0.2 mmol, 5 eq) in DMF (1 mL) was stirred at room temperature for 5 hours. The completion of the reaction was confirmed *via* analytical HPLC. The reaction was diluted with water before being purified *via* reverse phase column chromatography using a water/acetonitrile gradient (5 to 50%) to give **18** as a white solid (25 mg, 0.029 mmol, 73%).

HRMS (MALDI): Anal. For $C_{35}H_{50}N_9O_9^{+1}$ (M$^+$+1) Calcd.: 740.3726. Found: 740.3918.

3 - General Procedure for Metal Complexation Reactions:

Free ligand **6** or **14** (1 eq) and $MCl_3.6H_2O$ (1.1 eq, M = Gd, Eu, Tb) were dissolved in water (5 mL) and the pH adjusted to 5.5 with 0.1 M NaOH before being allowed to stir at room temperature. After 24 hours, an additional 1 equivalent of $MCl_3.6H_2O$ was added and the pH readjusted to 5.5. After a further 3 days the solvent was removed *in vacuo* to give a light pink solid. Purification was achieved *via* reverse phase column chromatography eluting with a water/acetonitrile gradient (0 to 95%, no TFA) to give the complexes as light pink solids following lyophilisation.

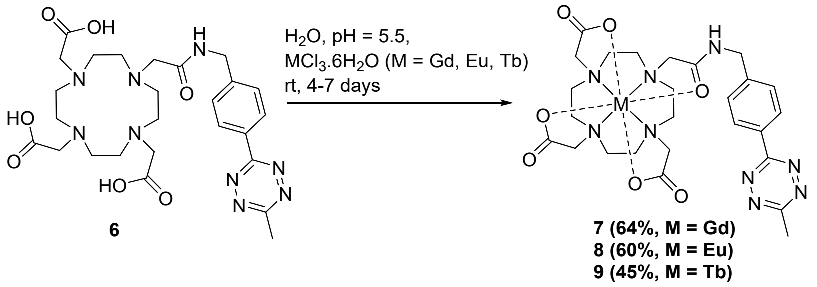

*Scheme S3 – General complexation procedure for free-ligand **6** to give lanthanide complexes **7-9**.*

7 (M = Gd) = 64%. HRMS (ES$^+$): Anal. For $C_{26}H_{35}N_9O_7Gd^{+1}$($M^+$+1) Calcd.: 743.1901 Found: 743.1915.

8 (M = Eu) = 60%. HRMS (ES$^+$): Anal. For $C_{26}H_{35}N_9O_7Eu^{+1}$($M^+$+1) Calcd.: 736.1858 Found: 736.1840.

^{1}H-NMR (400 MHz, D_2O) δ_H (ppm): -17.21, -16.94, -15.87, -15.51, - 14.87, -12.82, -12.46, -11.30, -10.78, -7.94, - 7.32, - 6.91, - 6.16, - 5.08, -3.92, -3.04, -2.29, -0.43, - 0.09, 1.25, 1.52, 1.91, 2.03, 2.18, 2.50, 3.32, 31.84, 31.99, 32.37, 33.33.

9 (M = Tb) = 45%. HRMS (ES$^+$): Anal. For $C_{26}H_{35}N_9O_7Tb^{+1}$($M^+$+1) Calcd.: 744.1913 Found: 744.1930.

^{1}H-NMR (400 MHz, D_2O) δ_H (ppm): -394.77, -382.17, -378.31, -372.25, -124.98, -120.58, -114.11, -111.15, -105.30, -79.05, -77.87, -76.54, -71.33, -1.13, 0.31, 1.51, 4.71, 7.36, 10.53, 18.22, 20.49, 27.90, 47.82, 63.58, 66.32, 108.71, 192.42, 202.08, 244.60, 247.63, 255.74

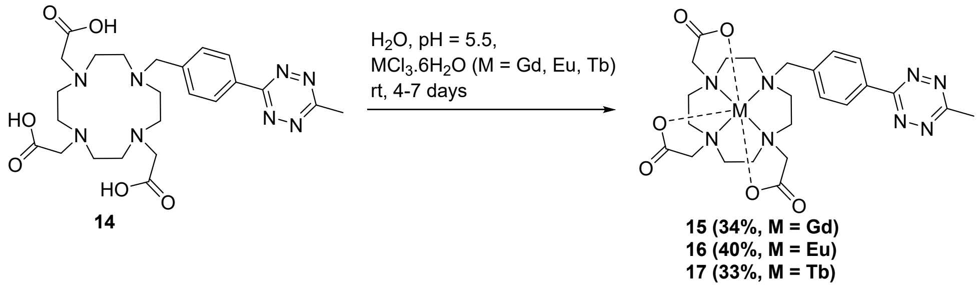

*Scheme S4 - General complexation procedure for the free-ligand **14** to give lanthanide complexes **15-17**.*

15 (M = Gd) = 34%. HRMS (ES$^+$): Anal. For $C_{24}H_{32}N_8O_6Gd^{+1}$($M^+$+1) Calcd.: 686.1686. Found: 686.1712.

16 (M = Eu) = 40%. HRMS (ES$^+$): Anal. For $C_{24}H_{32}N_8O_6Eu^{+1}$($M^+$+1) Calcd.: 679.1565. Found: 679.1545.

17 (M = Tb) = 33%. HRMS (ES$^+$): Anal. For $C_{24}H_{32}N_8O_6Tb^{+1}$($M^+$+1) Calcd.: 687.1698. Found: 687.1718.

4 - Relaxivity Data and NMRD Profiles

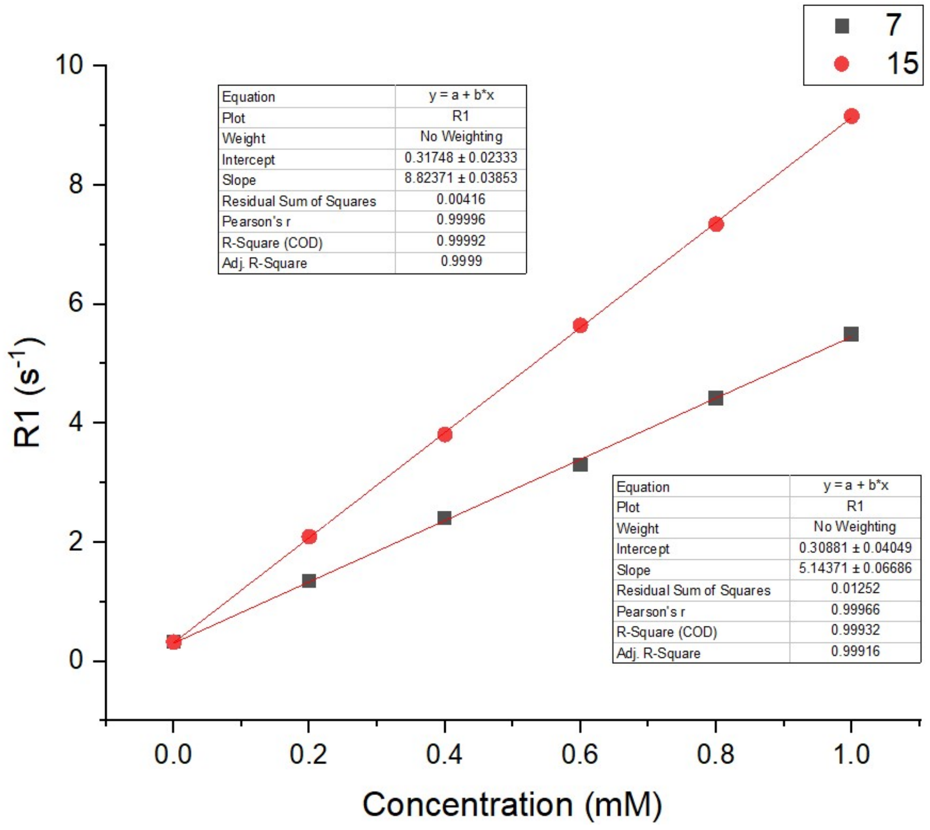

*Figure S1 - T_1-inversion recovery experiment for **7** and **15** (60 MHz, 25 °C).*

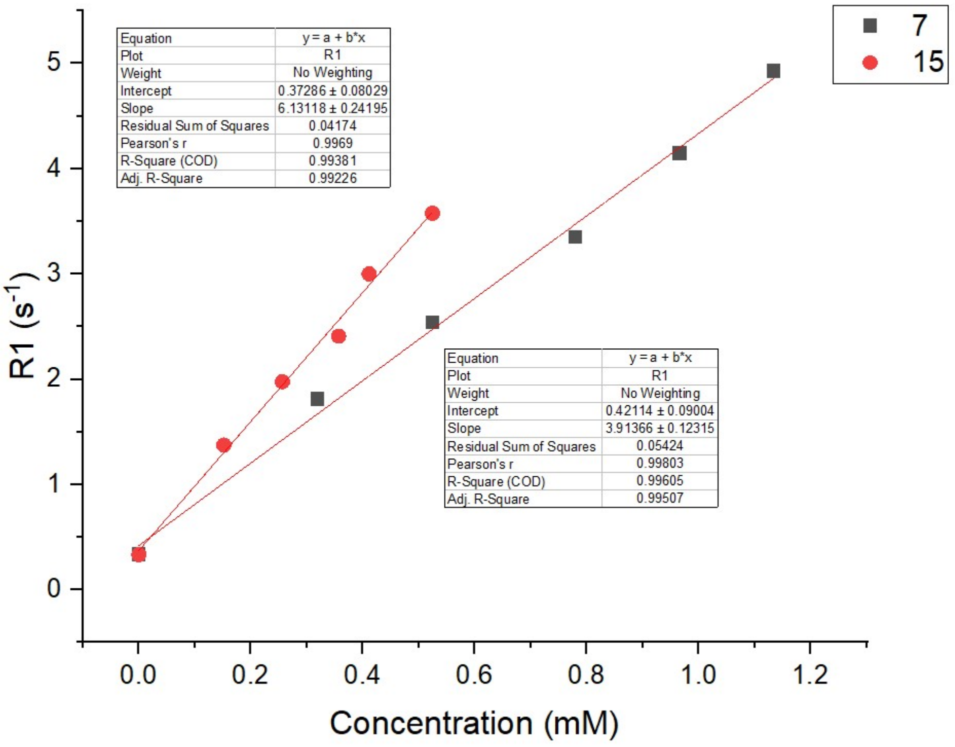

*Figure S2 - T_1-inversion recovery experiment for **7** and **15** (60 MHz, 37 °C).*

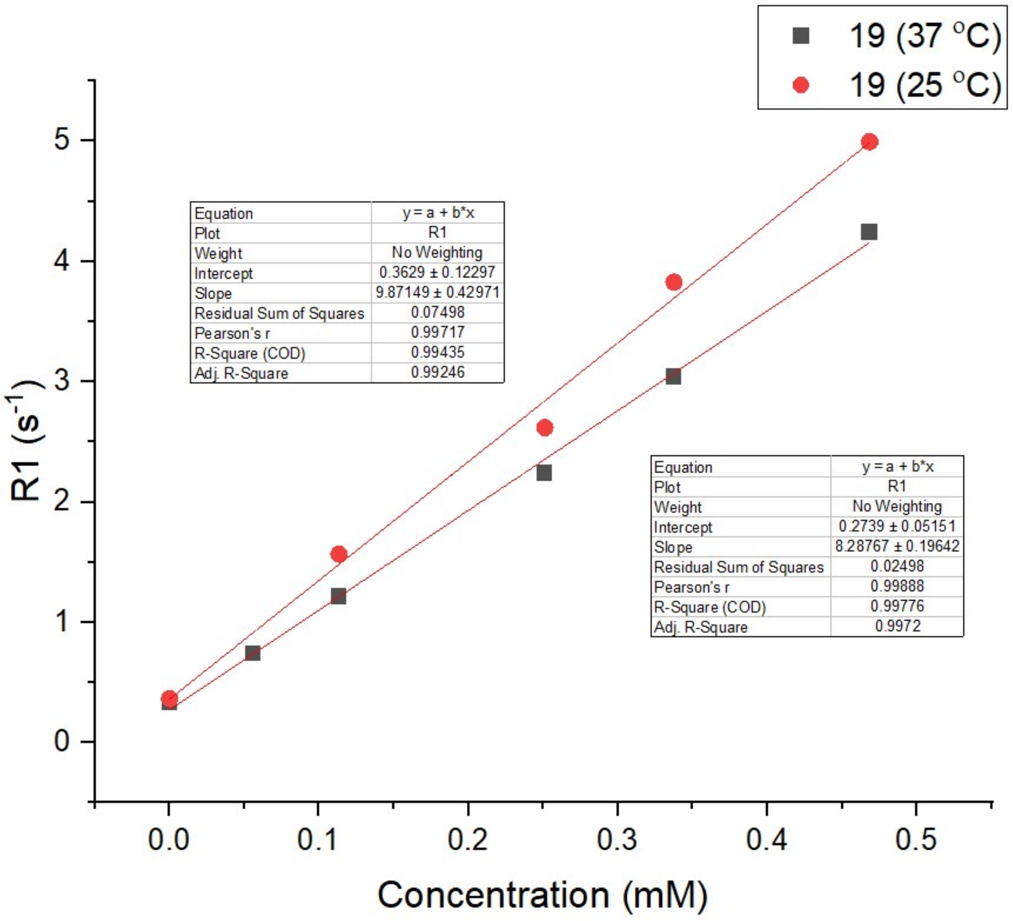

*Figure S3 - T_1-inversion recovery experiment for **19** at 25 and 37 °C (60 MHz)*

*Table S1 - Summary of relaxivity data for complexes **7**, **15**, **19** and Gd(DOTA).*[2,3]

Complex	r_1 0.01 MHz (mM^{-1} s^{-1})	r_1 10 MHz (mM^{-1} s^{-1})	r_1 60 MHz (mM^{-1} s^{-1})
**7 (25 °C)**	9.24	6.00	5.14
**7 (37 °C)**	8.44	5.36	3.91
**15 (25 °C)**	10.80	9.02	8.82
**15 (37 °C)**	9.18	7.53	6.13
**19 (25 °C)**	10.77	7.80	9.87
**19 (37 °C)**	10.24	7.18	8.29
**Gd(DOTA) (37 °C)**[2,3]	10.07	5.36	2.90

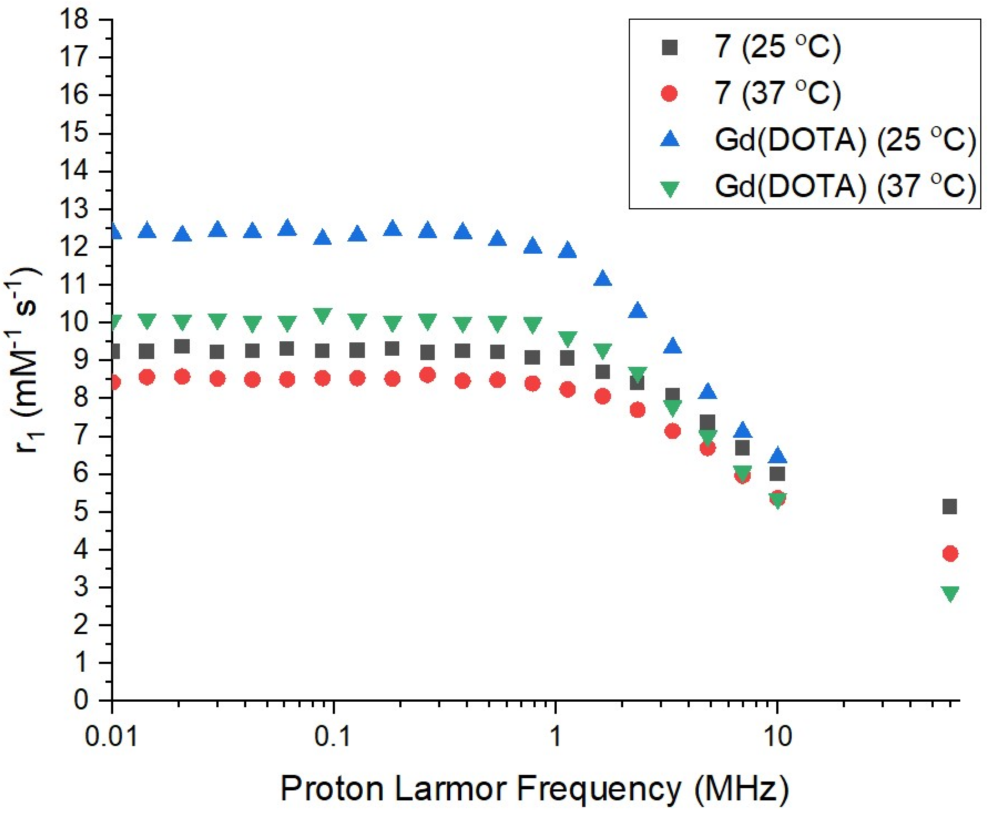

*Figure S4 - NMRD profile for **7** vs Gd(DOTA).*[2,3]

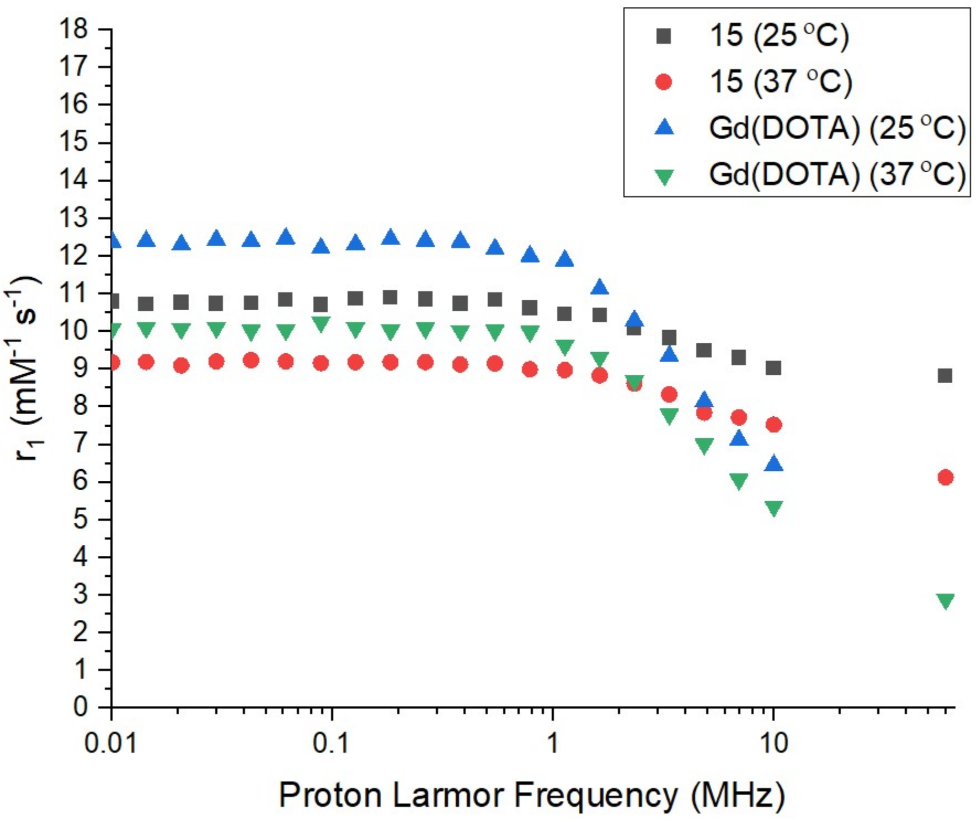

*Figure S5 - NMRD profile for **15** vs Gd(DOTA).*[2,3]

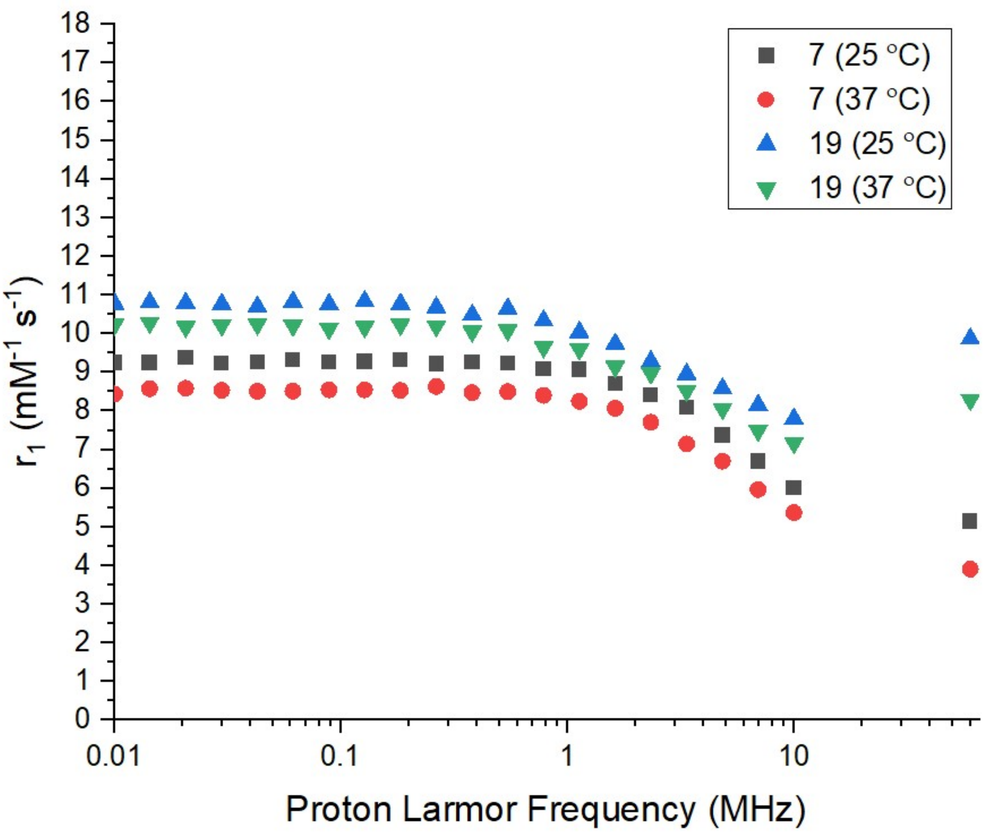

*Figure S6 - NMRD profile for **7** vs **19**.*

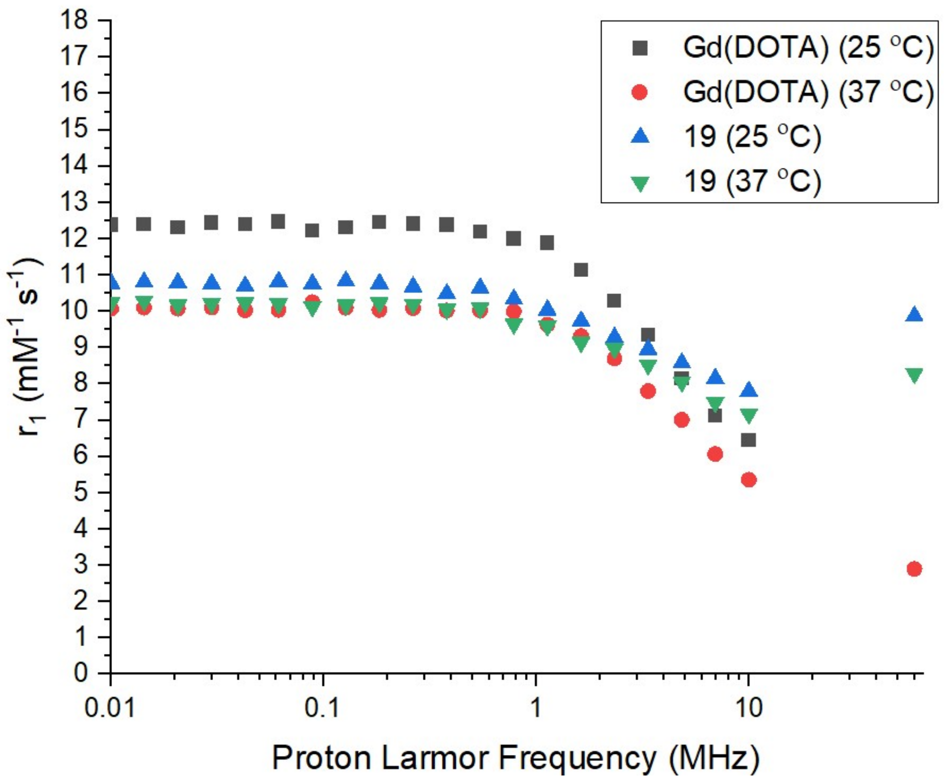

*Figure S7 - NMRD profile for **19** vs Gd(DOTA).*[2,3]

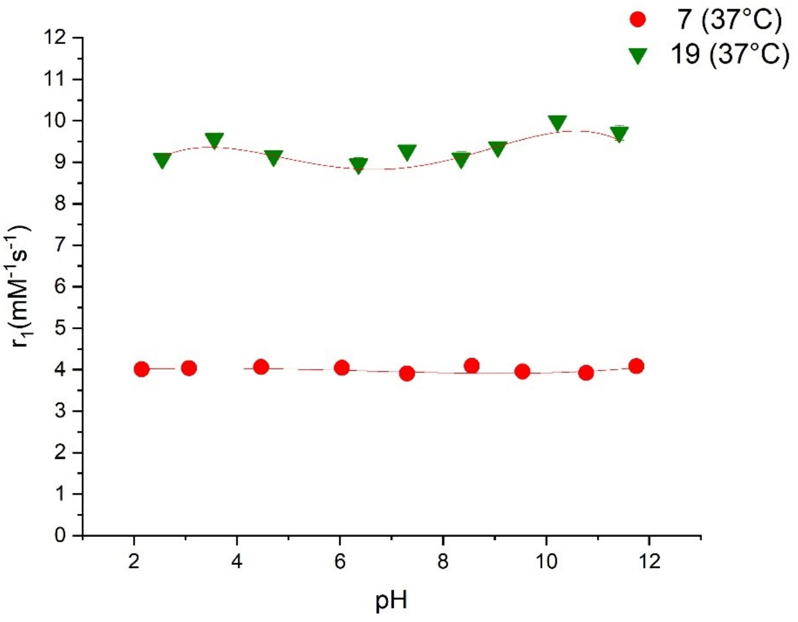

*Figure S8 – pH dependent r_1 relaxivity measurements for complex **7** and the clicked product **19** in water, pH was adjusted by 1 N NaOH and 1 N HCl.*

*Table S2 - r_1 relaxivity measurements for complex **7** and the clicked product **19** with and without 4.5% HSA at 37 °C, 60 MHz.*

Comple x	Without HSA (mM^{-1} s^{-1})	With HSA (mM^{-1} s^{-1})
**7**	3.90 ± 0.003	4.73 ± 0.01
**19**	9.28 ± 0.03	10.48 ± 0.01

5 - UV-Vis Absorbance and Fluorescence Emission Spectra:

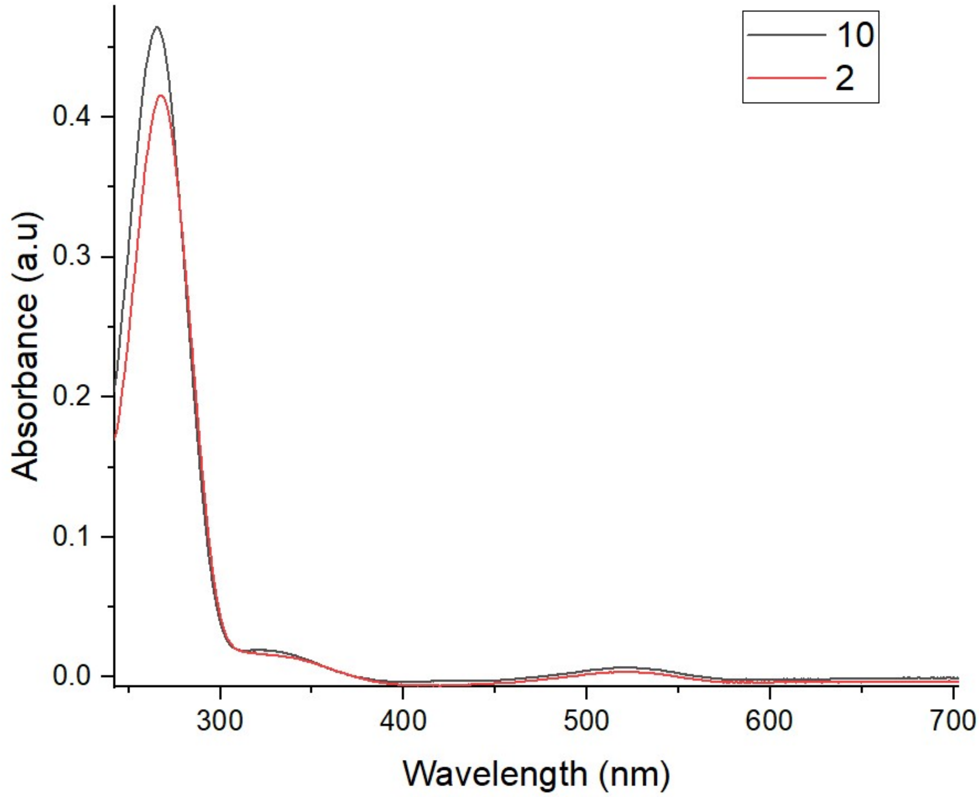

*Figure S9 - UV-vis absorbance spectra for tetrazines **2** and **10** (20 µM, PBS, slits 10 nm).*

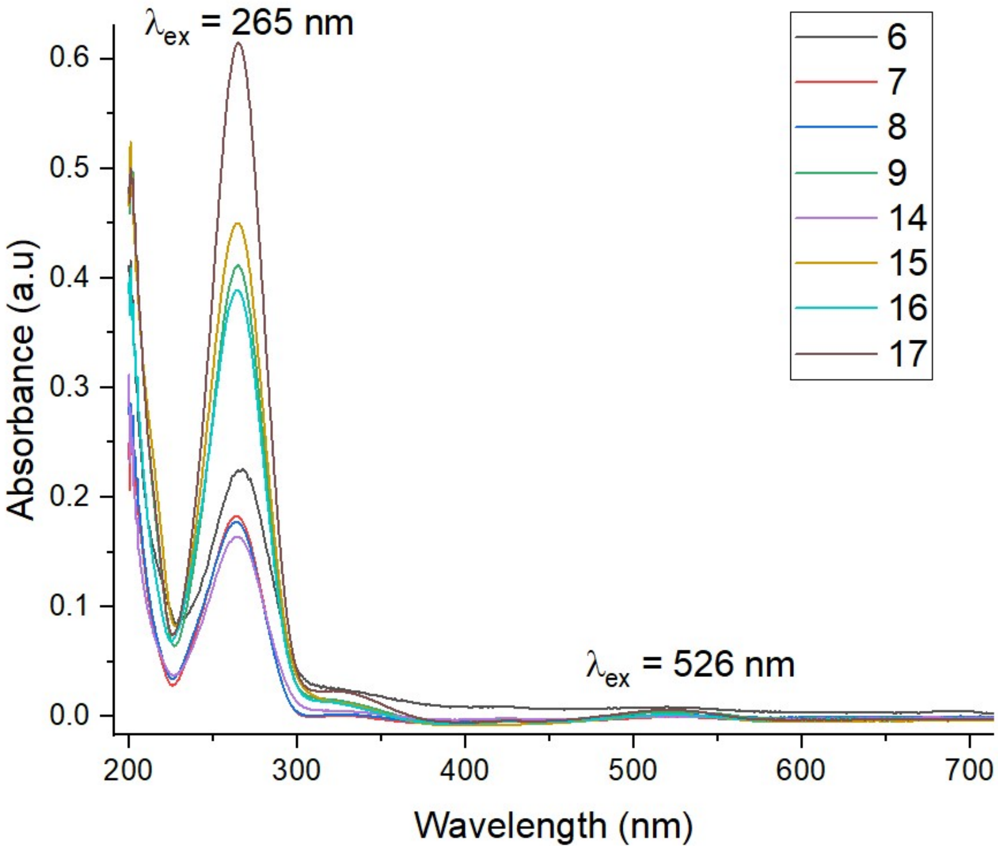

*Figure S10 - UV-vis absorbance spectra for **6-9** and **14-17** (20 µM, PBS, slits 10 nm).*

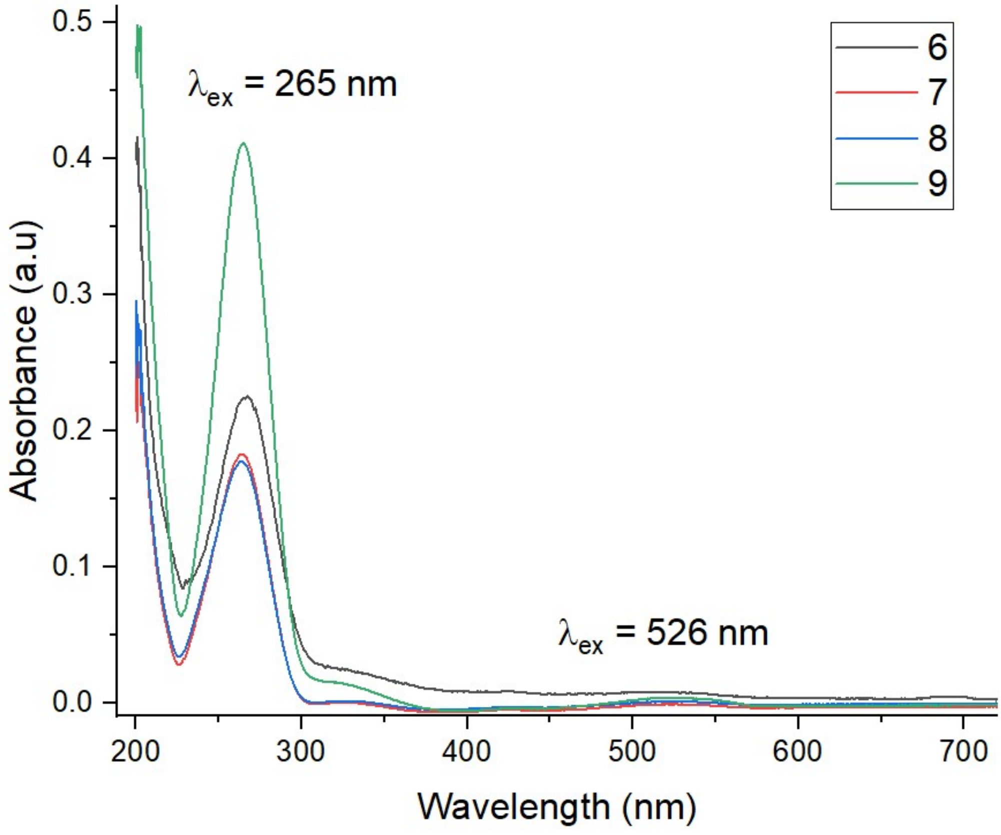

*Figure S11 - UV-vis absorbance spectra for **6-9** (20 µM, PBS, slits 10 nm).*

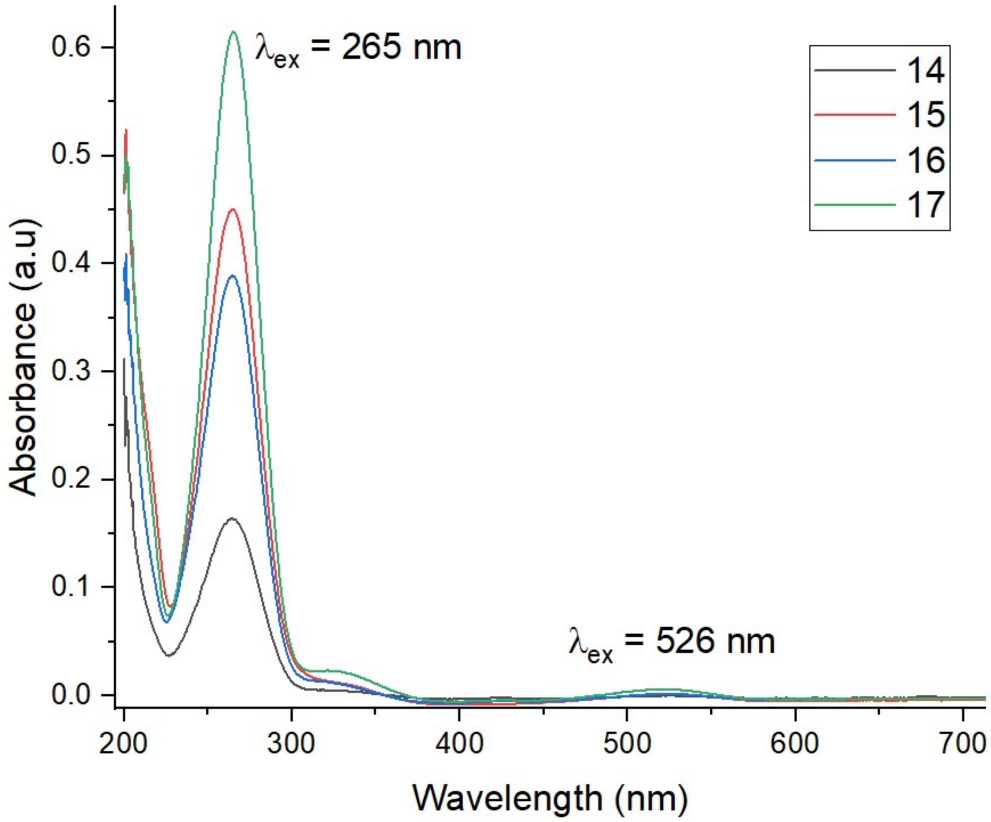

*Figure S12 - UV-vis absorbance spectra for **14-17** (20 µM, PBS, slits 10 nm).*

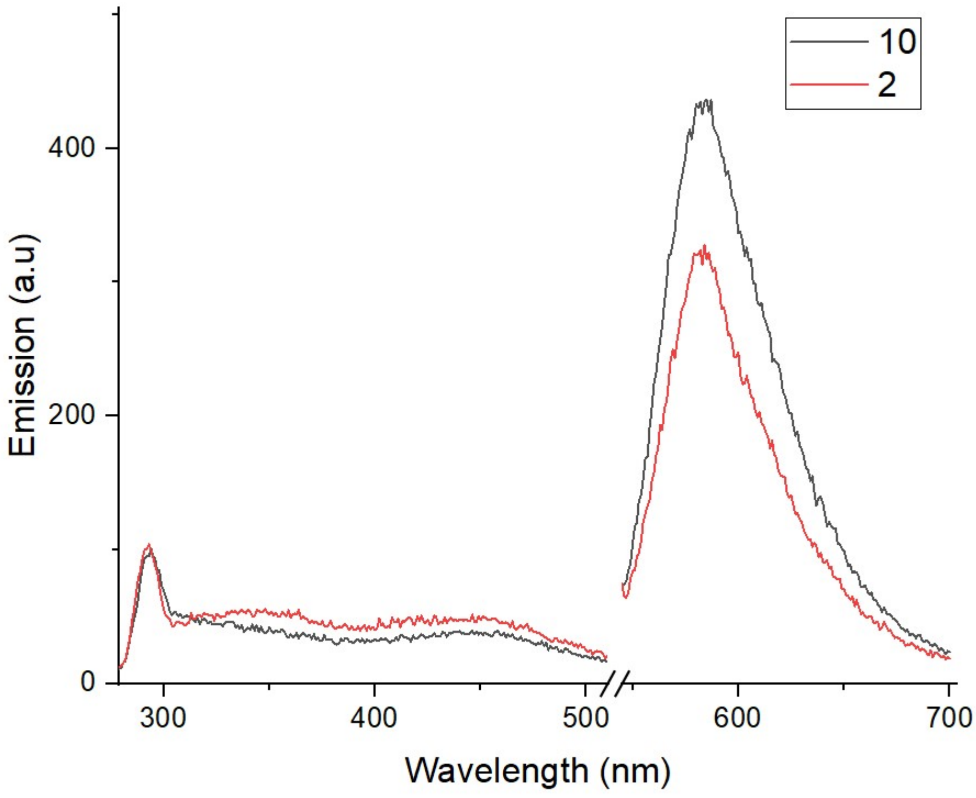

*Figure S13 - Fluorescence emission spectra for tetrazines **2** and **10** (20 µM, PBS, slits 10 nm, axis break to remove $2\lambda_{ex}$ peak).*

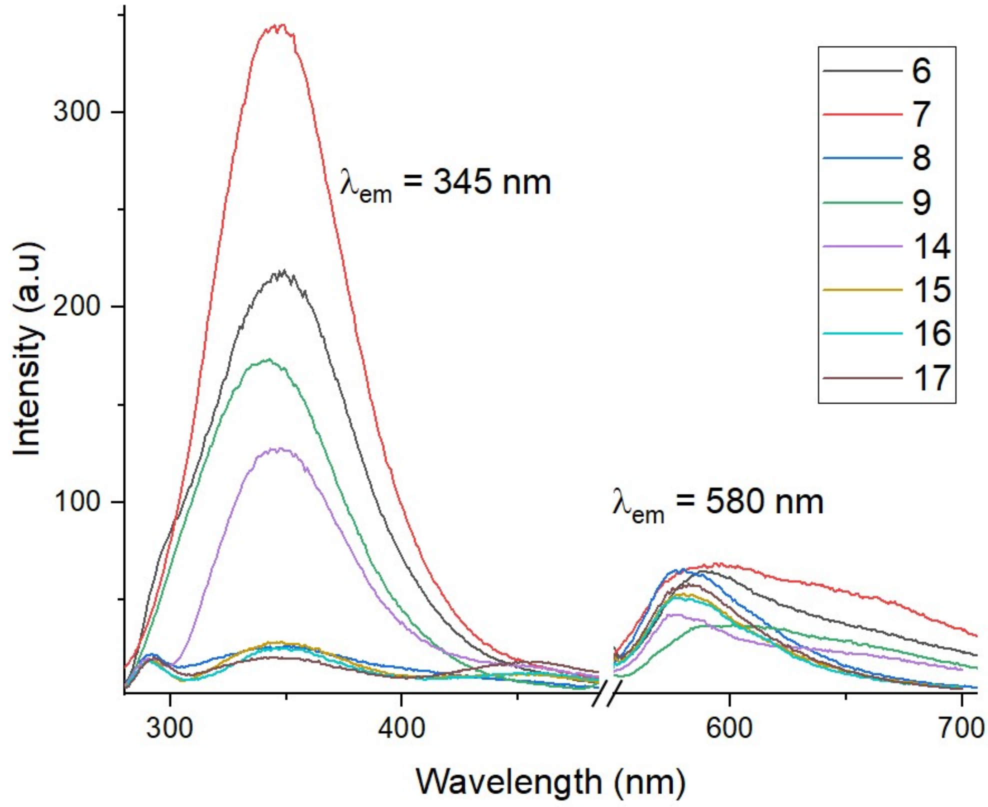

*Figure S14 - Fluorescence emission spectra for **6-9** and **14-17** (20 μM, PBS, slits 10 nm, axis break to remove 2λ_{ex} peak).*

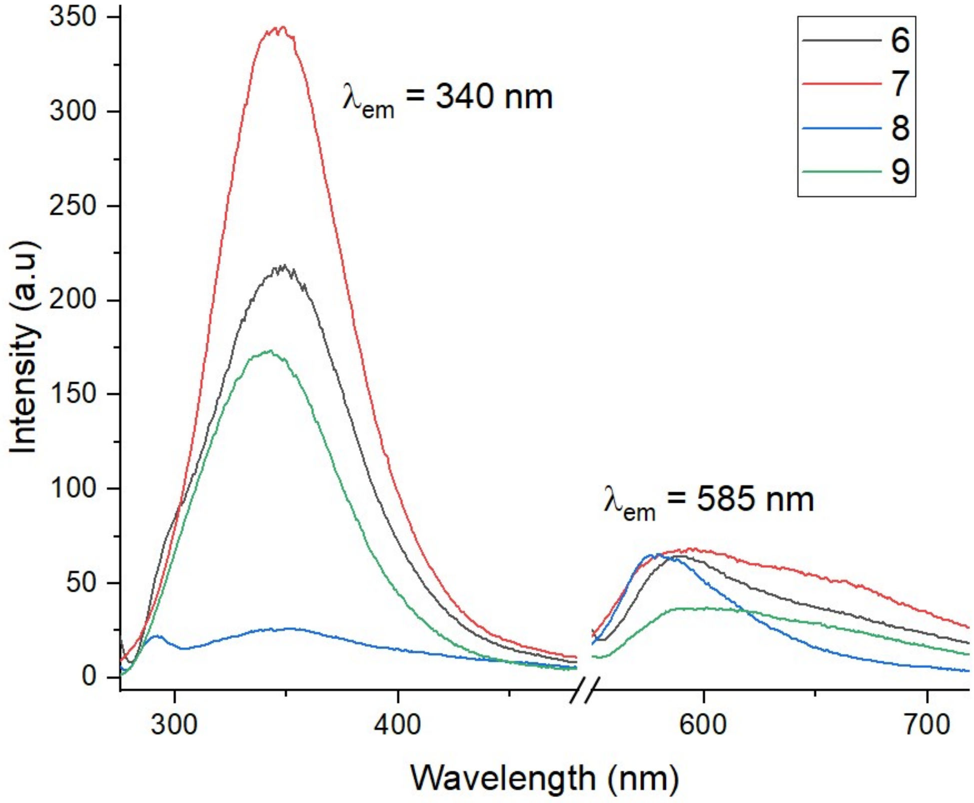

*Figure S15 - Fluorescence emission spectra for **6-9** (20 µM, PBS, slits 10 nm, axis break to remove $2\lambda_{ex}$ peak).*

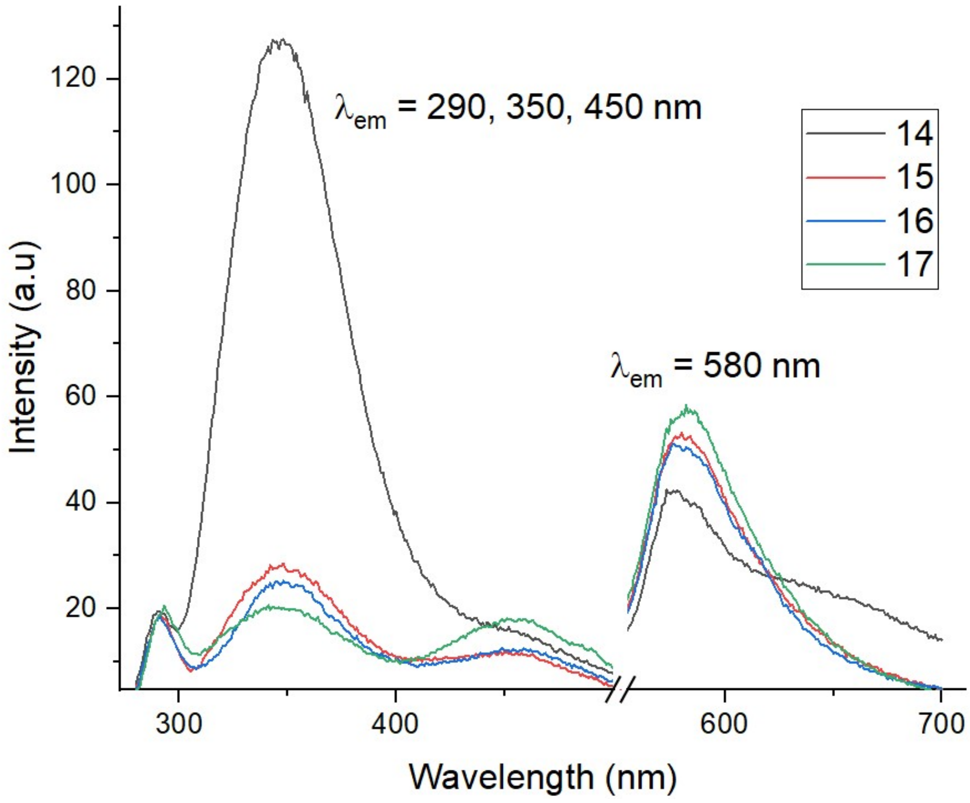

*Figure S16 - Fluorescence emission spectra for **14-17** (20 µM, PBS, slits 10 nm, axis break to remove $2\lambda_{ex}$ peak).*

*Table S3 - UV-vis absorbance, extinction coefficients, and fluorescence emission peaks for compounds **6-9** and **14-17** (20 µM, PBS, slits 10 nm).*

Compound	λ_{ex} (nm)	ε (M^{-1} cm^{-1})	Fluorescence λ_{em} (nm)
**2**	267	20790	293, 350, 450, 583
**6**	267	11200	347, 590
**7**	264	9100	347, 593
**8**	264	8750	291, 348, 579
**9**	265	20050	342, 590
**10**	265	23150	293, 350, 450, 583
**14**	264	8250	291, 347, 576
**15**	265	22500	291, 348, 458, 580
**16**	265	19450	291, 348, 458, 580
**17**	265	30650	291, 346, 458, 583

*Table S4 - Phosphorescence emission peaks for compounds **8-9** and **16-17** (20 µM, PBS, slits 10 nm, 0.1 ms delay).*

Compound	λ_{ex} (nm)	Phosphorescence λ_{em} (nm)
**8**	264	592, 616, 652, 698
**9**	265	490, 545, 586, 621, 650
**16**	265	592, 616, 652, 698
**17**	265	490, 545, 586, 621, 650

6 - Phosphorescence Emission Spectra:

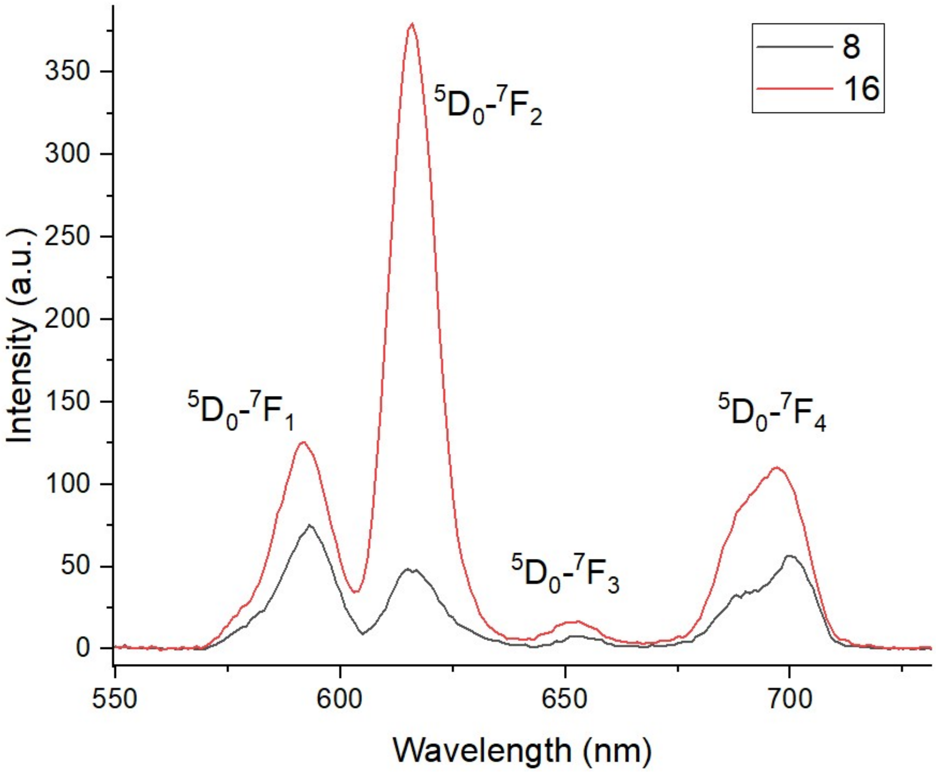

*Figure S17 - Phosphorescence emission spectra for Eu complexes **8** and **16** (20 µM, PBS, slits 10 nm, 0.1 ms delay).*

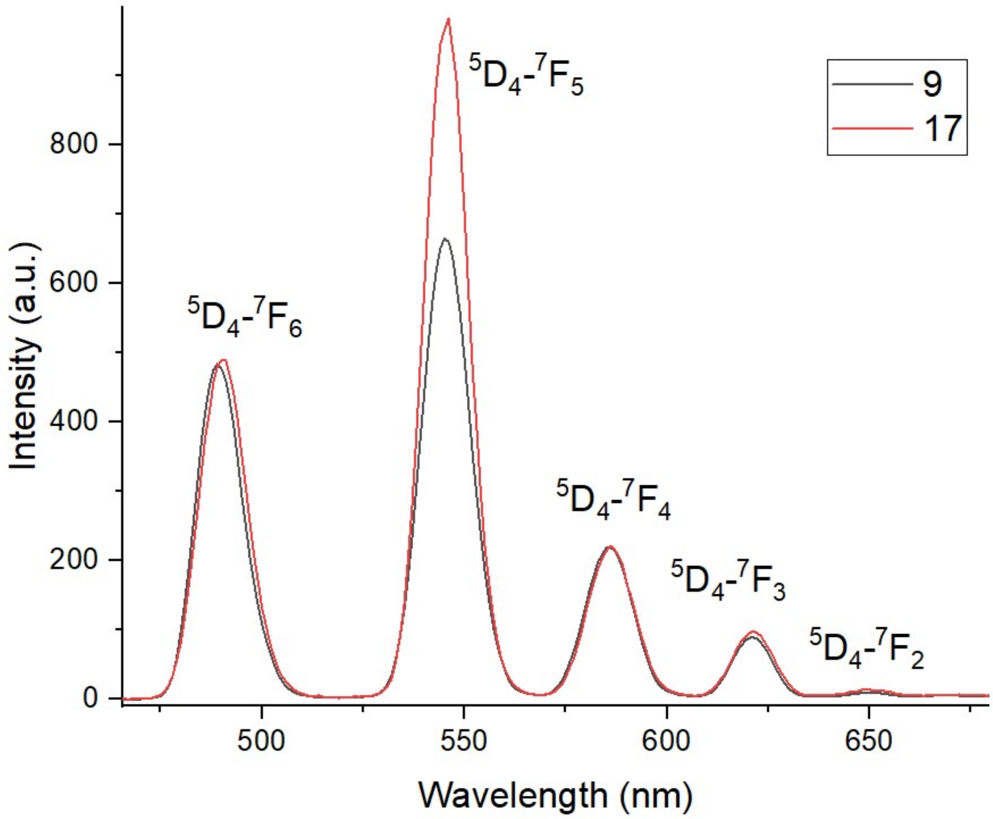

Figure S18 - Phosphorescence emission spectra for Tb complexes ***9*** *and* ***17*** *(20 µM, PBS, slits 10 nm, 0.1 ms delay).*

7 - Lifetime and Hydration Number (q) Data:

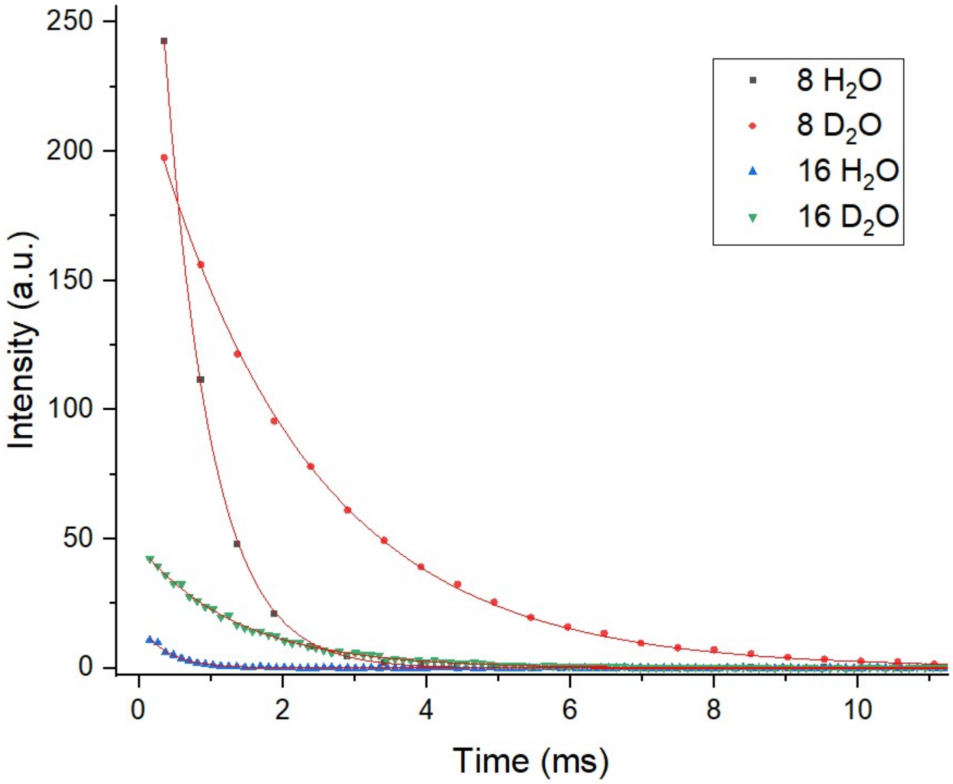

*Figure S19 - Eu complexes **8** and **16** decay lifetime emission spectra (20 µM, slits 10 nm, 0.1 ms delay).*

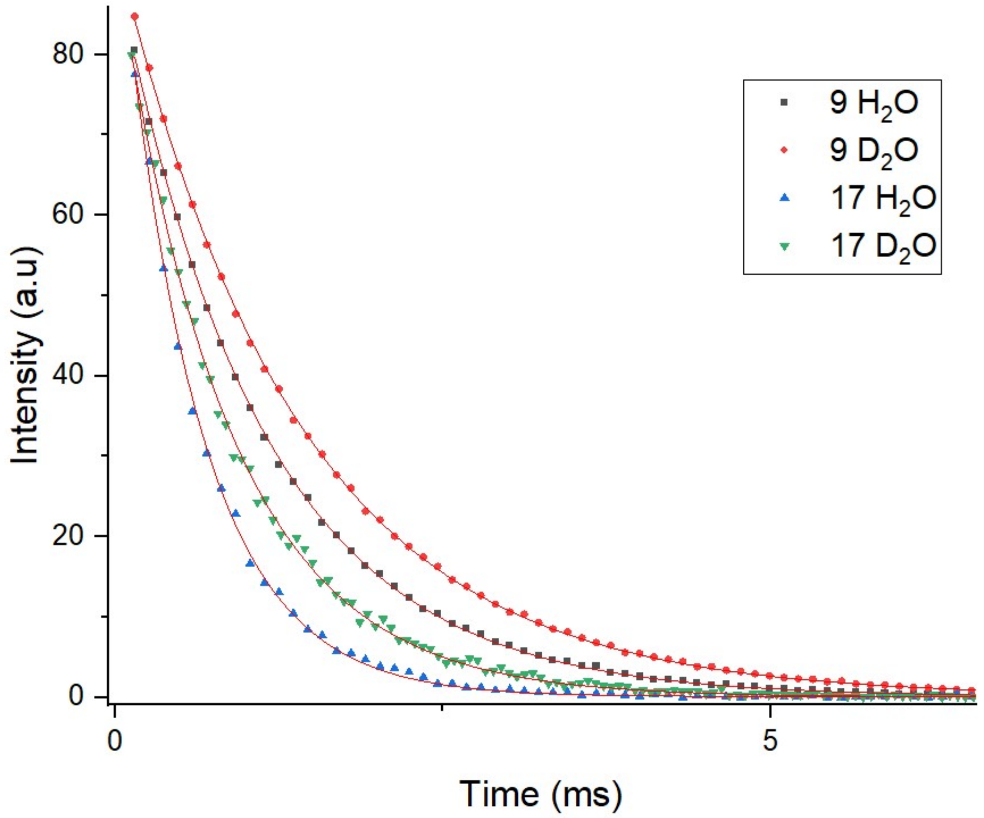

*Figure S20 - Tb complexes **9** and **17** decay lifetime emission spectra (20 µM, slits 10 nm, 0.1 ms delay).*

7.1 - General Procedure for Calculating Hydration Numbers (q):[8]

Lanthanide-based lifetimes were measured using a Varian Cary Eclipse spectrophotometer in the phosphorescence mode, using 20 µM solutions in water or deuterium oxide. The gate time was fixed at 0.1 ms with both excitation and emission slits set at 10 nm and 0.1 ms delay time. Solutions (20 µM) of Eu complexes **8** and **16** and Tb complexes **9** and **17** in either H_2O or D_2O were prepared. The decay of emission intensity of the most intense phosphorescent peak after excitation at 265 nm (either 593 or 615 nm for Eu complexes **8** and **16**, or 546 nm for the Tb complexes **9** and **17**) was monitored in each solvent and a graph of intensity against time plotted. The decay profiles for each system were fitted to a single exponential decay and the gradient represents the decay lifetime (τ):

$$I(t) = I(0)e^{(\frac{-t}{\tau})}$$

The decay rates are then found by taking the reciprocal of the decay lifetime (i.e. k = 1/τ). Finally, the decay rates are entered into the following hydration number equation:

$$q = A(k_{H2O} - k_{D2O} - B - n(0.075))$$

A and B are constants for different lanthanides (A = 1.2 and B = 0.25 ms^{-1} for Eu, and A = 5.0 and B = 0.06 ms^{-1} for Tb, respectively) and n represents the number of amide-bound oscillators for Eu complexes (n = 1 for **8**, n = 0 for **16**).

*Table S5 - Table of decay lifetimes, rates, and hydration numbers for complexes **8, 9, 16**, and **17** (20 µM, slits 10 nm, 0.1 ms delay).*

Complex	Decay lifetime τ (ms)	Decay rate k (ms^{-1})	Hydration number q
**8 H_2O**	0.64	1.57	**0.93**
**8 D_2O**	2.21	0.45	
**9 H_2O**	1.11	0.90	**0.60**
**9 D_2O**	1.39	0.72	
**16 H_2O**	0.37	2.70	**1.88**
**16 D_2O**	1.37	0.73	
**17 H_2O**	0.59	1.68	**2.23**
**17 D_2O**	0.86	1.16	

8 - ^{1}H- and ^{13}C-NMR Spectra:

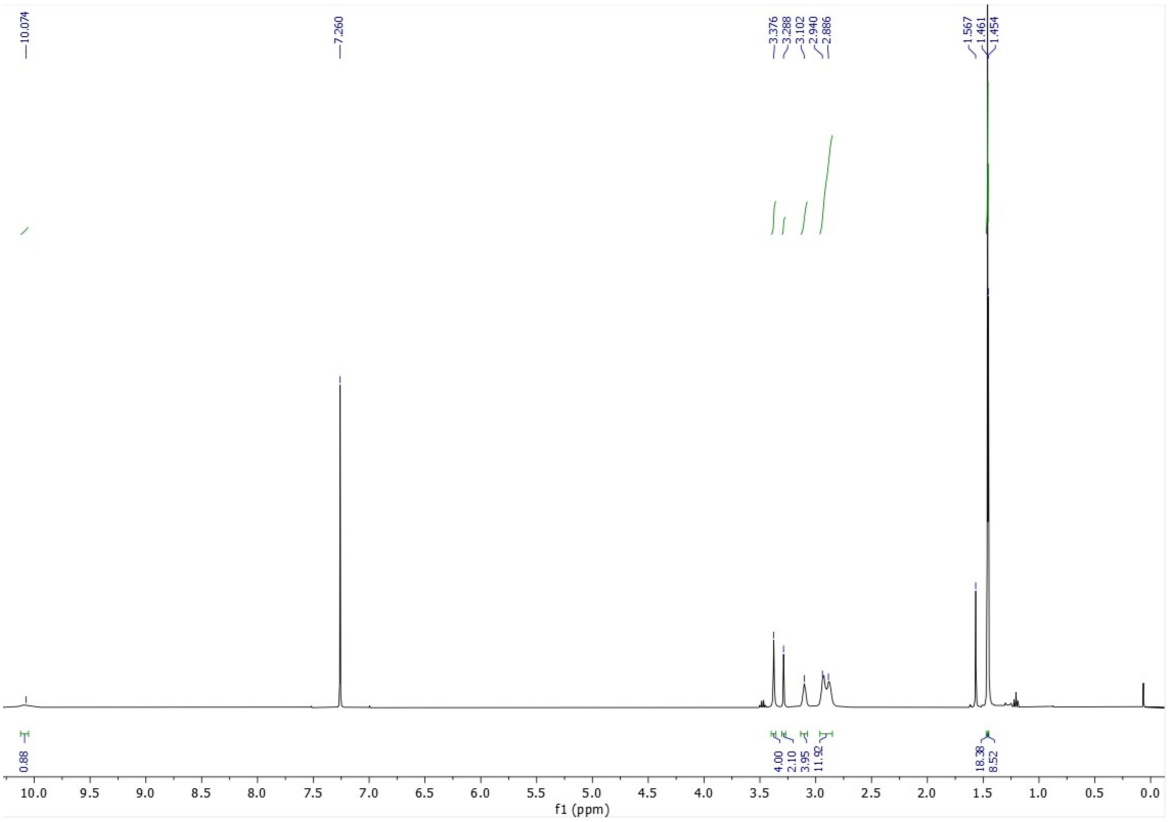

Figure S21 - ^{1}H-NMR spectrum for ***$^{t\text{-}Bu}$DO3A.HBr*** *($CDCl_3$, 298 K).*

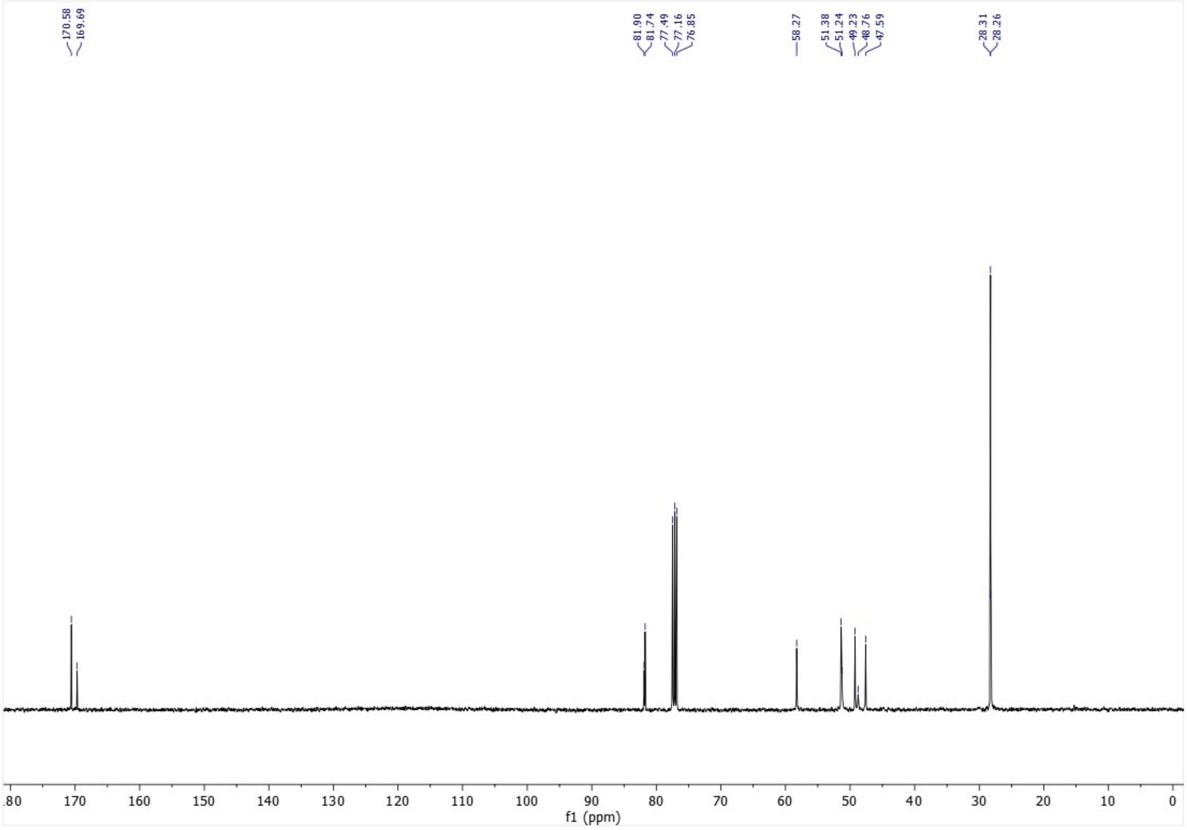

Figure S22 - ^{13}C-NMR spectrum for ***$^{t\text{-}Bu}$DO3A.HBr*** *($CDCl_3$, 298 K).*

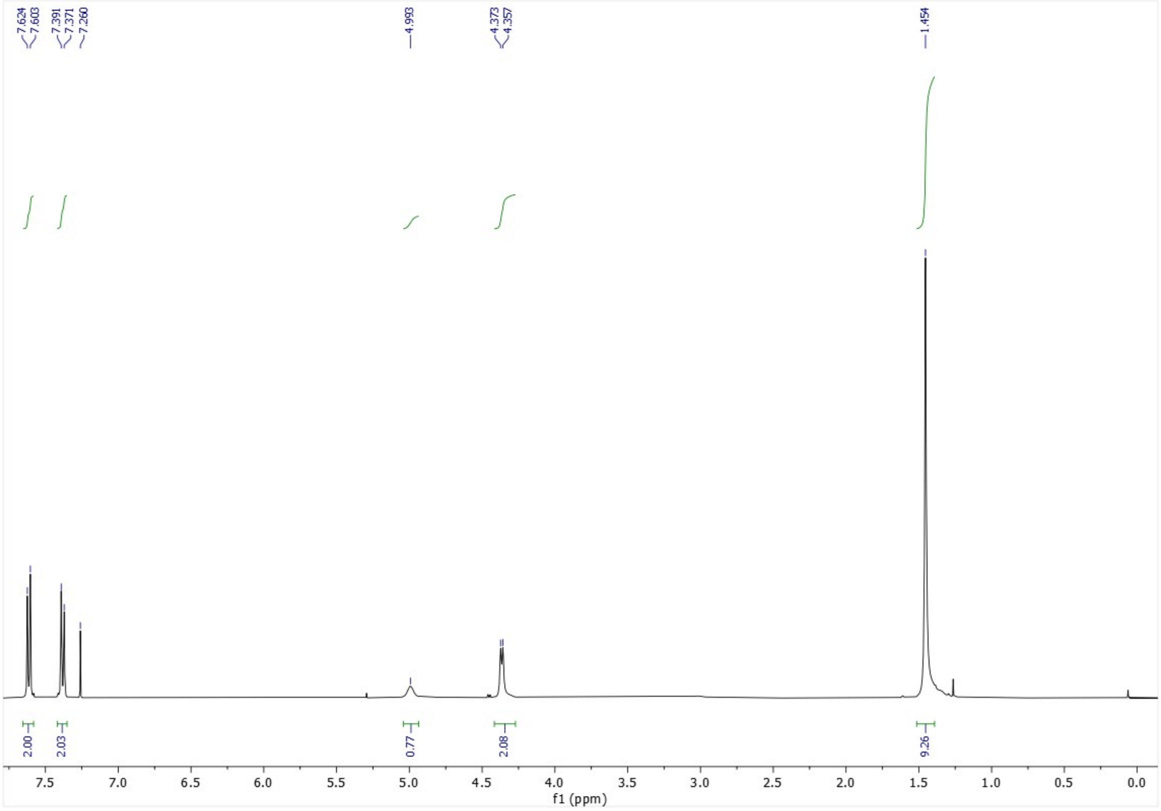

*Figure S23 - ^{1}H-NMR spectrum for **1** ($CDCl_3$, 298 K).*

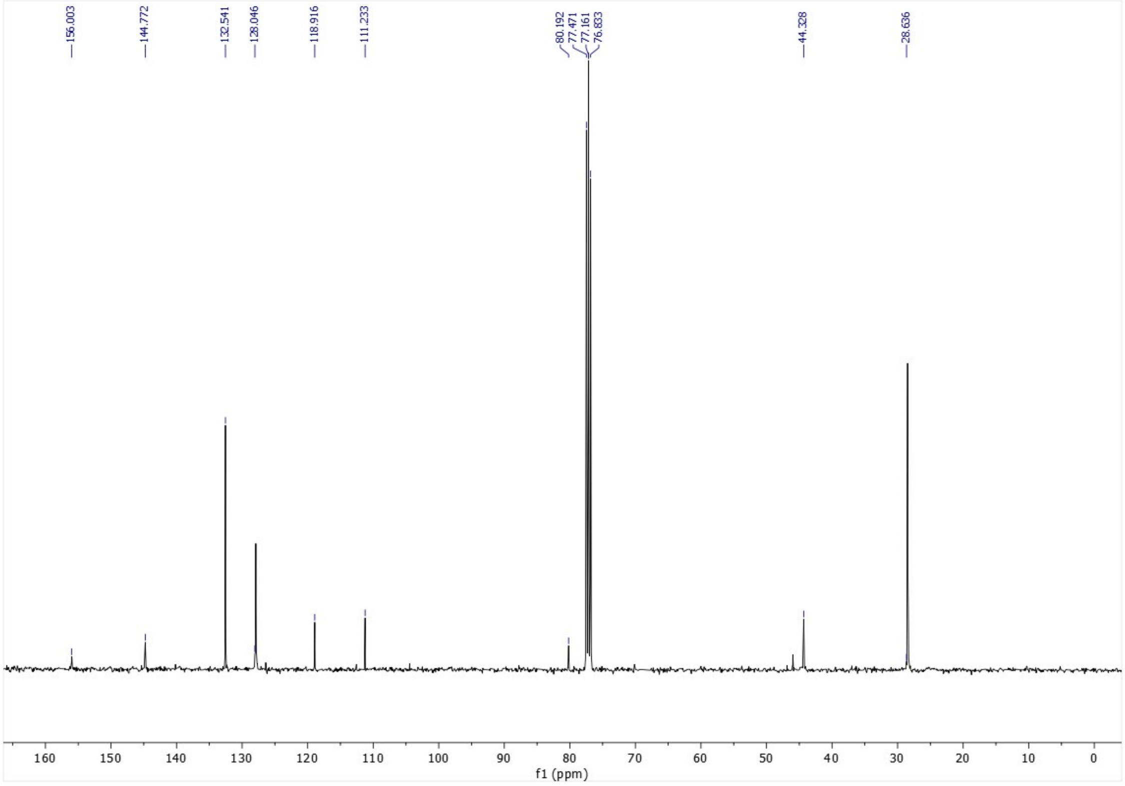

*Figure S24 - ^{13}C-NMR spectrum for **1** ($CDCl_3$, 298 K).*

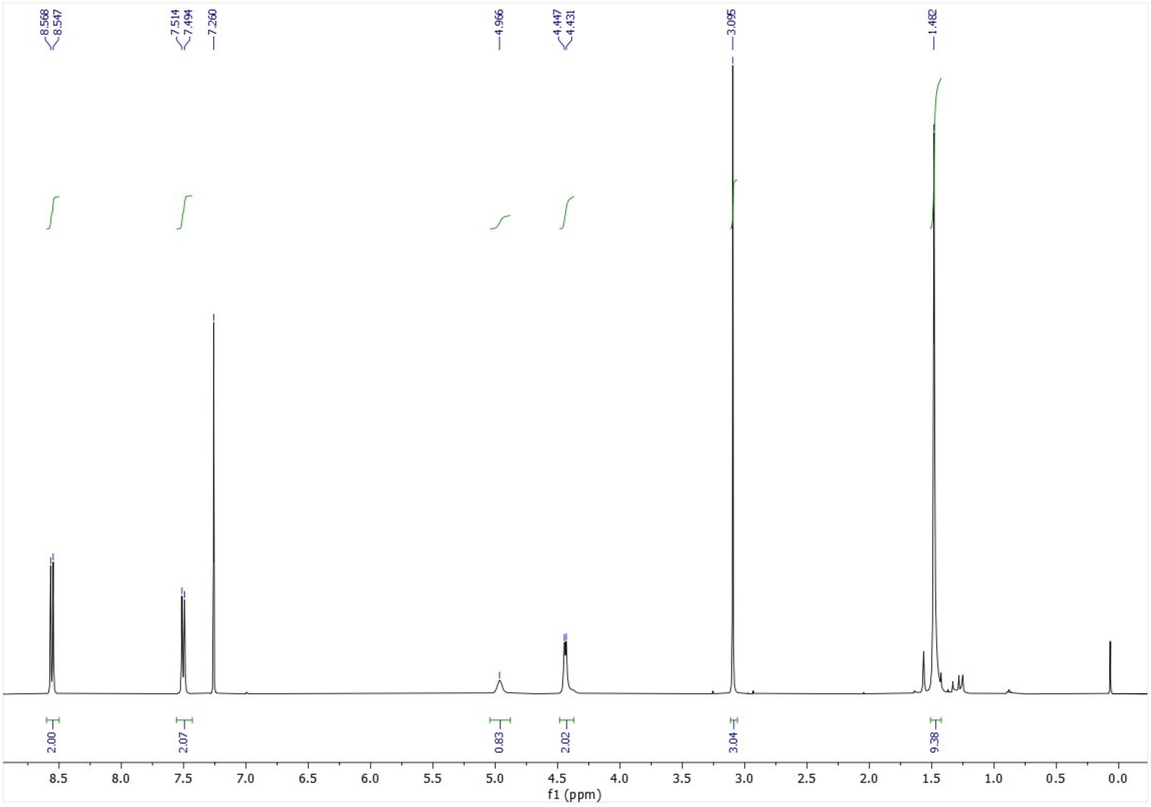

*Figure S25 - ^{1}H-NMR spectrum for **2** ($CDCl_3$, 298 K).*

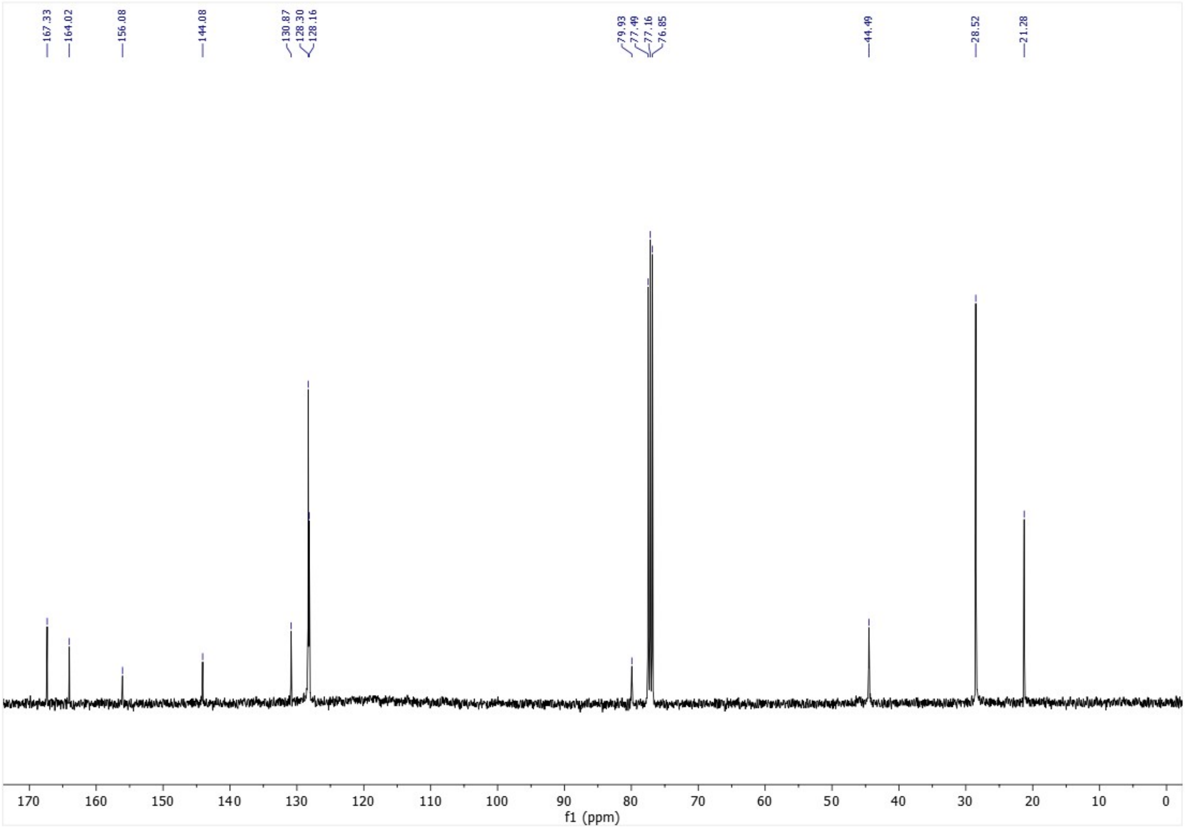

*Figure S26 - ^{13}C-NMR spectrum for **2** ($CDCl_3$, 298 K).*

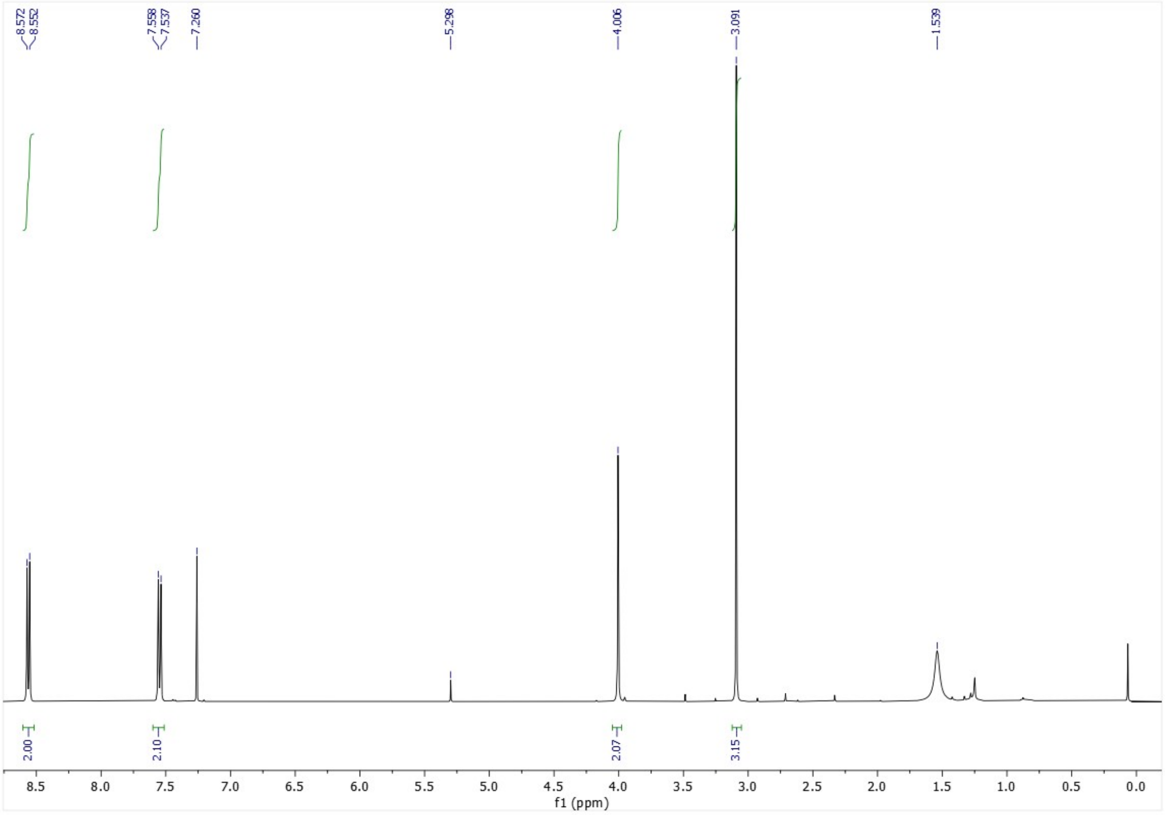

*Figure S27 - ^{1}H-NMR spectrum for **3** ($CDCl_3$, 298 K).*

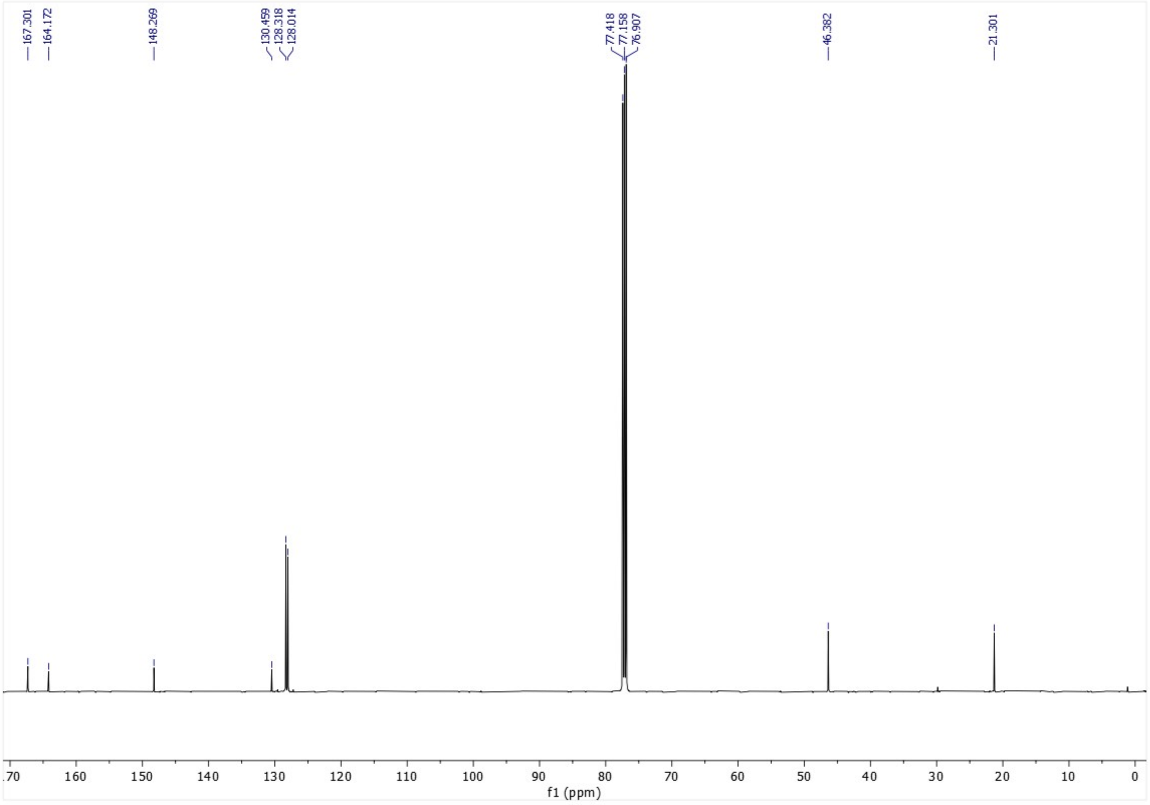

*Figure S28 - ^{13}C-NMR spectrum for **3** ($CDCl_3$, 298 K).*

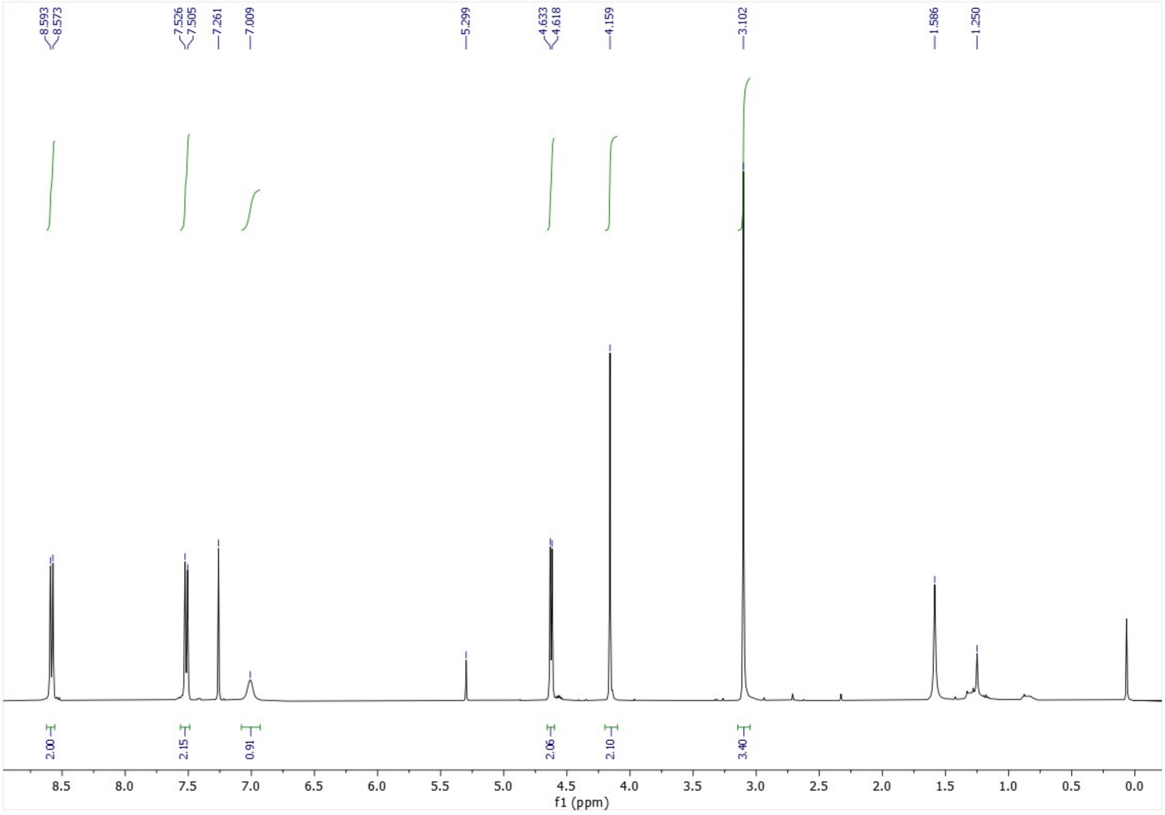

*Figure S29 - ¹H-NMR spectrum for **4** (CDCl$_3$, 298 K).*

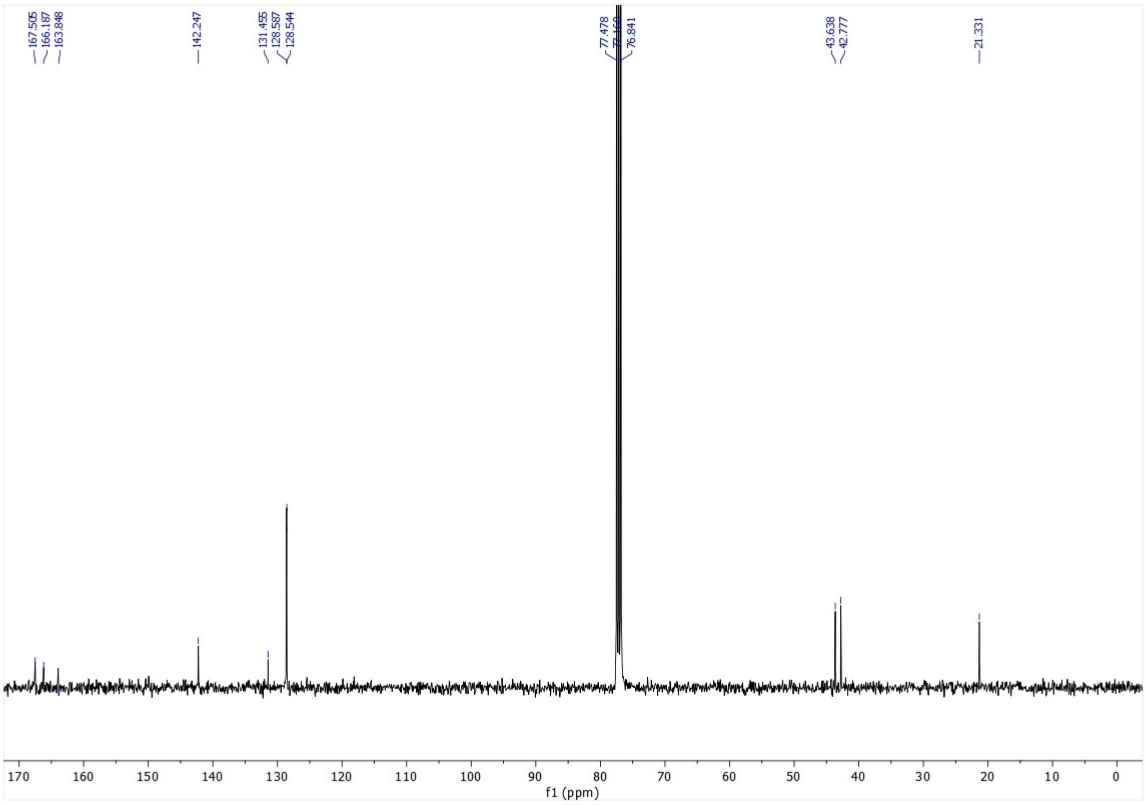

*Figure S30 - ¹³C-NMR spectrum for **4** (CDCl$_3$, 298 K).*

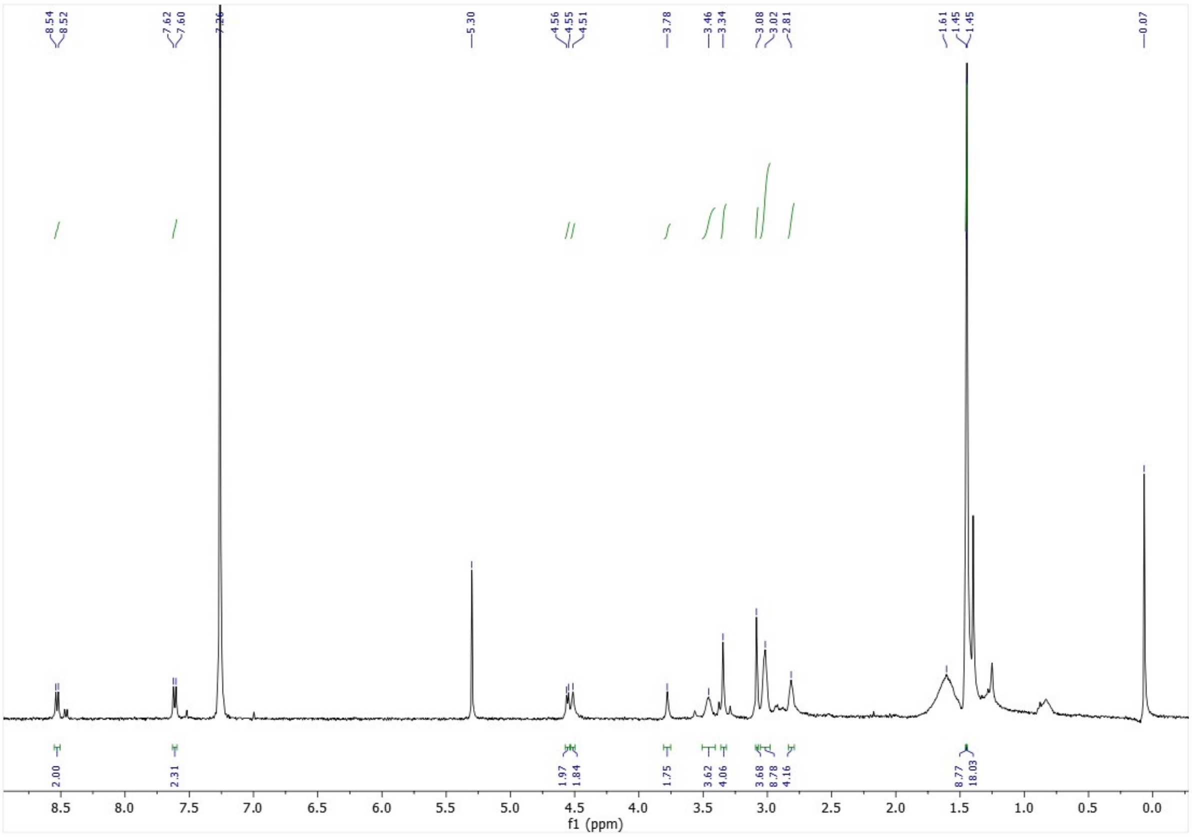

*Figure S31 - ^{1}H-NMR spectrum for **5** ($CDCl_3$, 298 K).*

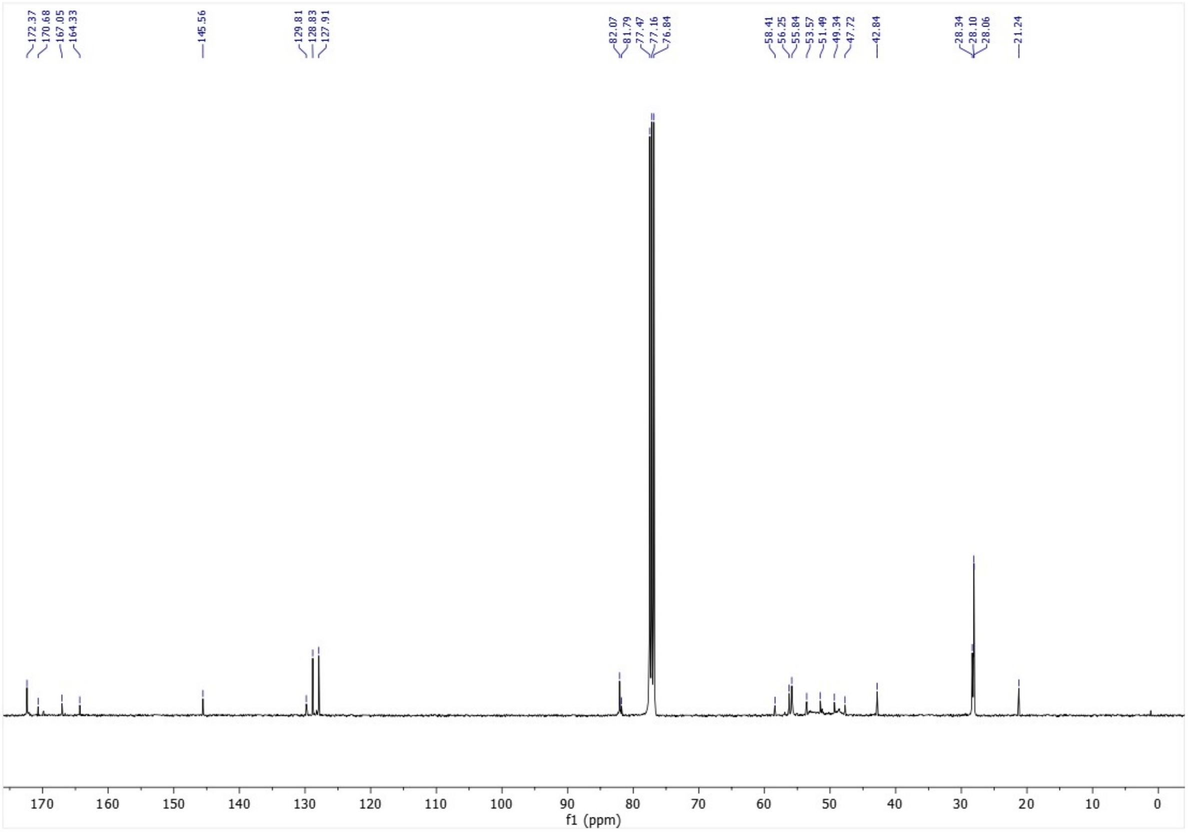

*Figure S32 - ^{13}C-NMR spectrum for **5** ($CDCl_3$, 298 K).*

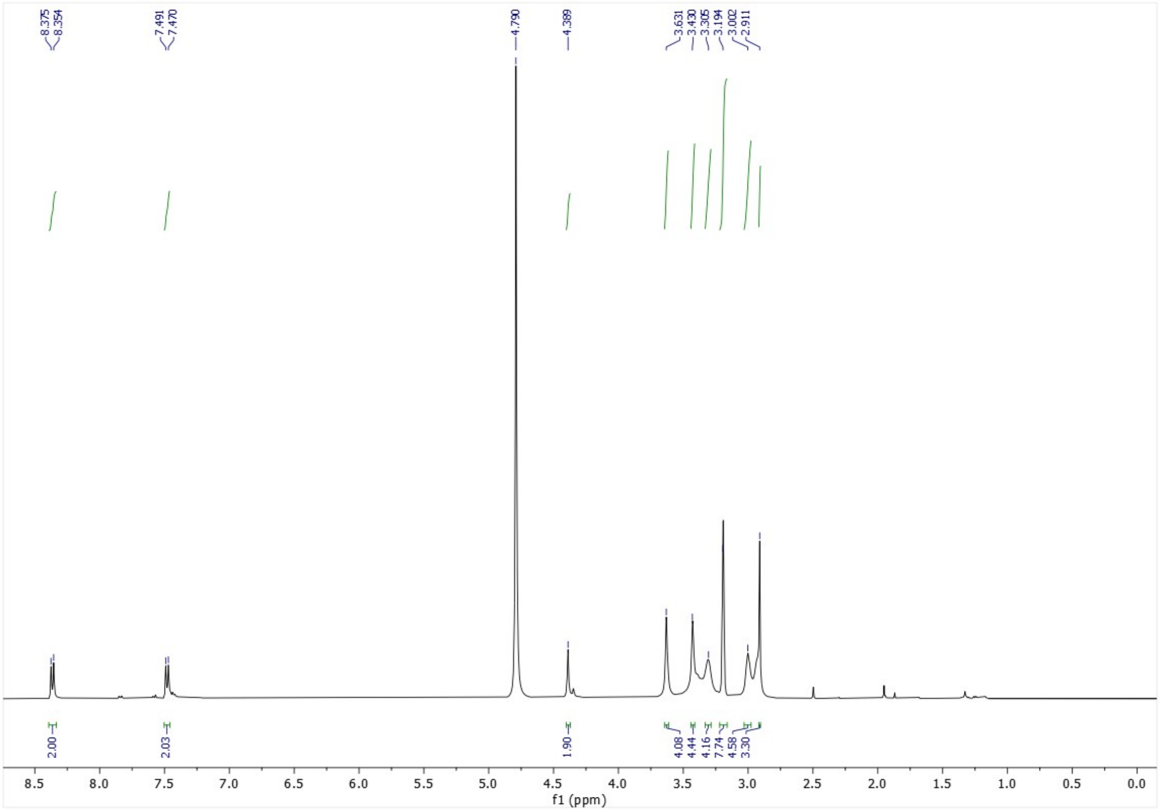

*Figure S33 - ^{1}H-NMR spectrum for **6** (D_2O, 298 K).*

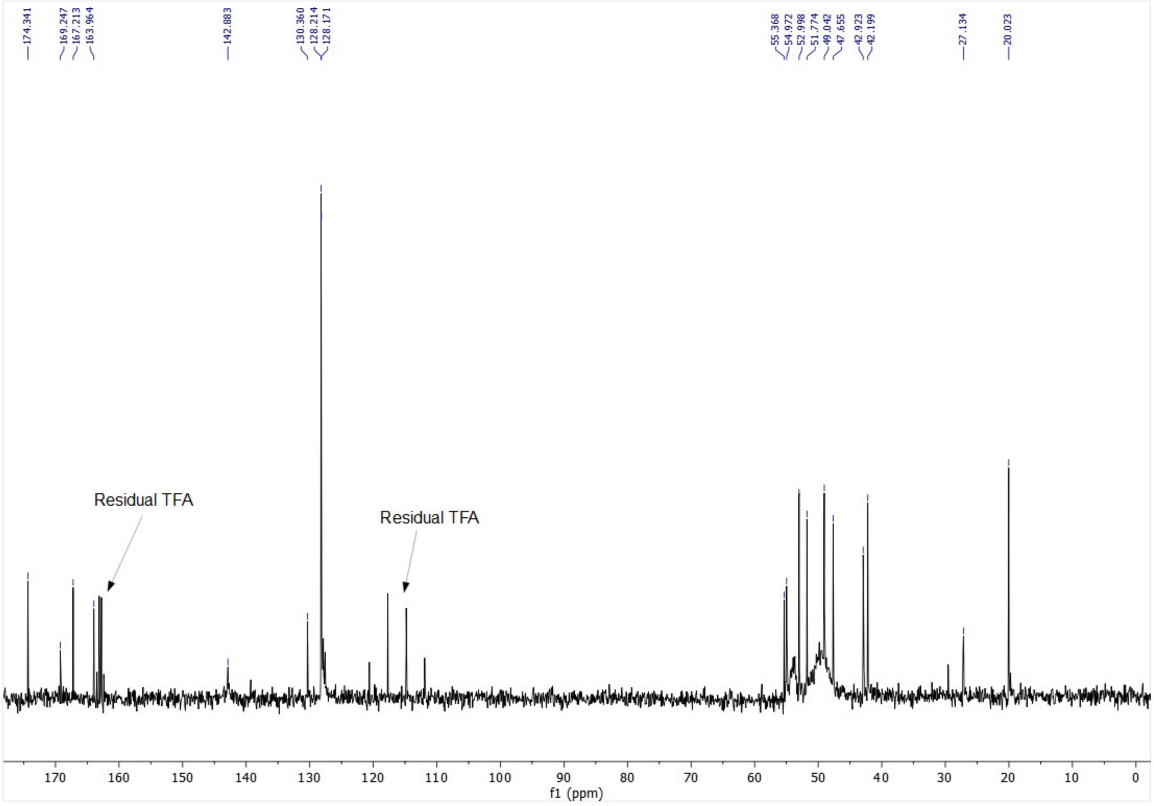

Figure S34 - ^{13}C-NMR spectrum for 6 (D_2O, 298 K).

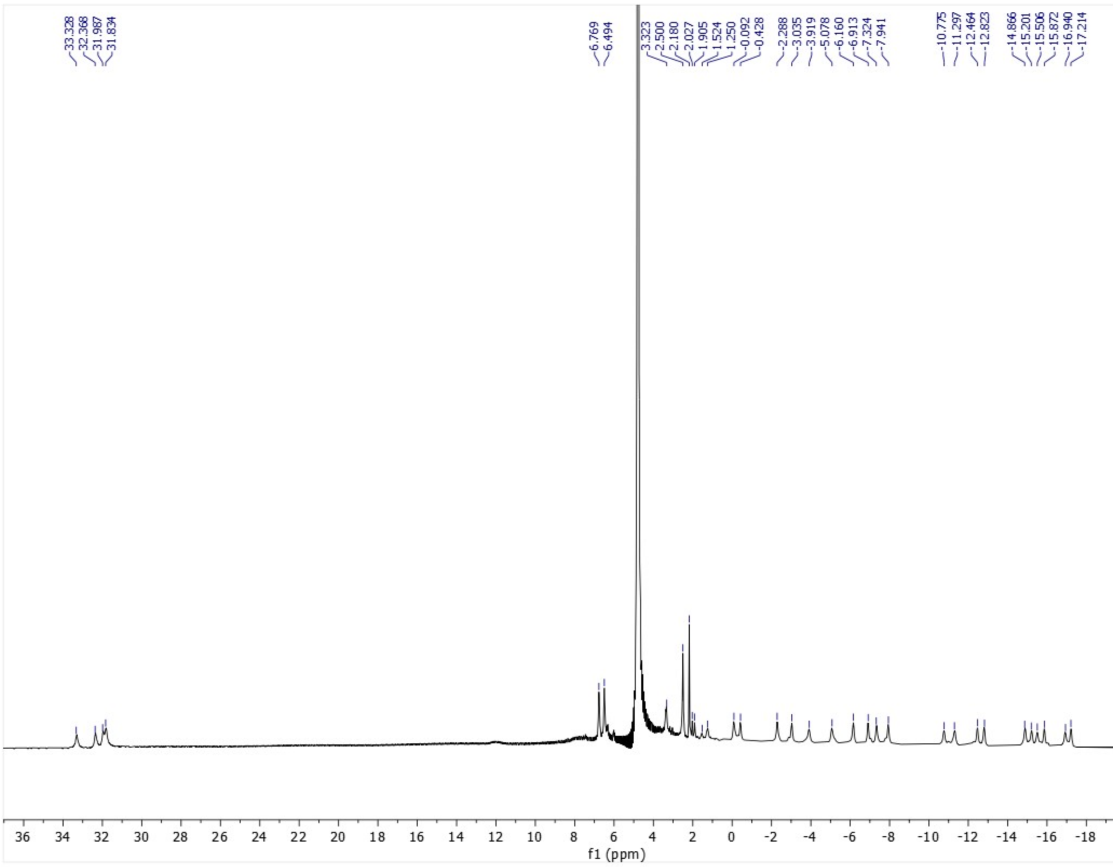

*Figure S35 - ^{1}H-NMR spectrum for **8** (D_2O, 298 K).*

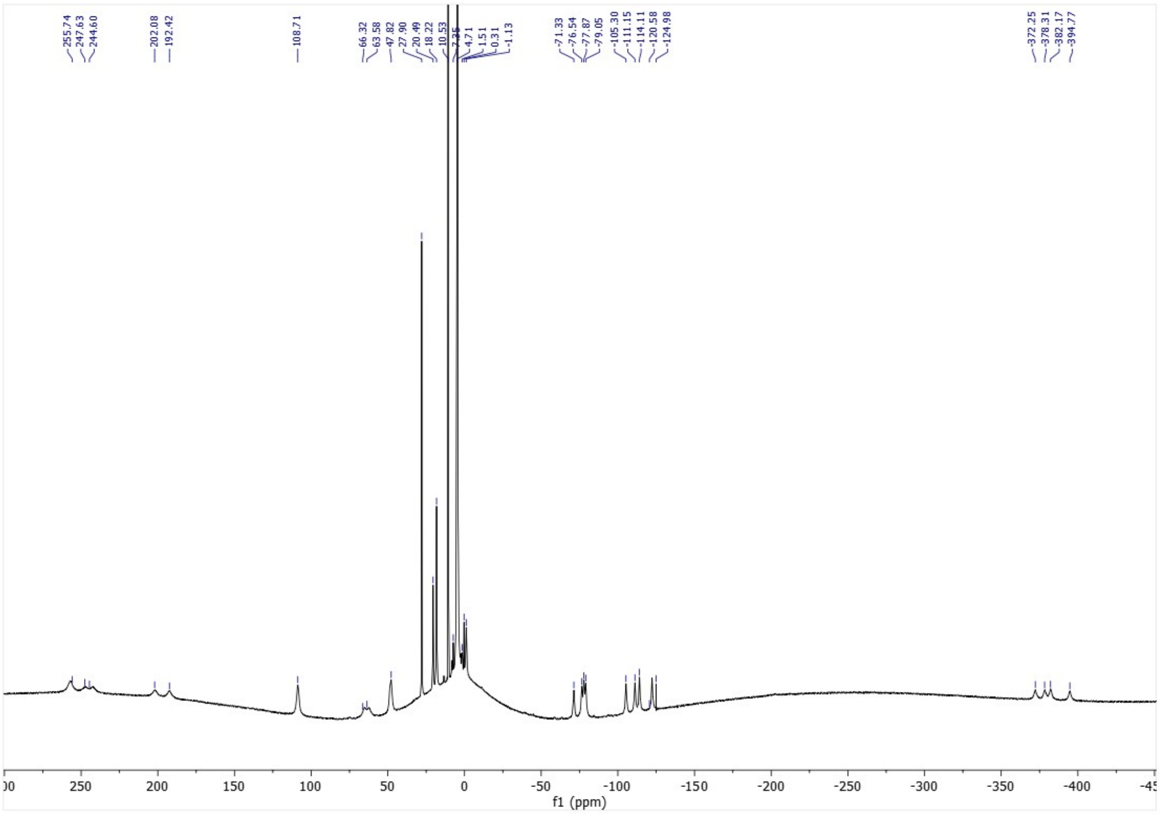

*Figure S36 - ^{1}H-NMR spectrum for **9** (D_2O, 298 K).*

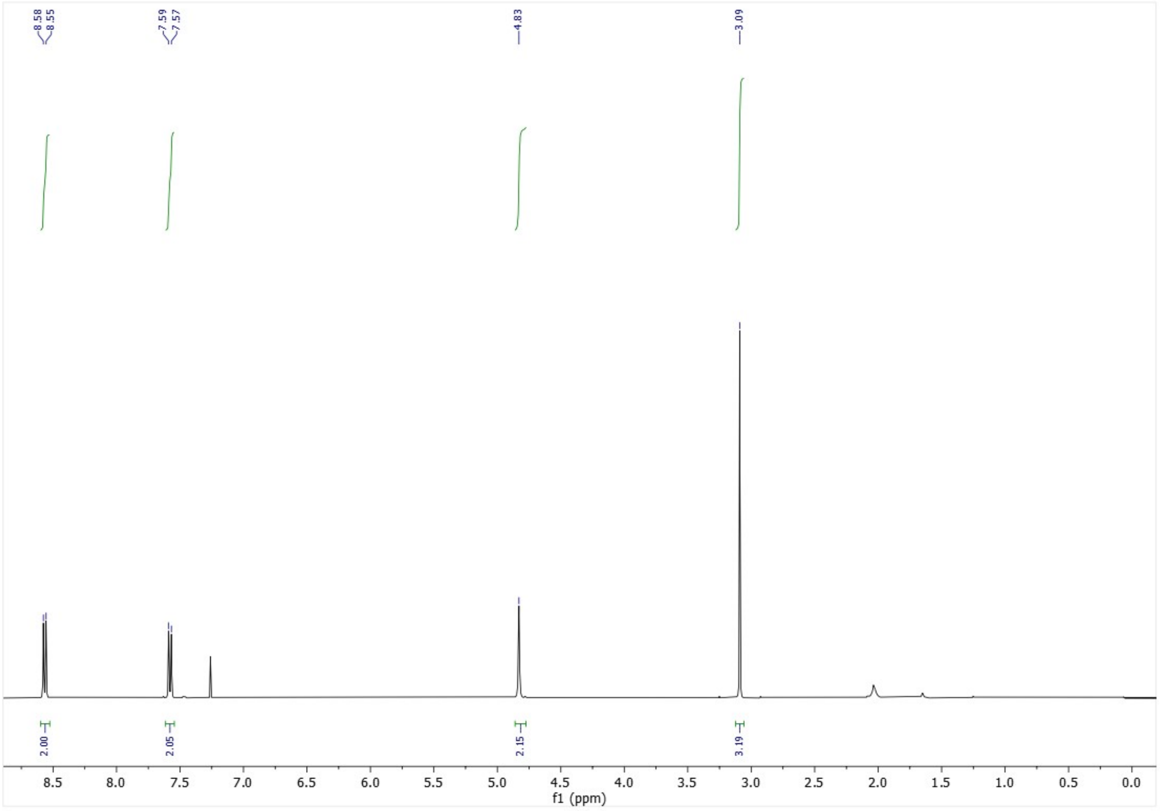

*Figure S37 - ^{1}H-NMR spectrum for **10** ($CDCl_3$, 298 K).*

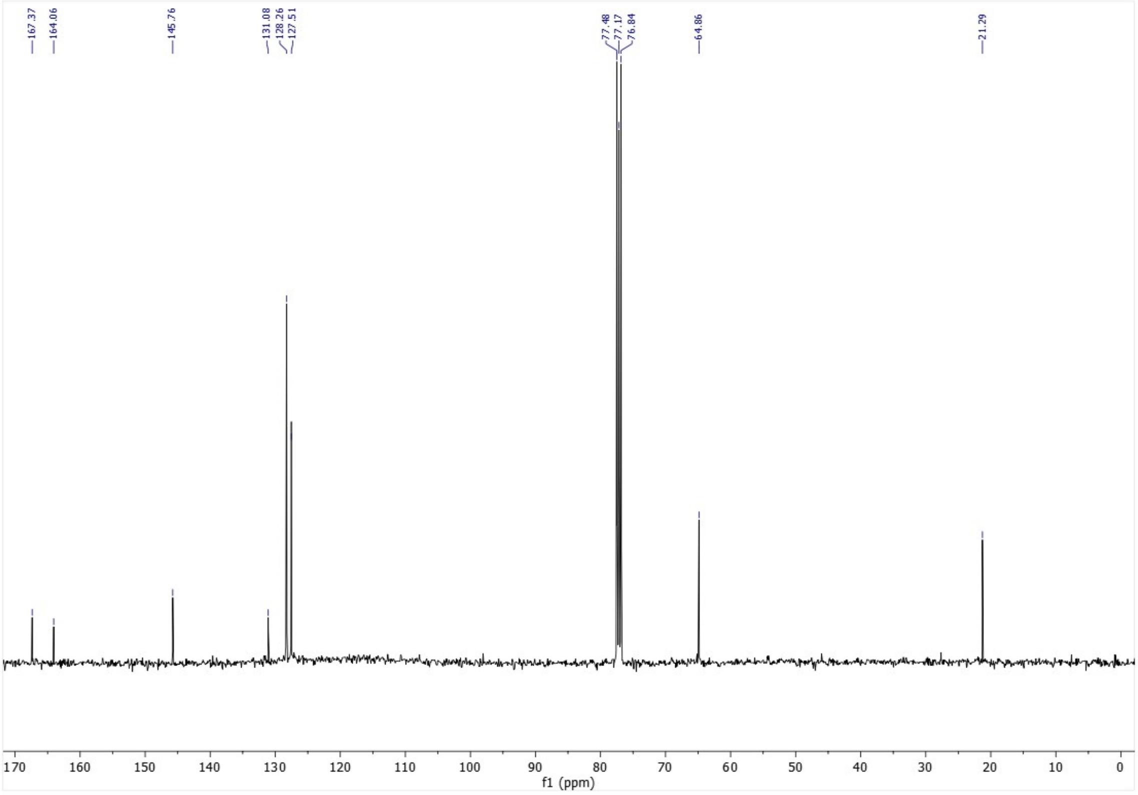

*Figure S38 - ^{13}C-NMR spectrum for **10** ($CDCl_3$, 298 K).*

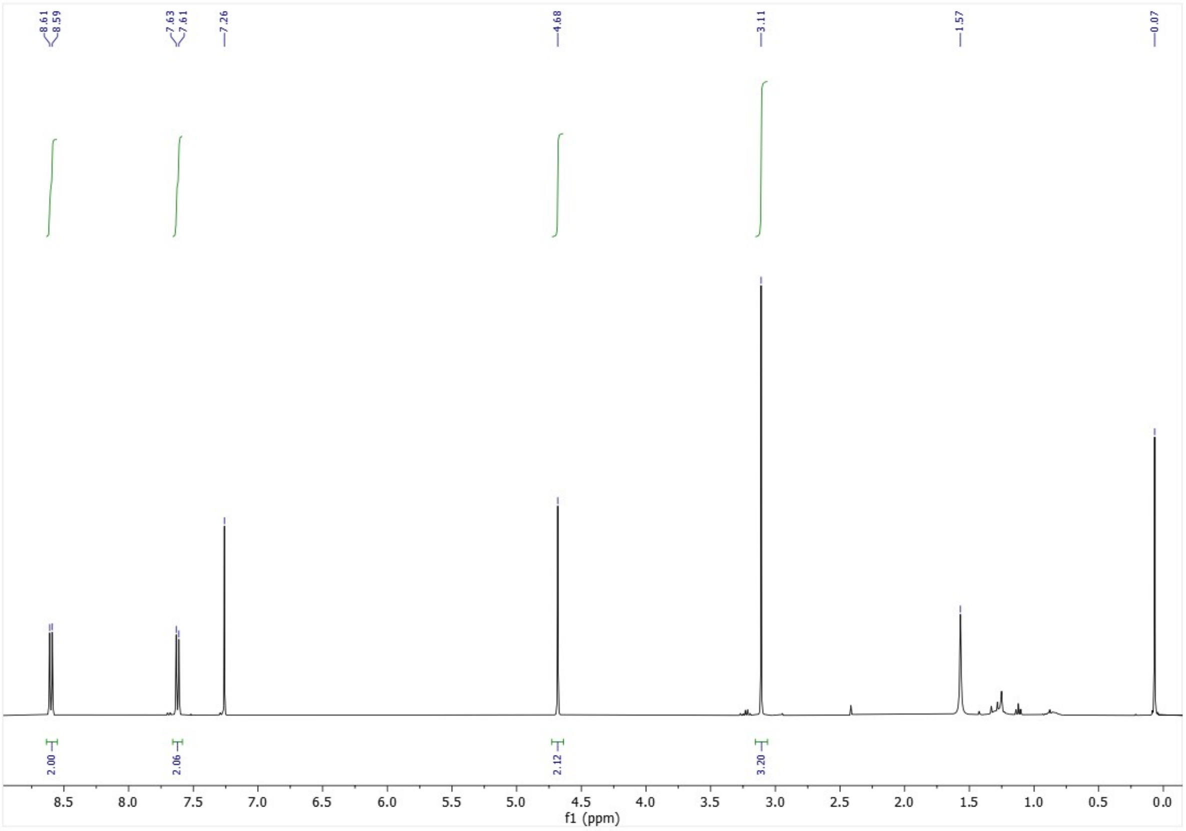

*Figure S39 - ^{1}H-NMR spectrum for **11** ($CDCl_3$, 298 K).*

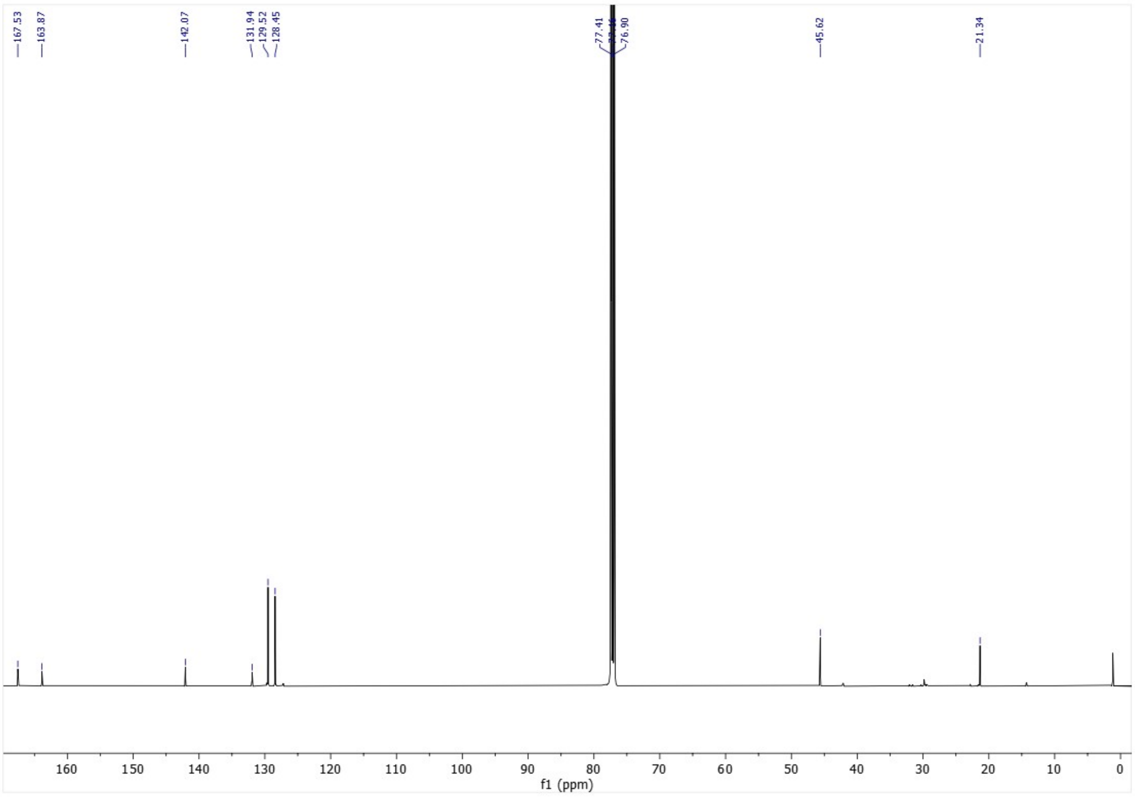

*Figure S40 - ^{13}C-NMR spectrum for **11** ($CDCl_3$, 298 K).*

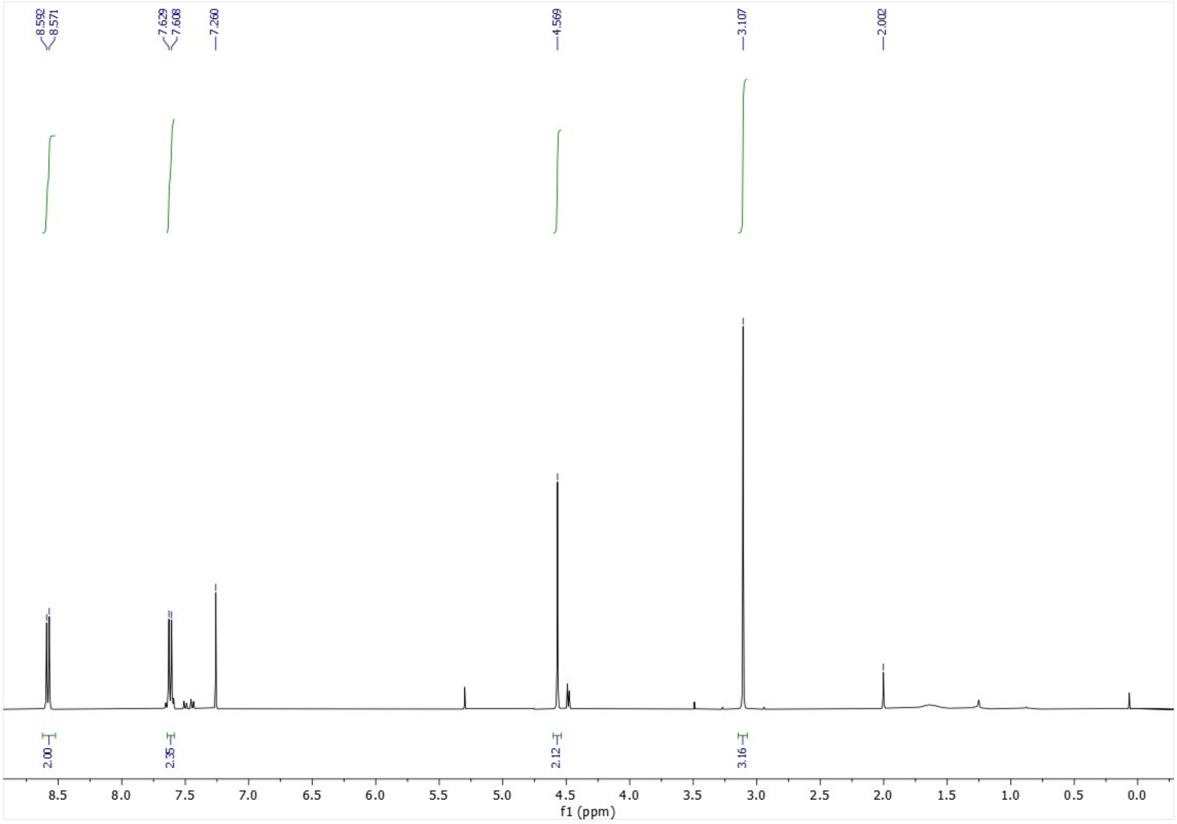

*Figure S41 - ^{1}H-NMR spectrum for **12** ($CDCl_3$, 298 K).*

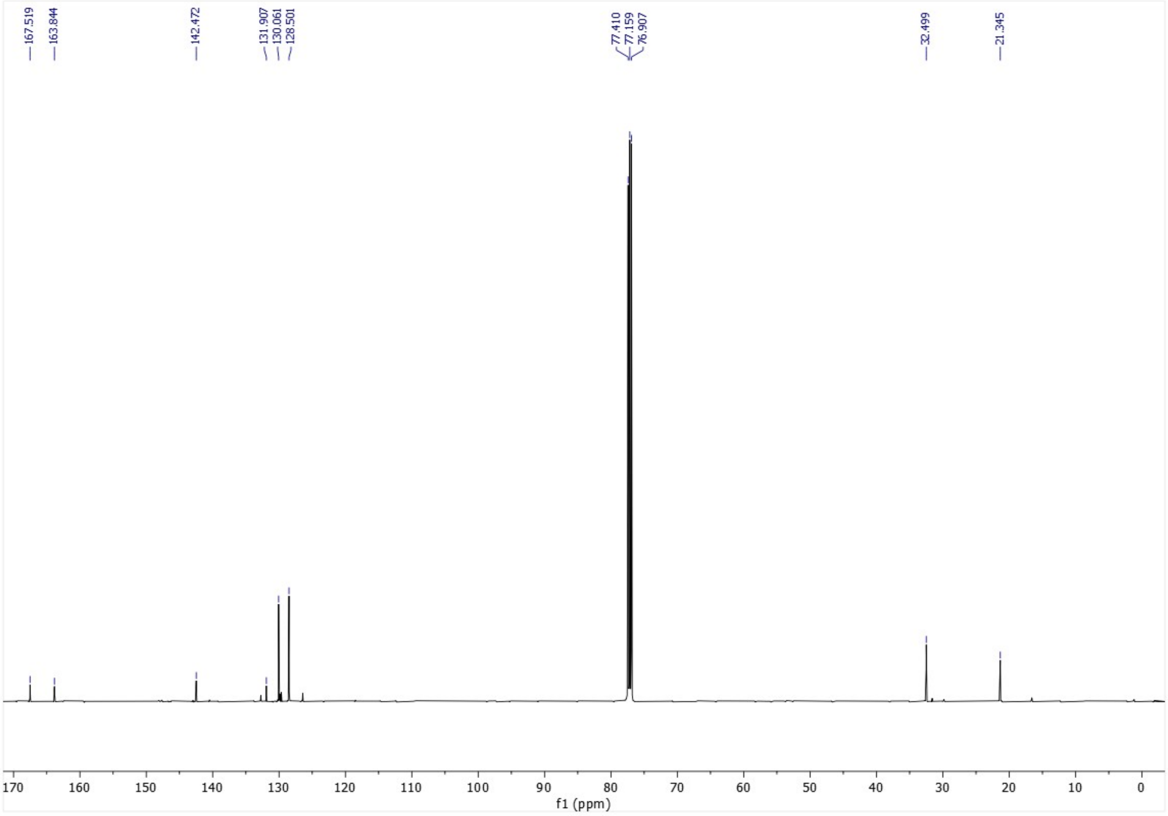

*Figure S42 - ^{13}C-NMR spectrum for **12** (CDCl$_3$, 298 K).*

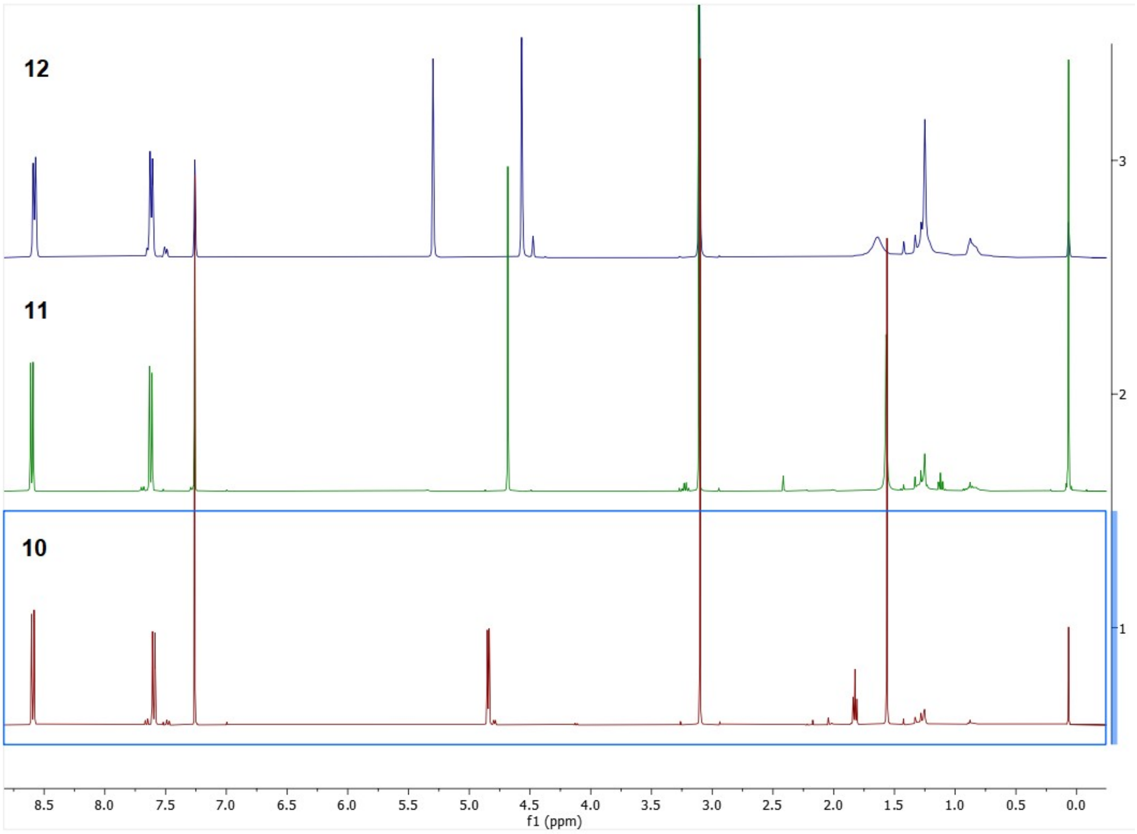

*Figure S43 - Comparison ^{1}H-NMR spectra for **10** (bottom), **11** (middle), and **12** (top) (CDCl$_3$, 298 K).*

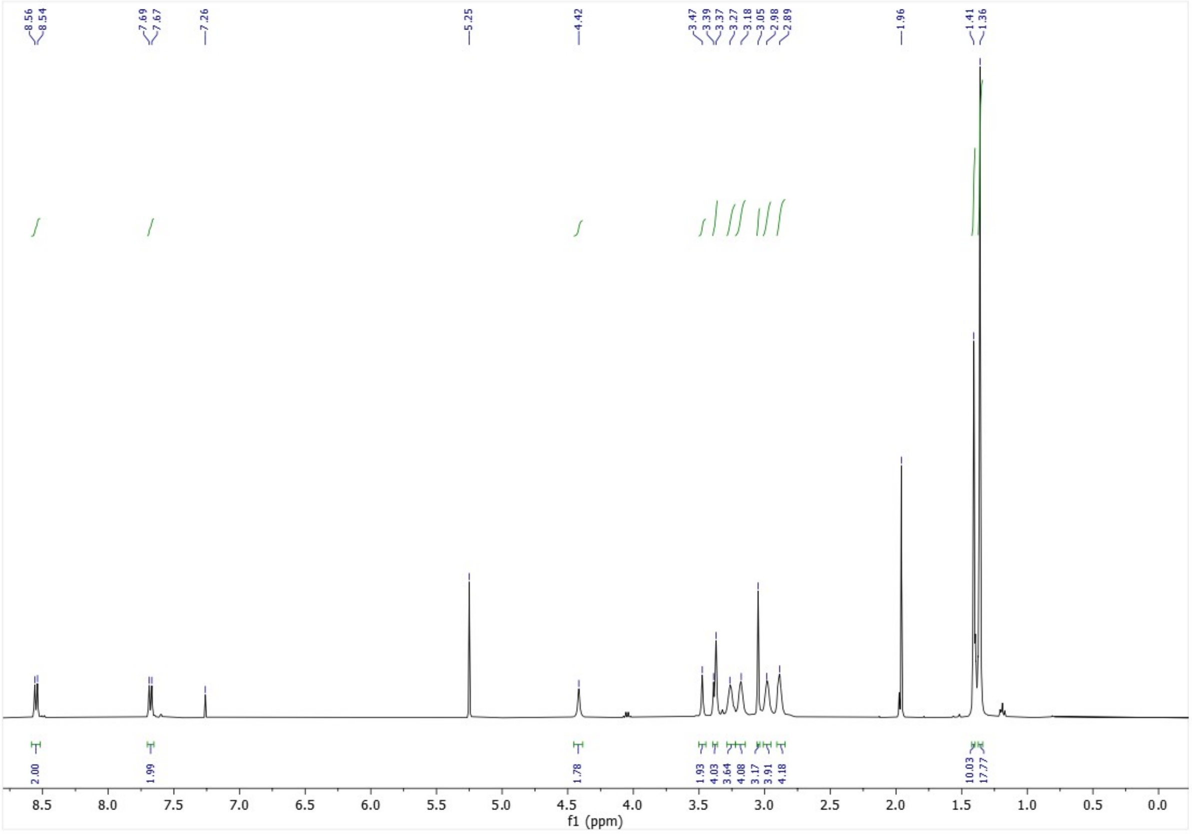

*Figure S44 - ^{1}H-NMR spectrum for **13** ($CDCl_3$, 298 K).*

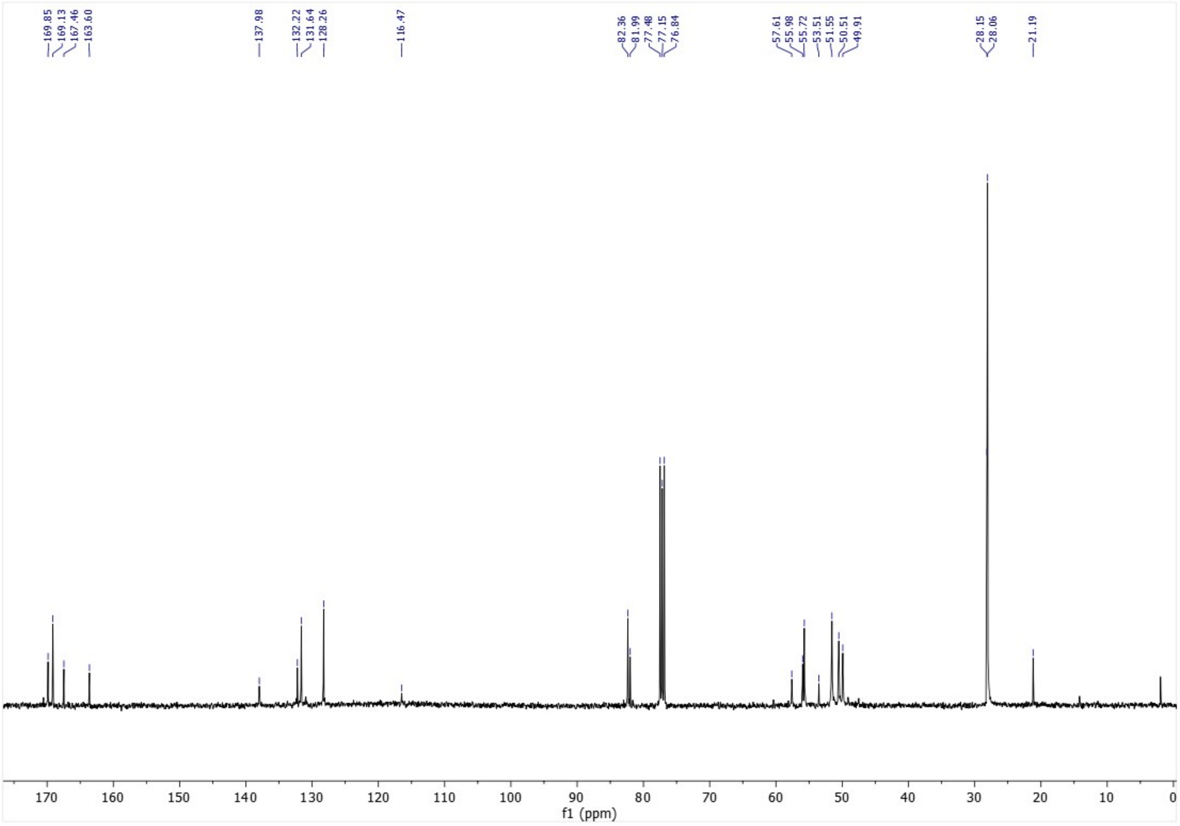

*Figure S45 - ^{13}C-NMR spectrum for **13** ($CDCl_3$, 298 K).*

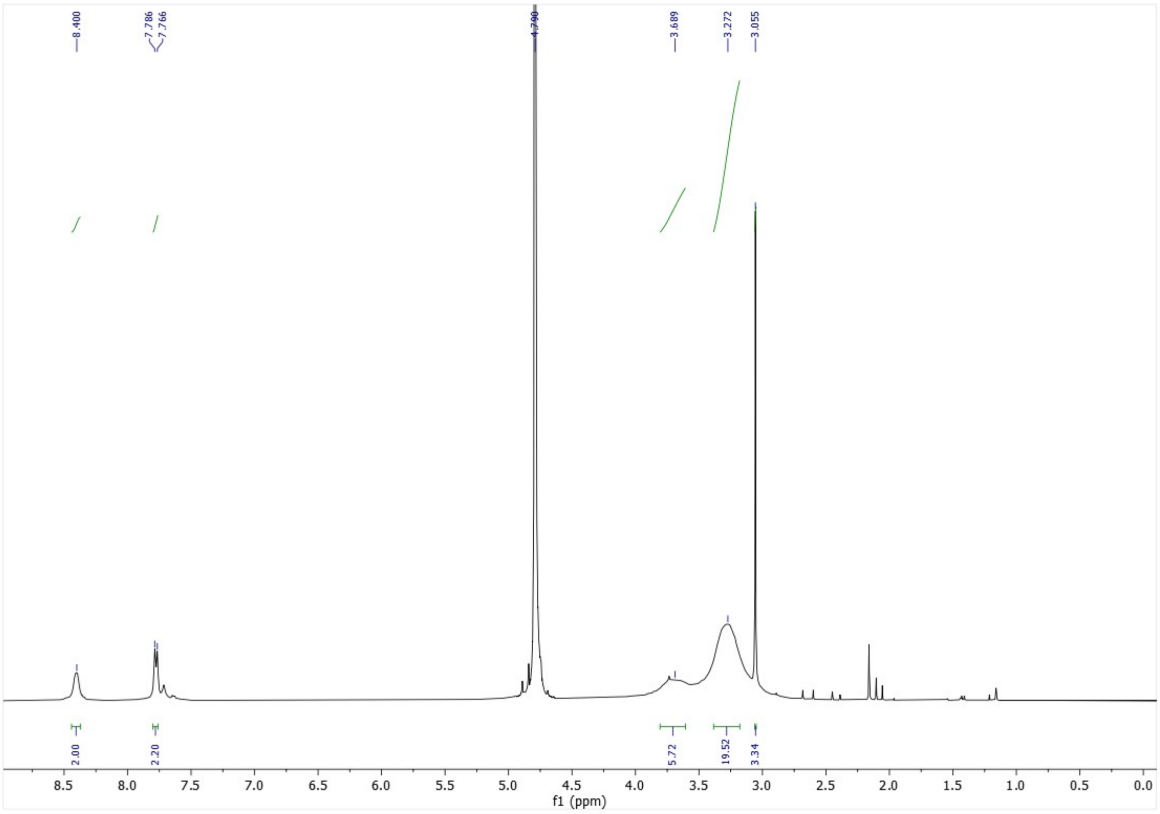

*Figure S46 - ^{1}H-NMR spectrum for **14** (D_2O, 298 K).*

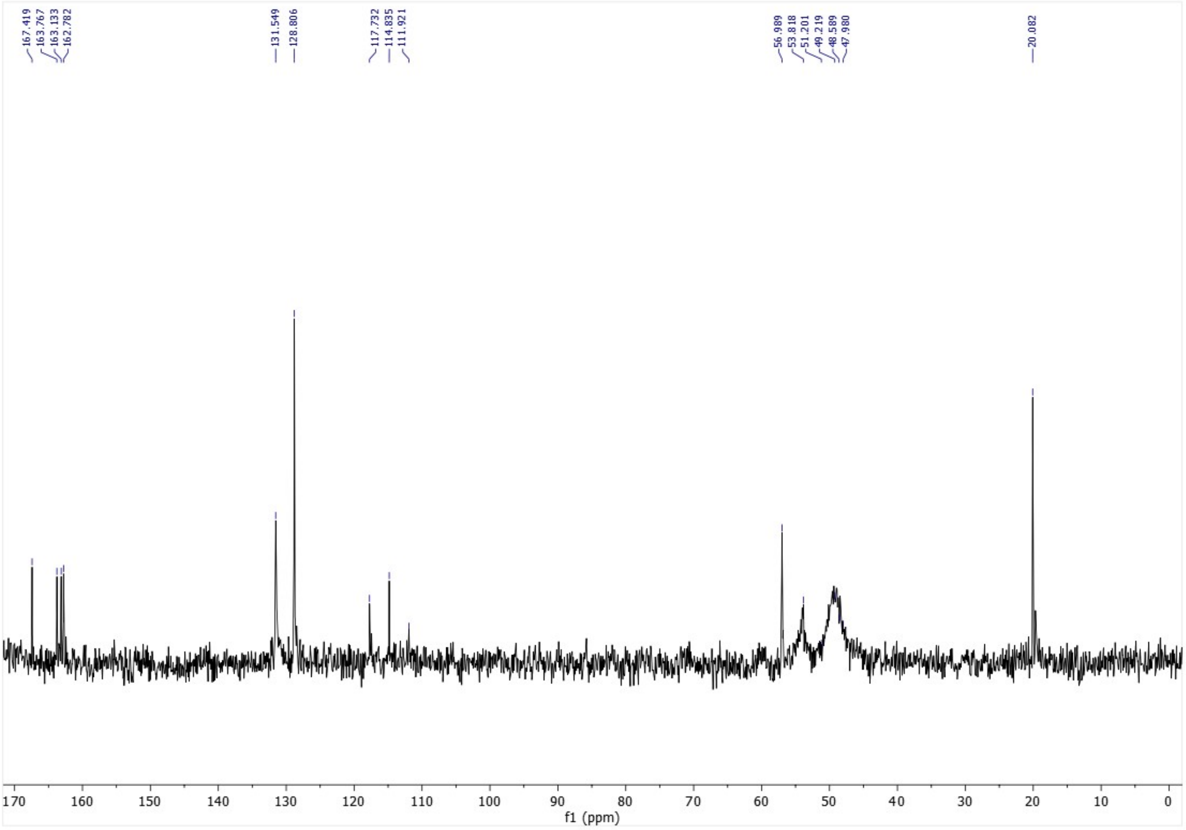

*Figure S47 - ^{13}C-NMR spectrum for **14** (D_2O, 298 K).*

9 - Mass Spectra and LCMS Traces:

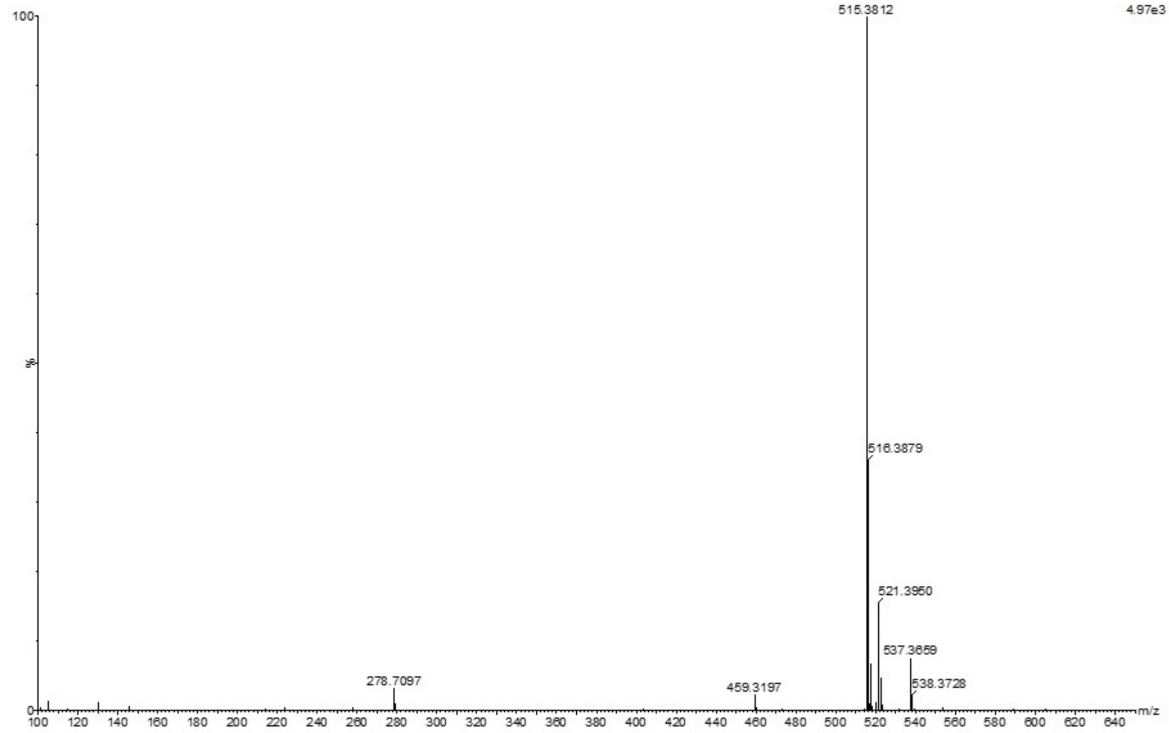

Mass	Calc. Mass	mDa	PPM	DBE	i-FIT	i-FIT (Norm)	Formula
515.3812	515.3809	0.3	0.6	3.5	105.4	0.0	C26 H51 N4 O6

Figure S48 - ES^{+} MS of ***$^{t\text{-}Bu}$DO3A.HBr.***

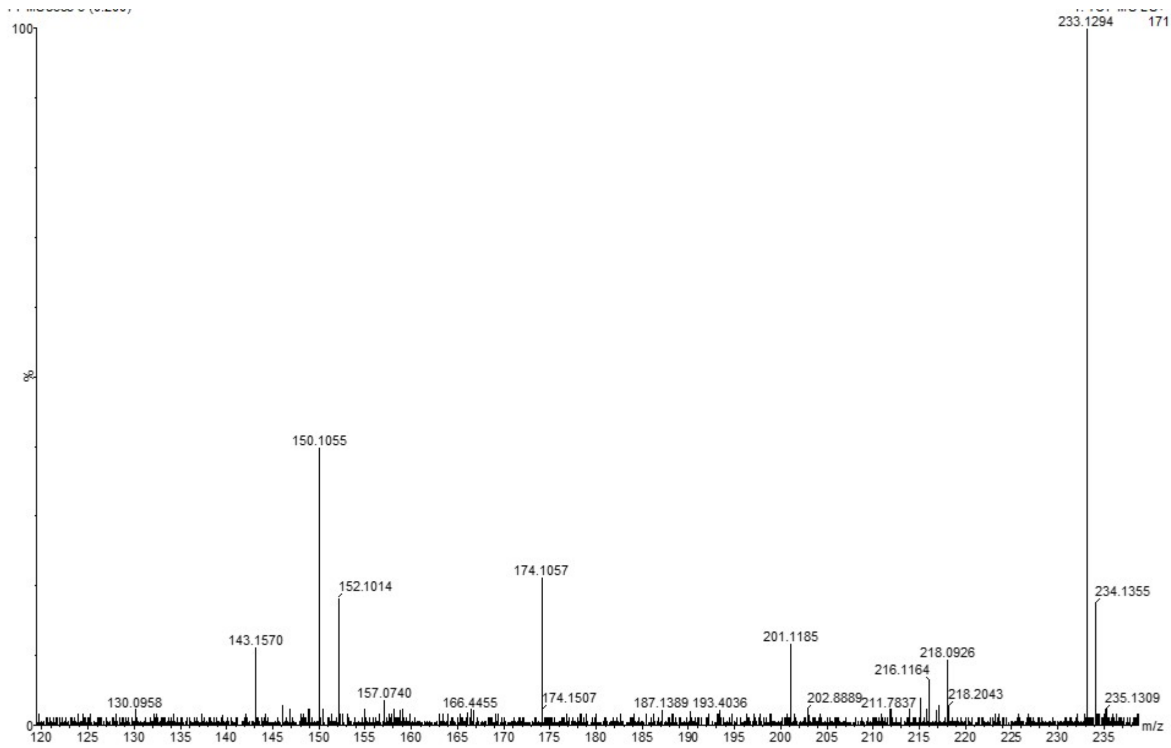

Mass	Calc. Mass	mDa	PPM	DBE	i-FIT	i-FIT (Norm)	Formula
233.1294	233.1290	0.4	1.7	6.5	66.8	0.0	C13 H17 N2 O2

Figure S49 - ES^{+} MS of ***1.***

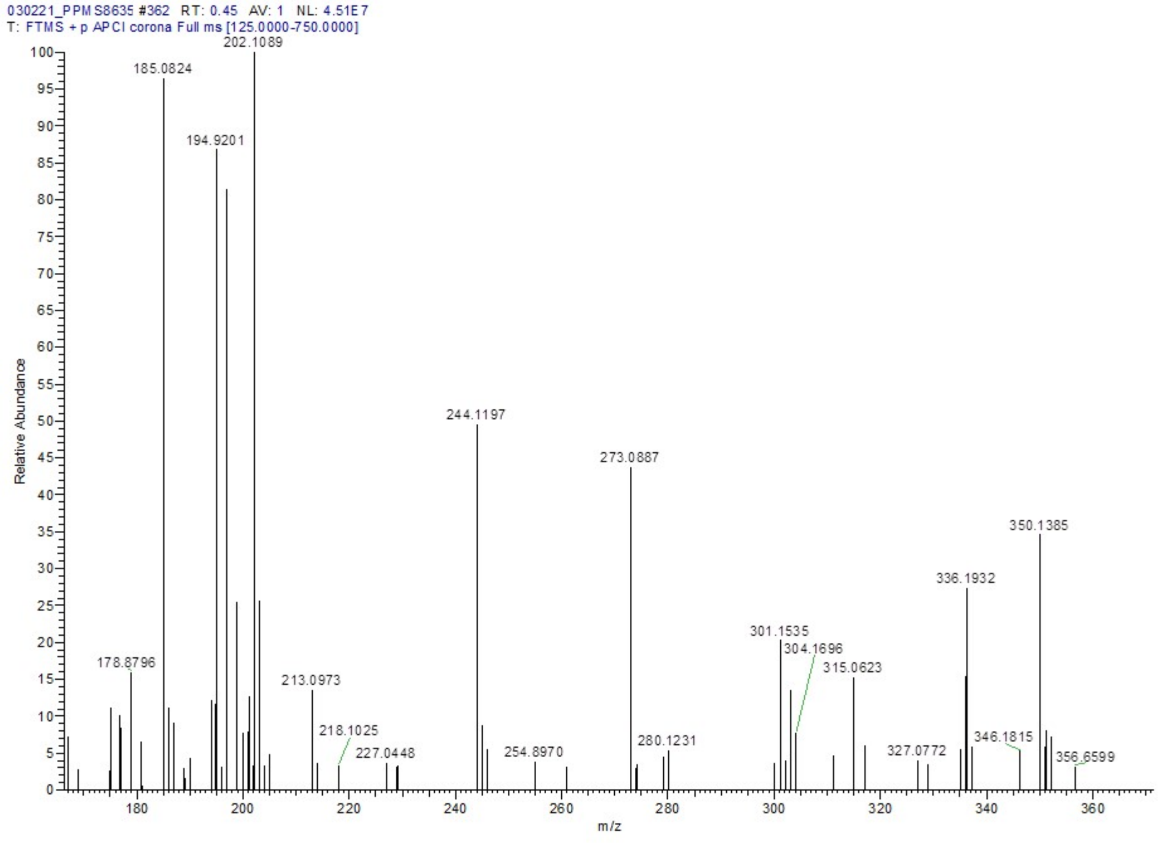

THEORETICAL MASS FOR C15H19NO2 .+ = 301.1533. FOUND MASS = 301.1535.

*Figure S50 - APCI MS for **2**.*

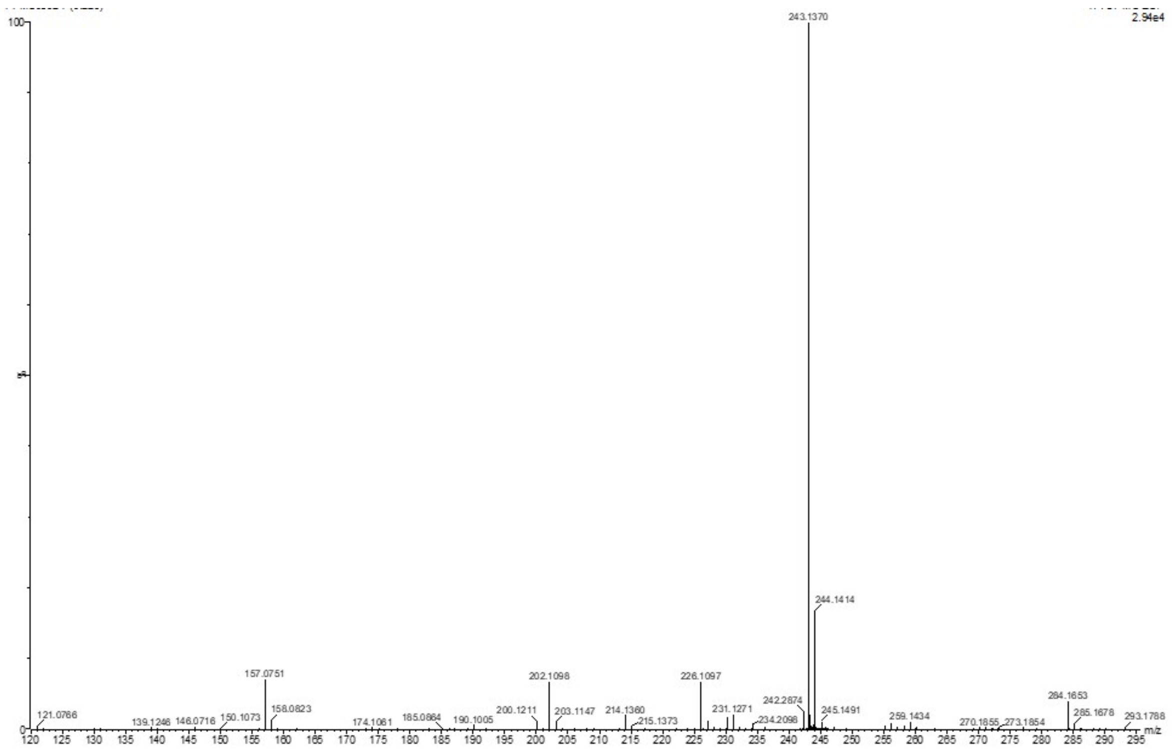

Mass	Calc. Mass	mDa	PPM	DBE	i-FIT	Formula
202.1098	202.1093	0.5	2.5	7.5	29.2	C10 H12 N5

*Figure S51 - ES$^+$ MS for **3**.*

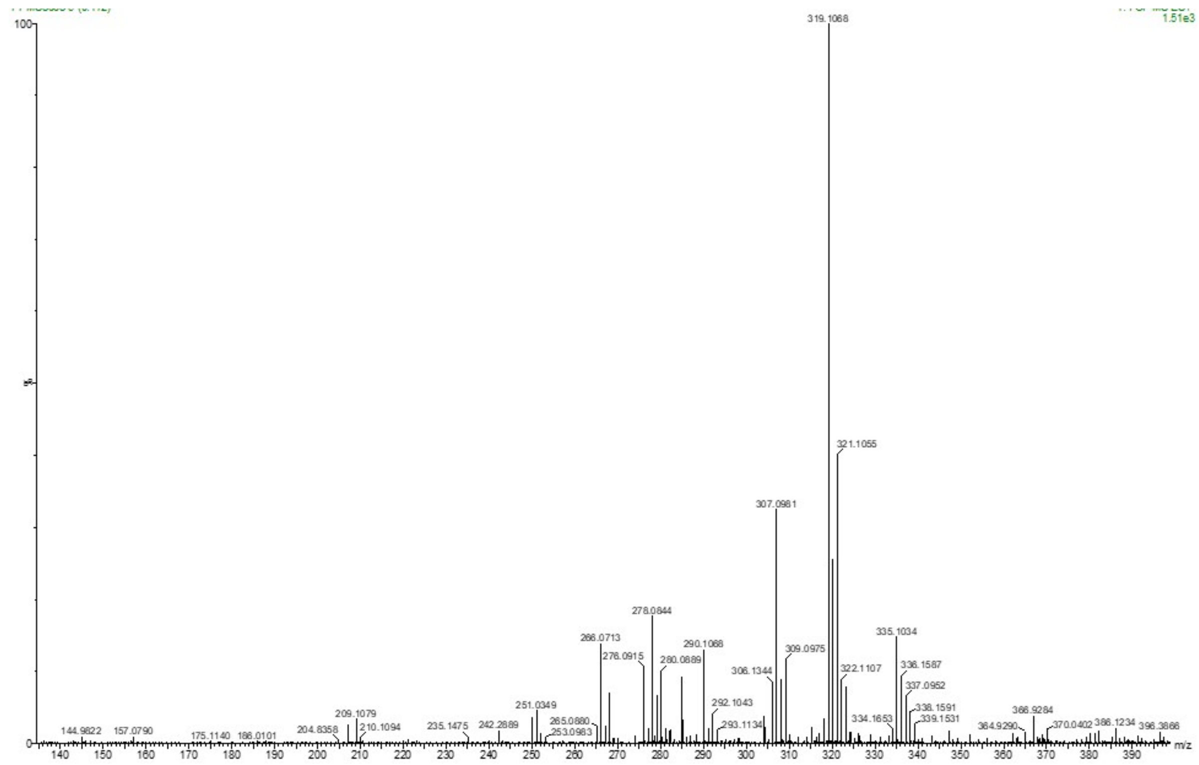

Mass	Calc. Mass	mDa	PPM	DBE	i-FIT	Formula
319.1068	319.1074	-0.6	-1.9	9.5	18.4	C14 H16 N6 O Cl

*Figure S52 - ES⁺ MS for **4**.*

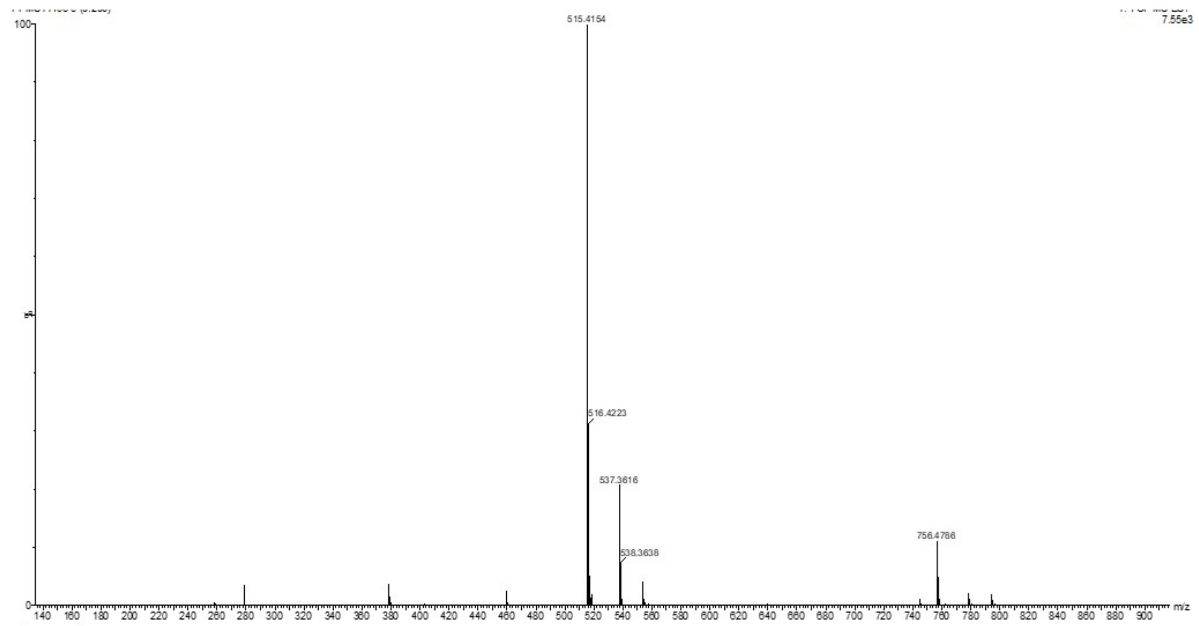

Elemental Composition Report

Mass	Calc. Mass	mDa	PPM	DBE	i-FIT	Formula
756.4786	756.4772	1.4	1.9	12.5	0.8	C38 H62 N9 O7

*Figure S53 - ES⁺ MS for **5**.*

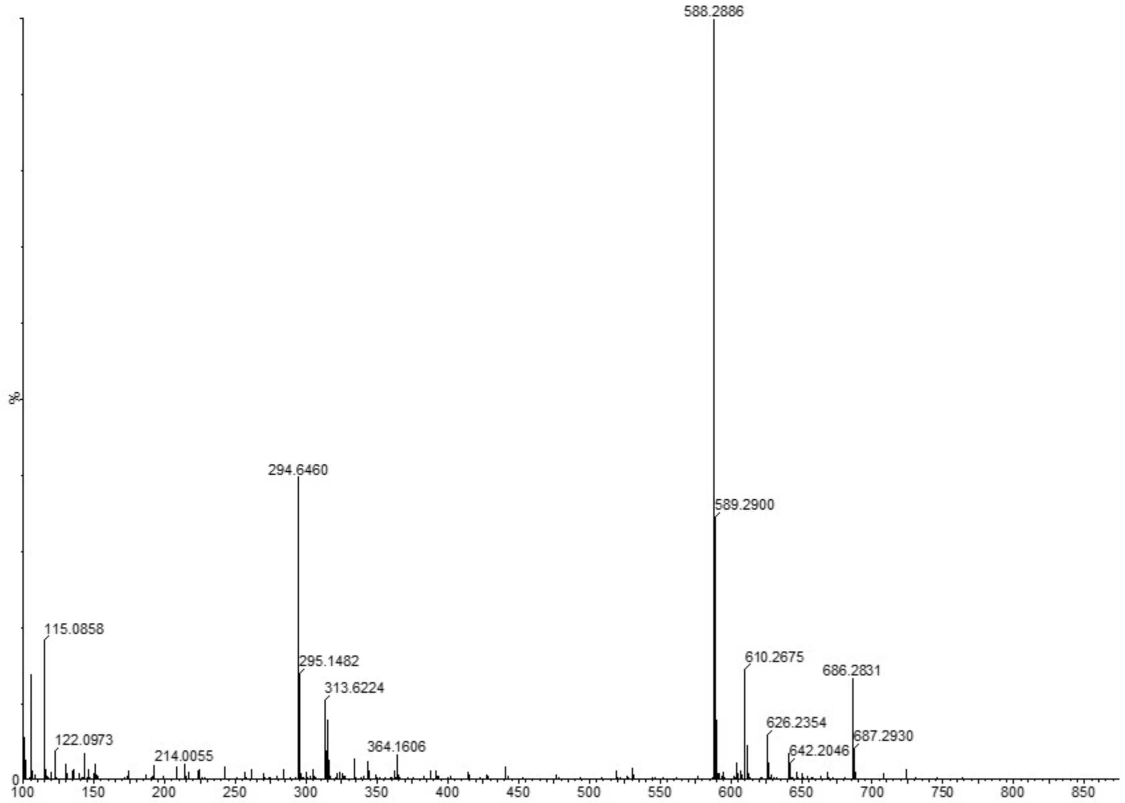

Mass	Calc. Mass	mDa	PPM	DBE	i-FIT	i-FIT (Norm)	Formula
588.2886	588.2894	-0.8	-1.4	12.5	97.1	0.0	C26 H38 N9 O7

*Figure S54 - ES⁺ MS of **6**.*

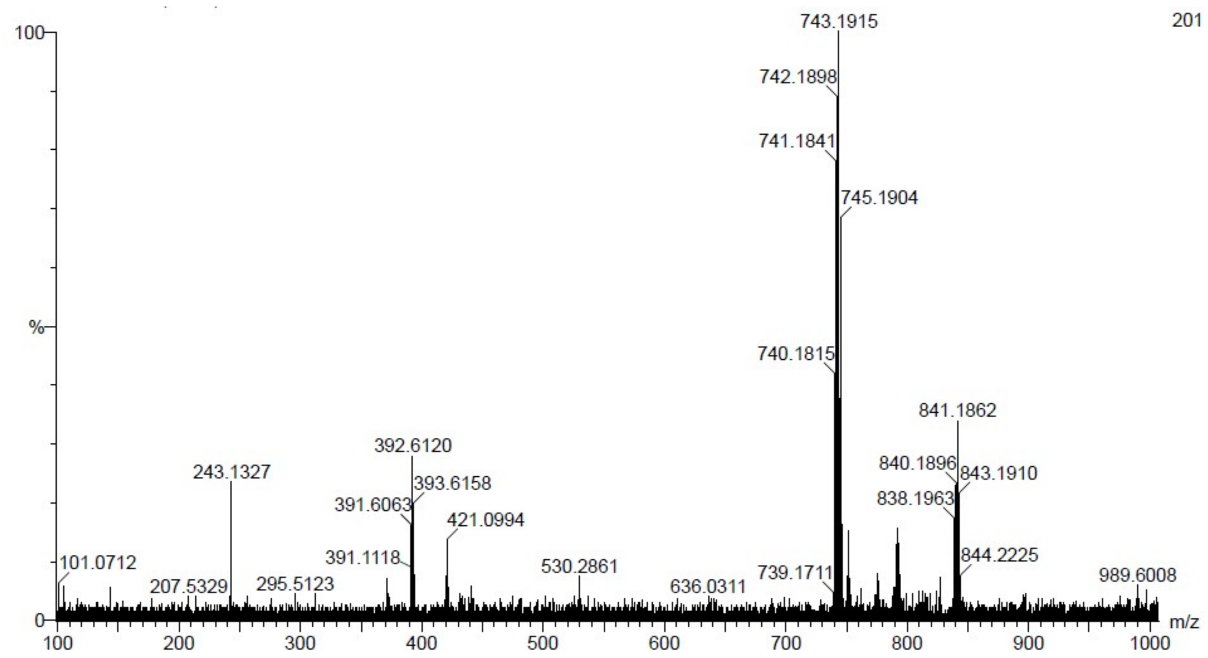

Minimum:				-1.5		
Maximum:		5.0	10.0	100.0		
Mass	Calc. Mass	mDa	PPM	DBE	i-FIT	Formula
743.1915	743.1901	1.4	1.9	14.5	2.2	C26 H35 N9 O7 158Gd

*Figure S55 - ES^+ MS of **7**.*

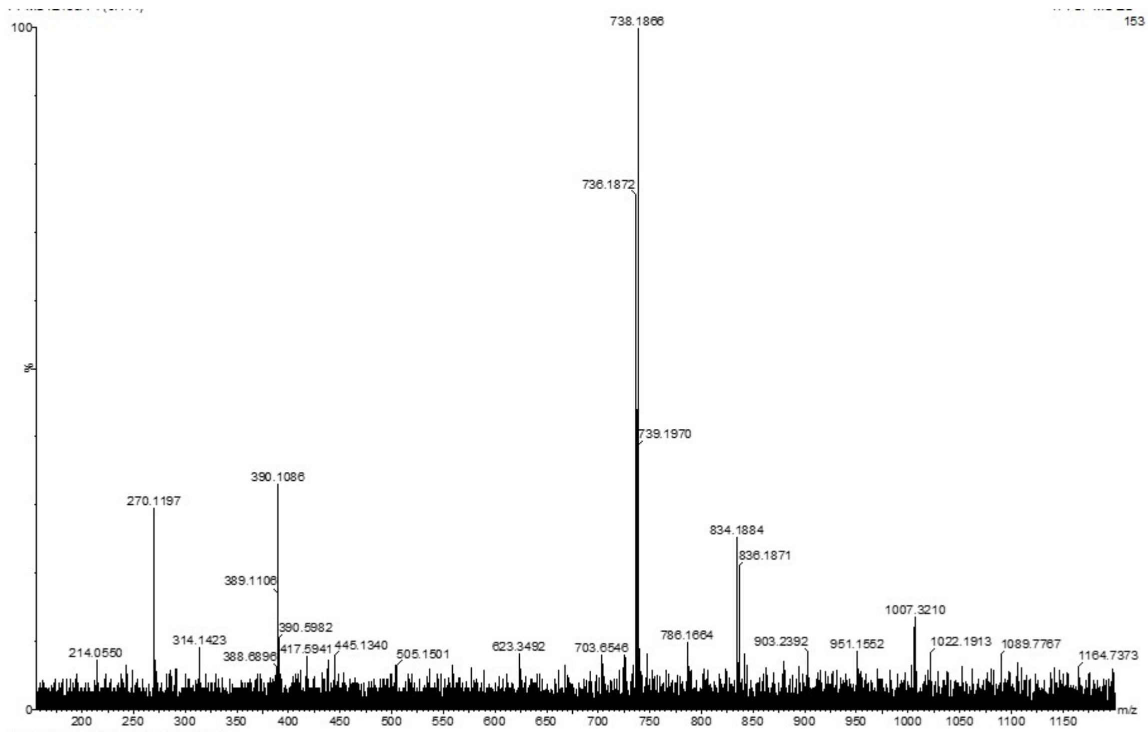

Elemental Composition Report

Mass	Calc. Mass	mDa	PPM	DBE	i-FIT	i-FIT (Norm)	Formula
736.1872	736.1858	1.4	1.9	14.5	74.9	0.0	C26 H35 N9 O7 151Eu

*Figure S56 - ES^+ MS for **8**.*

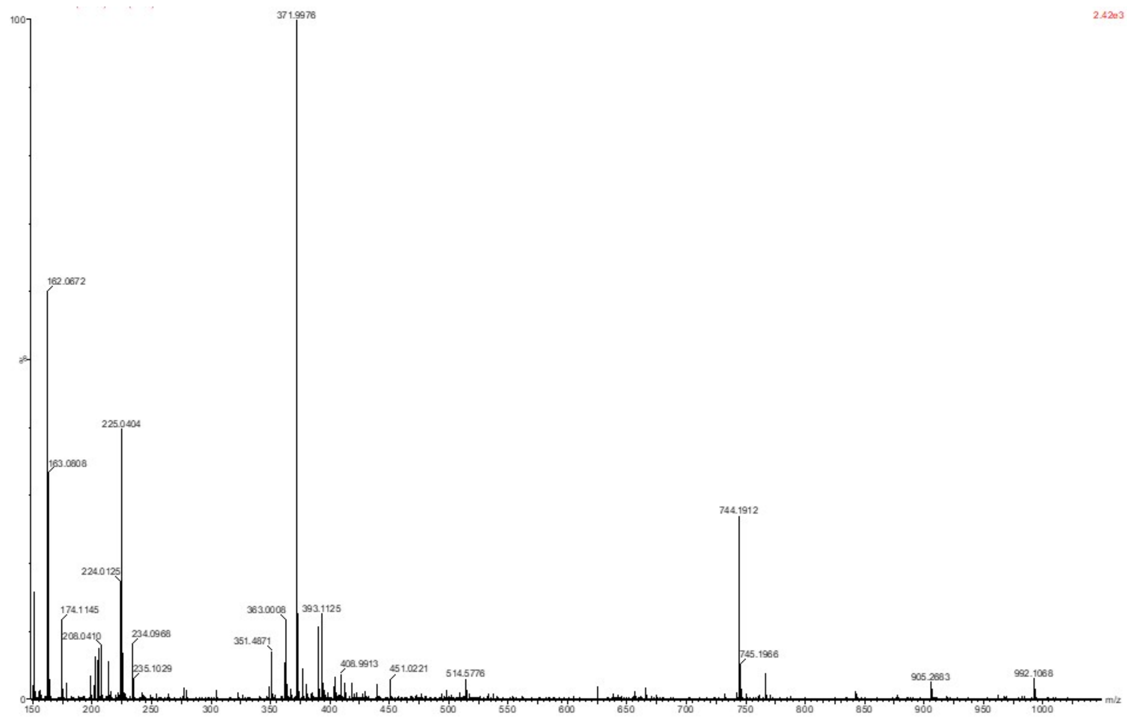

Mass	Calc. Mass	mDa	PPM	DBE	i-FIT	Formula
744.1912	744.1913	-0.1	-0.1	15.0	22.0	C26 H35 N9 O7 159Tb

*Figure S57 - ES$^+$ MS for **9**.*

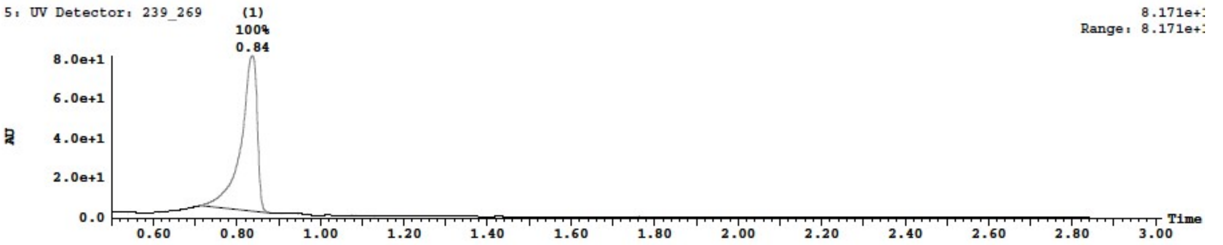

*Figure S58 - LCMS UV-trace for **7**.*

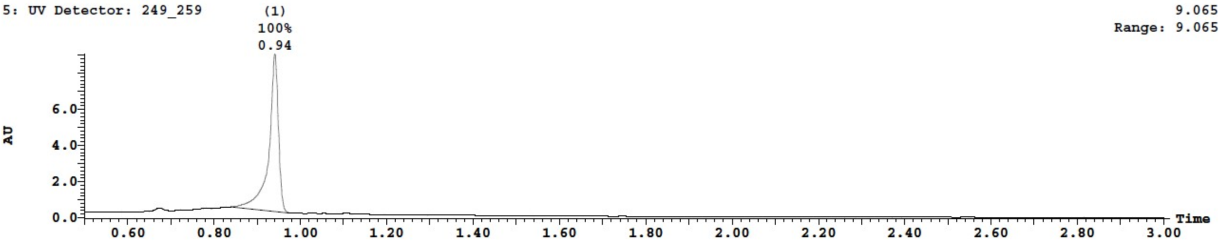

Figure S59 - LCMS UV-trace for 8.

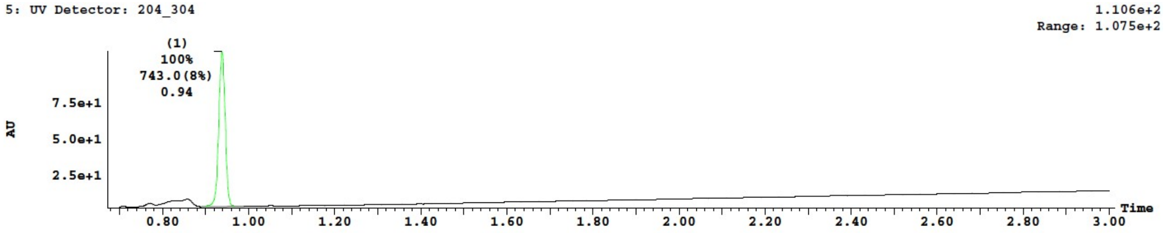

*Figure S60 - LCMS UV-trace for **9**.*

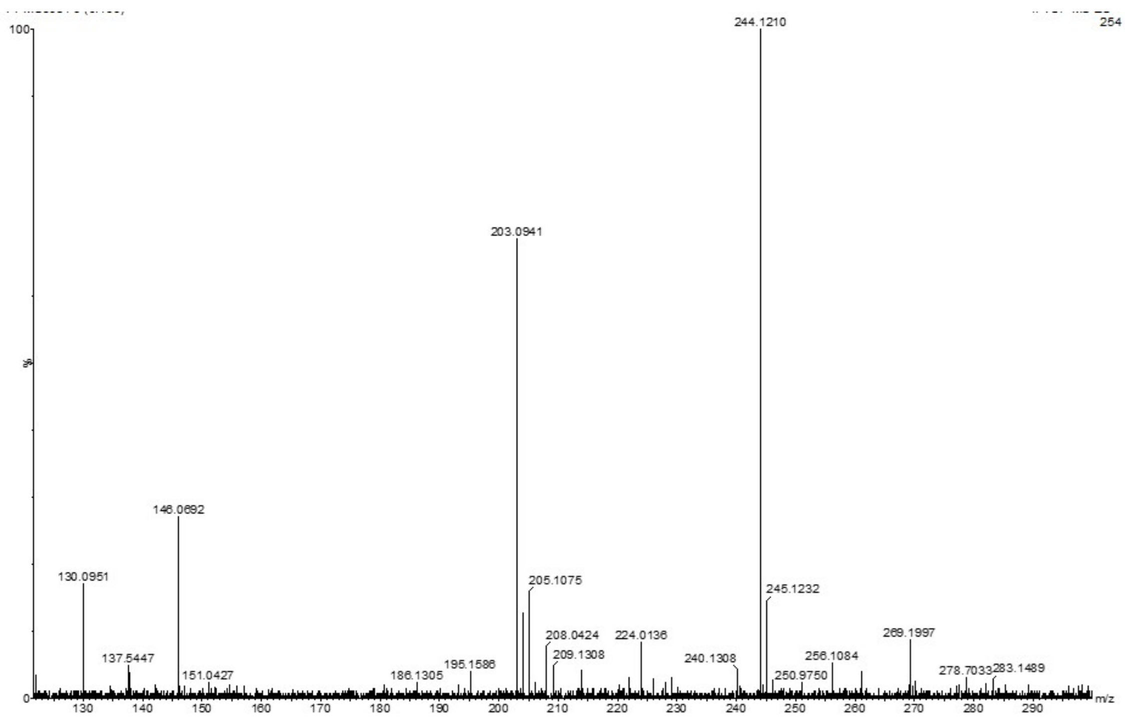

Mass	Calc. Mass	mDa	PPM	DBE	i-FIT	i-FIT (Norm)	Formula
203.0941	203.0933	0.8	3.9	7.5	90.4	0.0	C10 H11 N4 O

*Figure S61 - ES+ MS for **10**.*

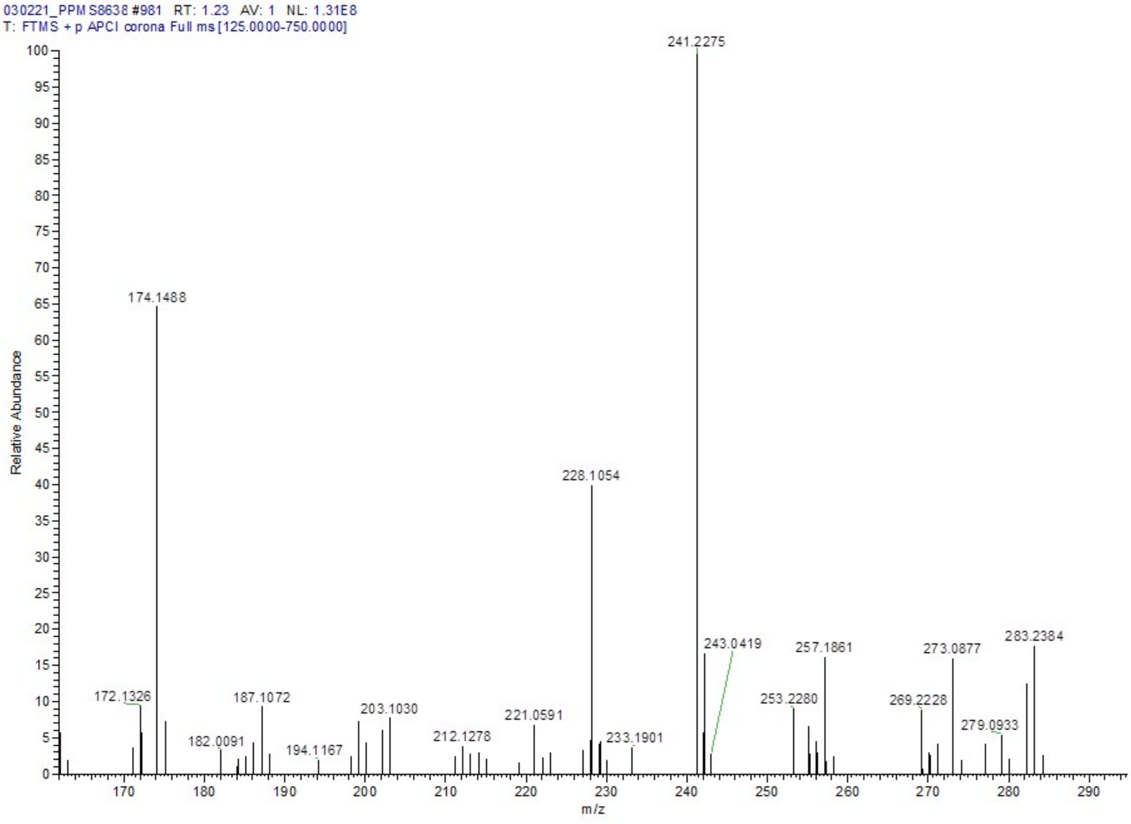

THEORETICAL MASS FOR C10H10N4Cl+ = 221.0589, FOUND MASS = 221.0591

*Figure S62 - APCI MS for **11**.*

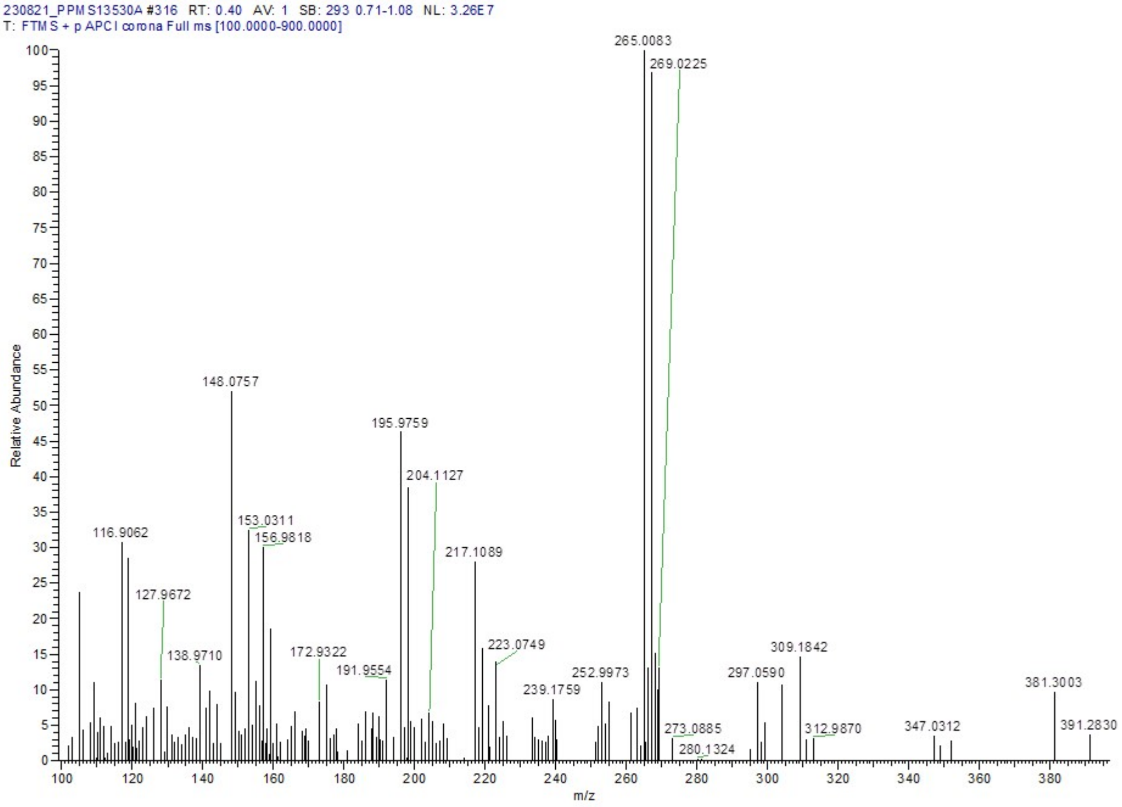

THEORETICAL MASS FOR C10H10N4Br+ = 265.0083. FOUND MASS = 265.0083

*Figure S63 - APCI MS for **12**.*

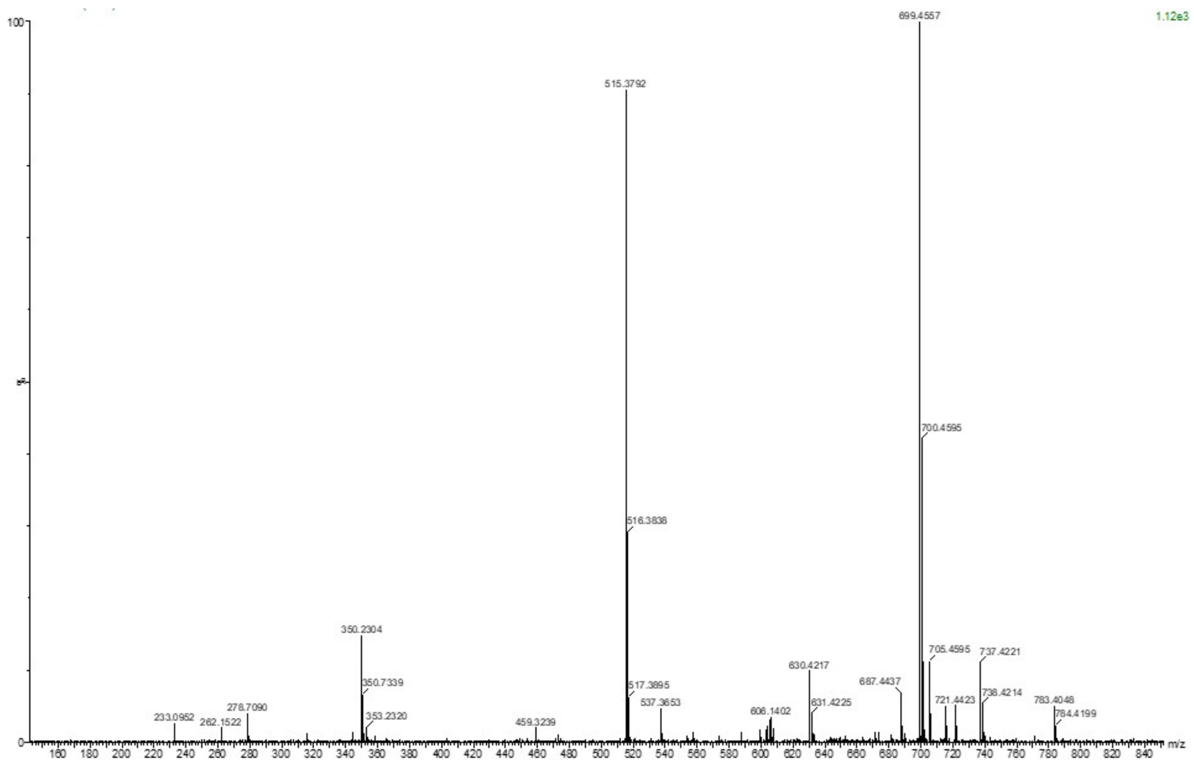

Mass	Calc. Mass	mDa	PPM	DBE	i-FIT	Formula
699.4557	699.4558	-0.1	-0.1	11.5	0.6	C36 H59 N8 O6

*Figure S64 - ES^{+} MS for **13**.*

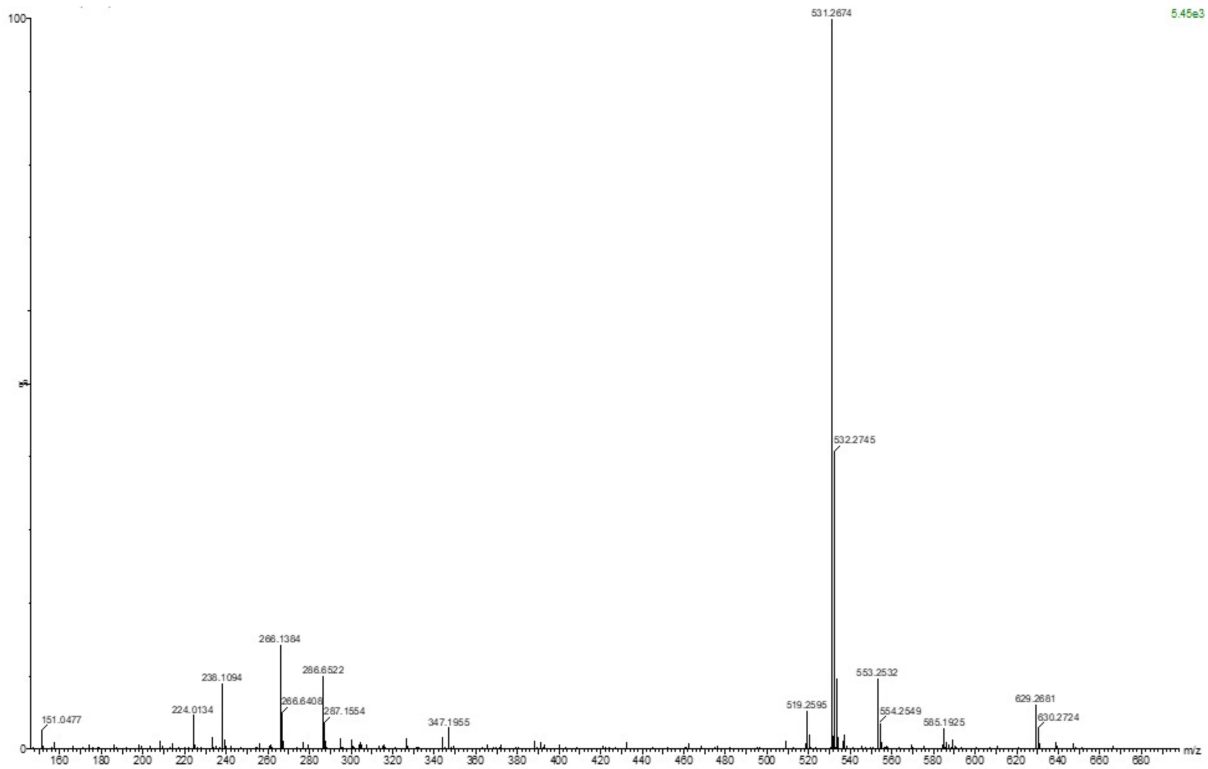

Mass	Calc. Mass	mDa	PPM	DBE	i-FIT	Formula
531.2674	531.2680	-0.6	-1.1	11.5	94.2	C24 H35 N8 O6

*Figure S65 - ES⁺ MS for **14**.*

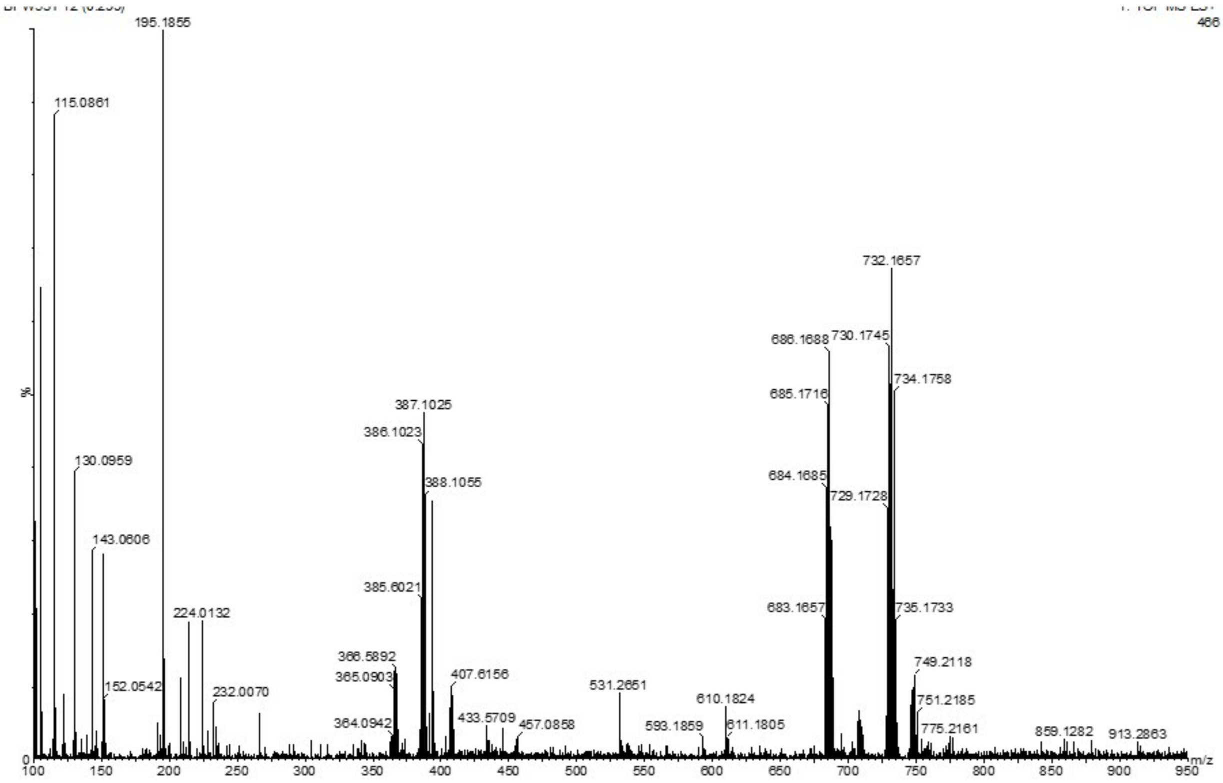

Mass	Calc. Mass	mDa	PPM	DBE	i-FIT	i-FIT (Norm)	Formula
686.1688	686.1686	0.2	0.3	13.5	76.1	0.0	C24 H32 N8 O6 158Gd

*Figure S66 - ES⁺ MS of **15**.*

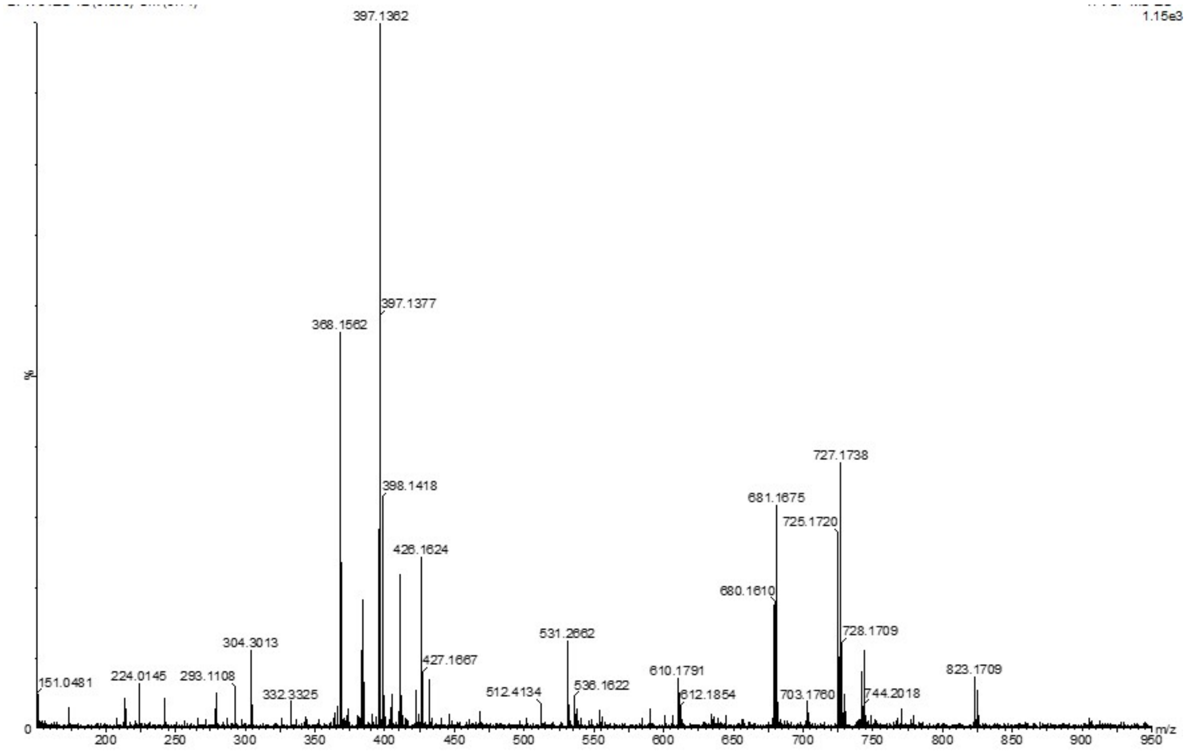

Mass	Calc. Mass	mDa	PPM	DBE	i-FIT	i-FIT (Norm)	Formula
681.1675	681.1657	1.8	2.6	13.5	294.8	0.6	C24 H32 N8 O6 153Eu

*Figure S67 - ES⁺ MS for **16**.*

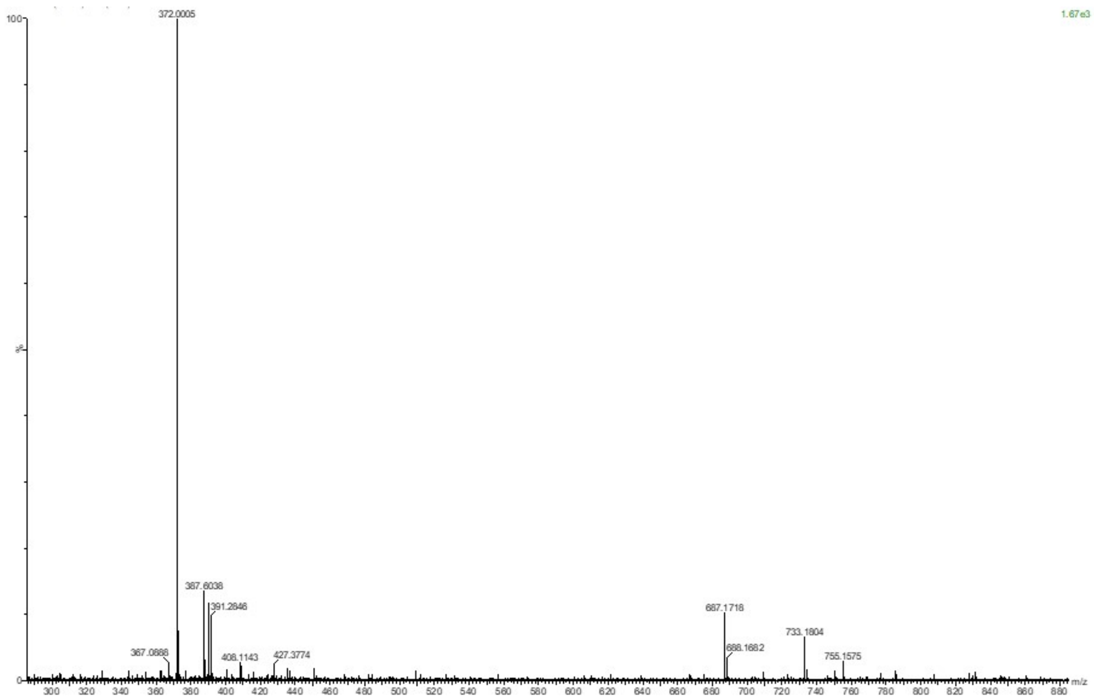

Mass	Calc. Mass	mDa	PPM	DBE	i-FIT	Formula
687.1718	687.1698	2.0	2.9	14.0	2.3	C24 H32 N8 O6 Tb

*Figure S68 - ES+ MS for **17**.*

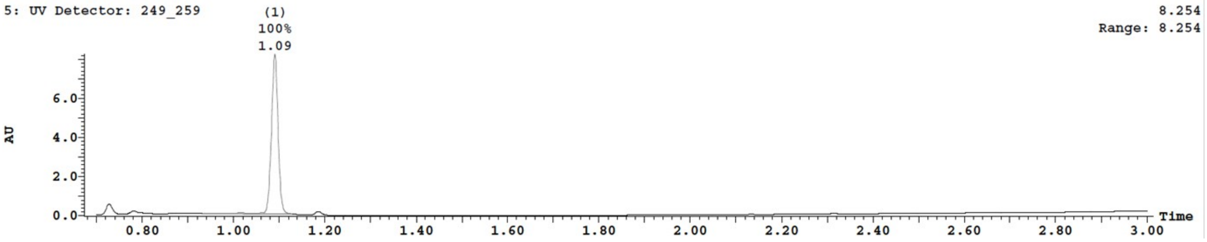

*Figure S69 - LCMS UV-trace for **15**.*

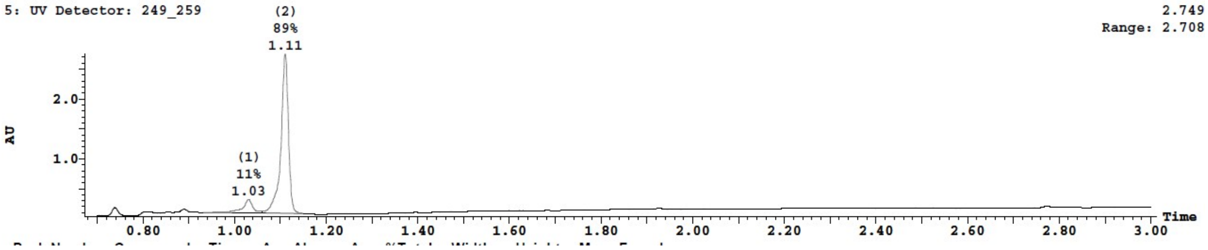

*Figure S70 - LCMS UV-trace for **16**.*

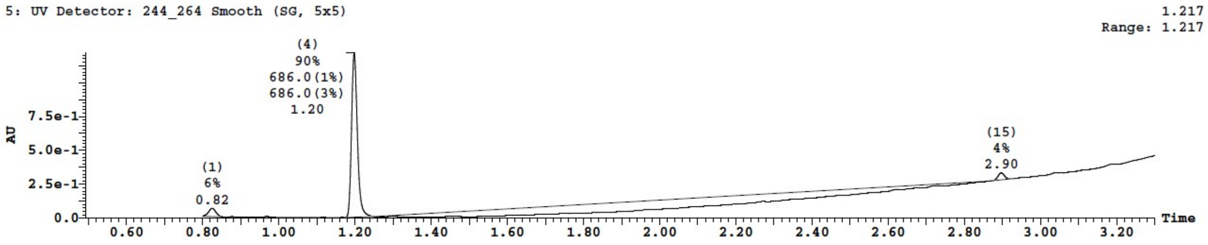

*Figure S71 - LCMS UV-trace for **17**.*

10 - Bio-orthogonal Click Reactions

General Procedure for Click Reactions:

A solution of **7** or **15** (0.2 mM) and **18** (1.0 mM) in 10 mM HEPES (950 µL) and MeCN (50 µL) was stirred at 37 °C for 16 hours. The resulting mixture was analysed *via* analytical HPLC as seen in Figure S71 and S72. A series of isomers of clicked products were isolated from the mixture as Figure S73 or S76, which were further characterised by HRMS (MALDI-TOF) to find the m/z of desired clicked product **19,** or free-ligand clicked products **20** and **21**. The completion of the click reaction was confirmed as m/z of **7** was no longer detected in the isolated mixture, although the retention time of **7** and the clicked products are very close.

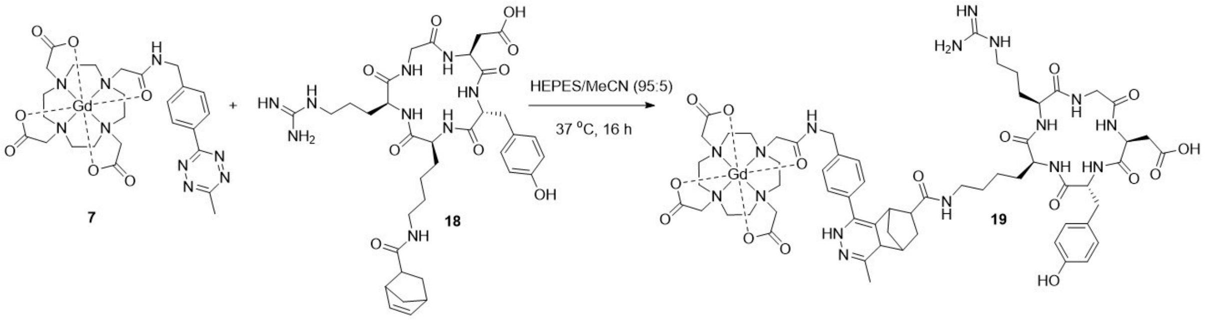

*Scheme S5 - Successful bio-orthogonal click reaction between tetrazine **7** and norbornene **18** to give clicked product **19**.*

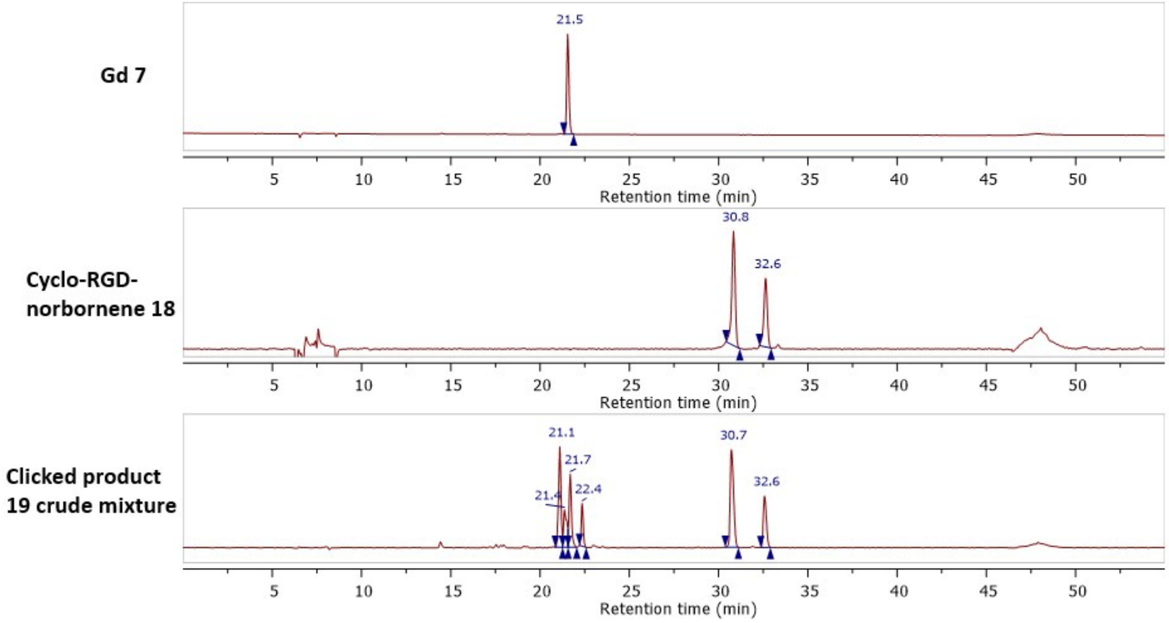

Column: Agilent C18 column (250 mm × 4.6 mm), particle size 5 µm.
Mobile phases: A, H_2O + 0.1 % TFA; B: MeCN + 0.1 % TFA. Flow rate: 0.5 mL/min.
Gradient: 0 min, 5 %; 40 min, 50 %; 41 min, 100%
All three chromatograms based on absorption at 280 nm

*Figure S72 - HPLC traces for **7**, **18**, and the crude clicked product **19** mixture.*

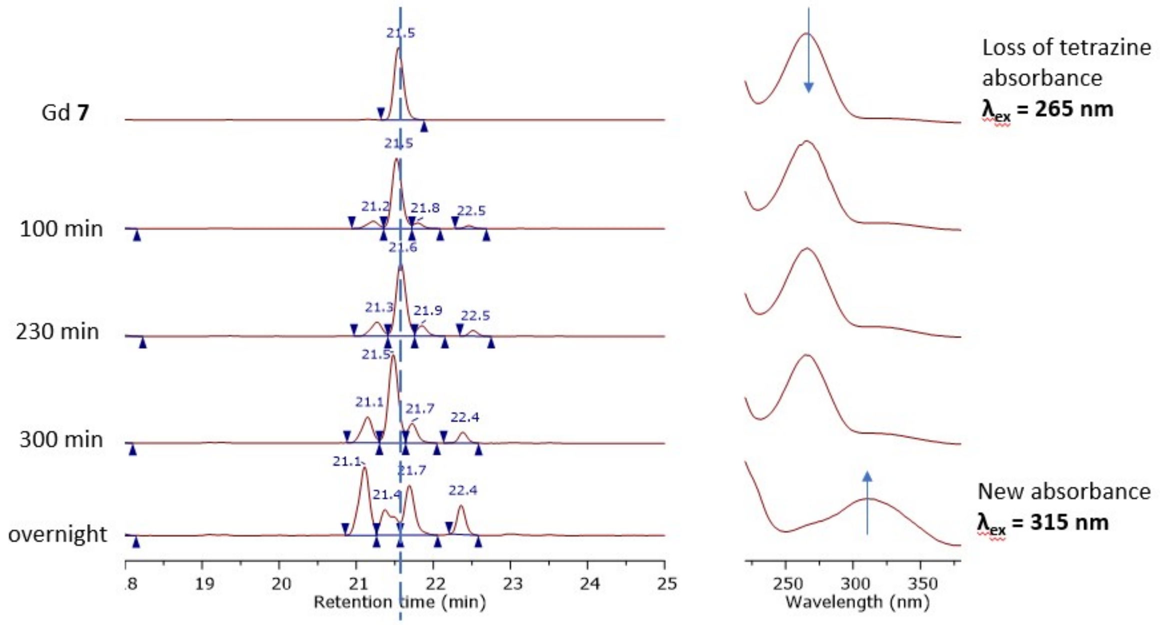

*Figure S73 - HPLC traces showing the crude clicked mixture **19** over time along with the absorbance spectra.*

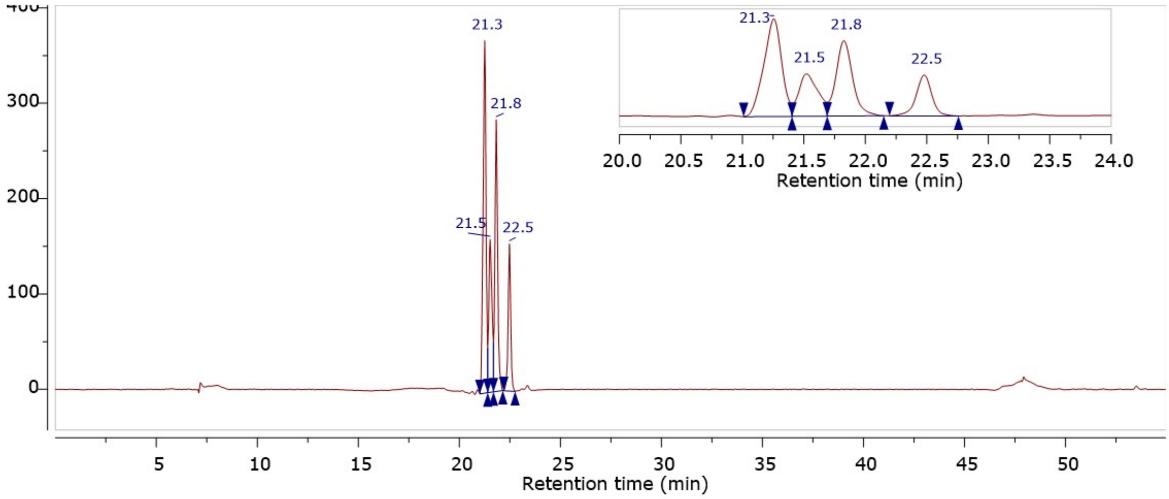

*Figure S74 - HPLC trace for the purified clicked product **19** showing 4 major diastereoisomers.*

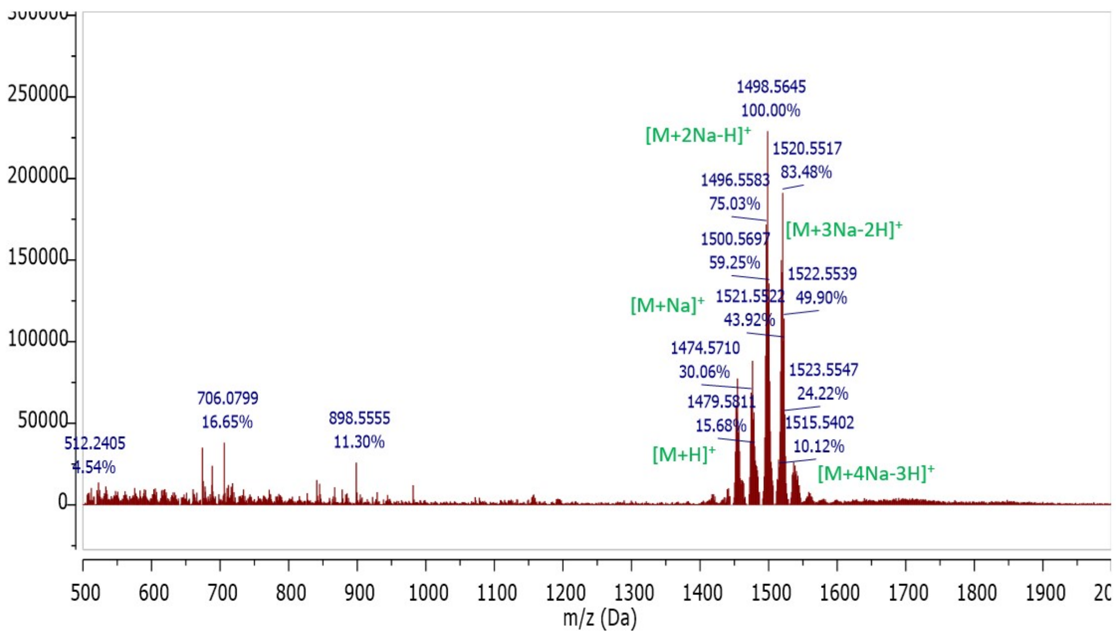

*Figure S75 - MALDI MS for the purified clicked product **19**.*

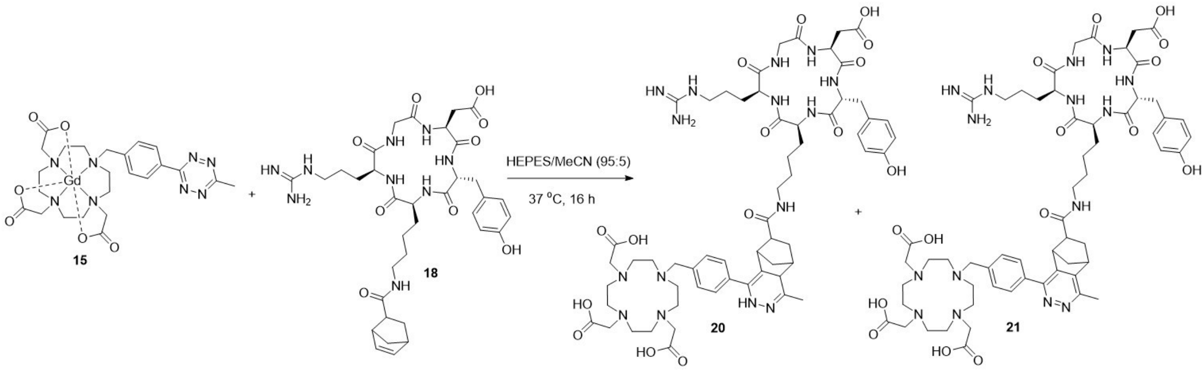

*Scheme S6 - Attempted click reaction between tetrazine **15** and norbornene **18** giving free-ligand clicked products **20** and **21**.*

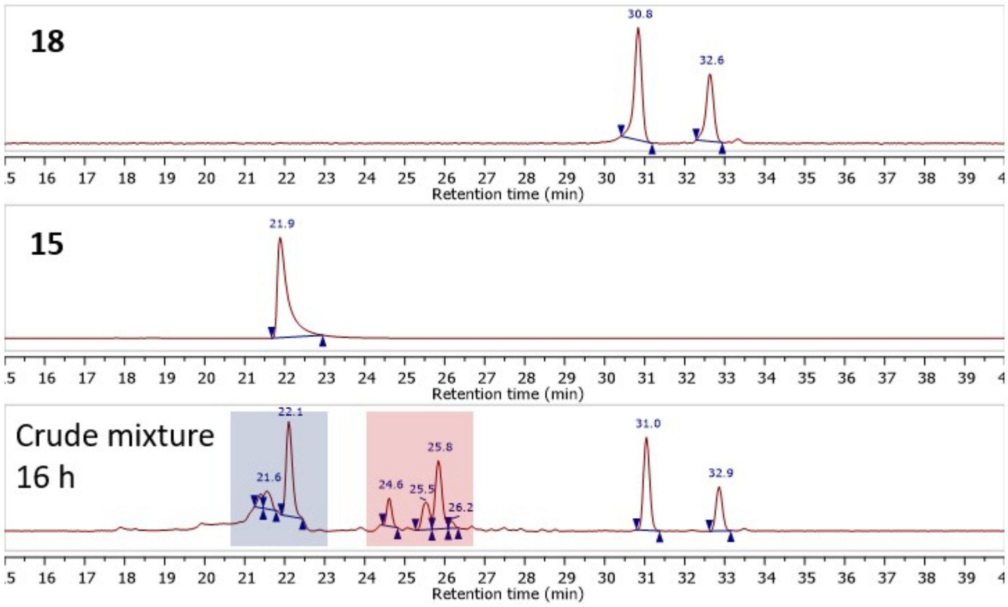

*Figure S76 - Stacked HPLC traces for **18, 15**, and the crude clicked mixture showing 2 major products **20** and **21**.*

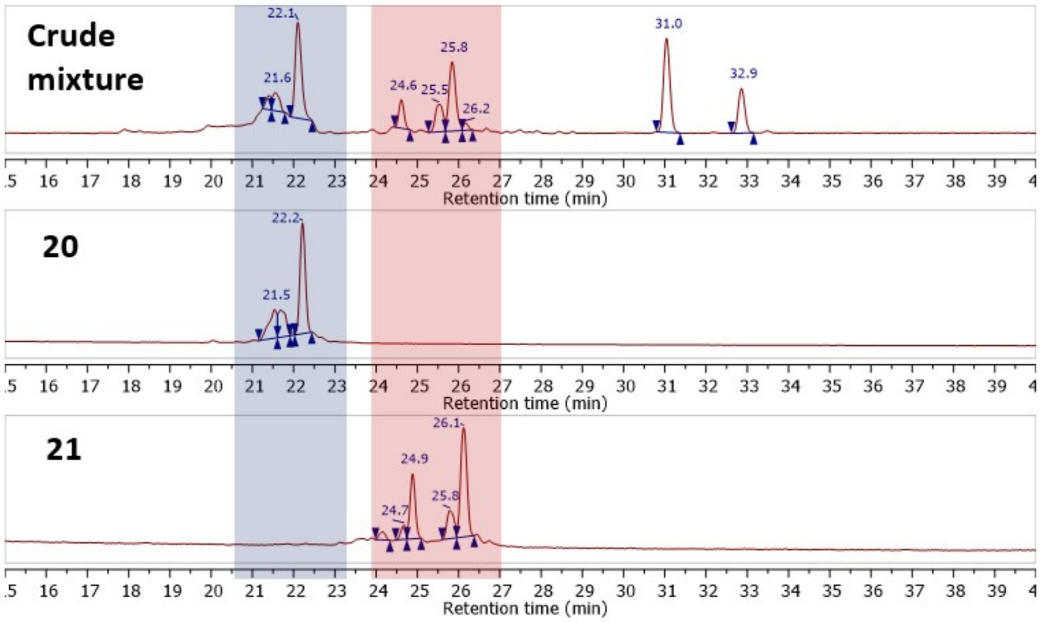

*Figure S77 - HPLC traces showing the crude click mixture and isolated pure samples of **20** and **21.***

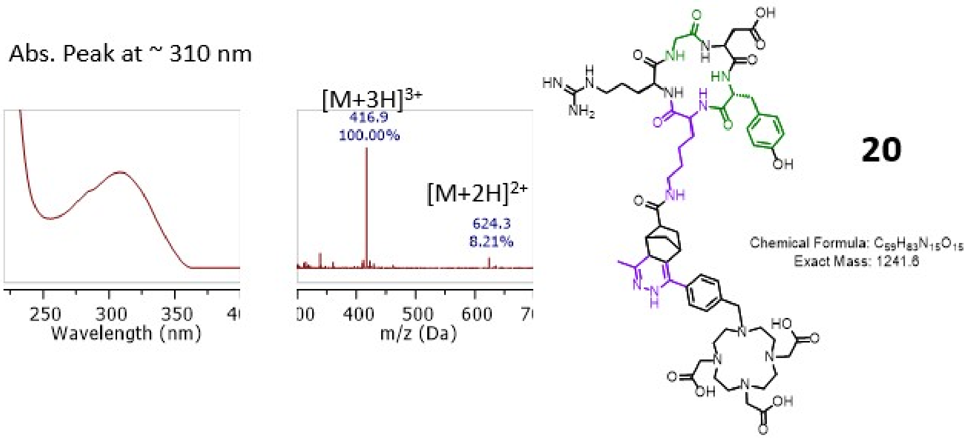

*Figure S78 - UV-vis absorbance spectrum and MALDI MS for **20**.*

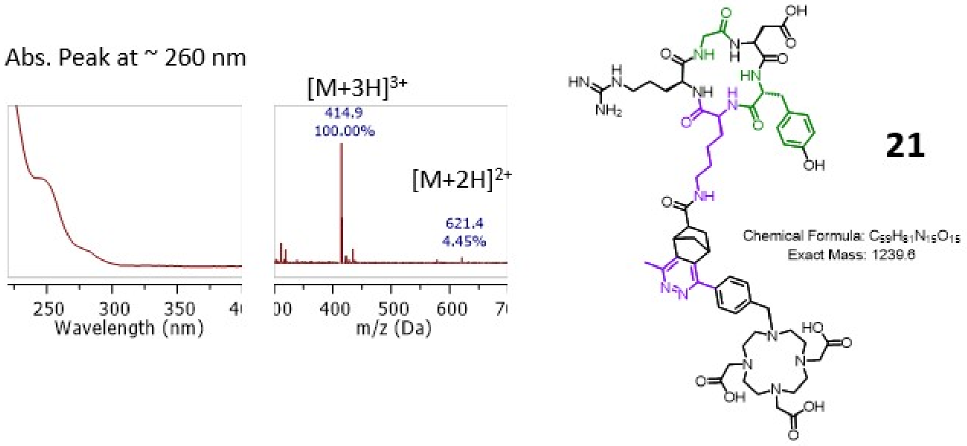

*Figure S79 – UV-vis absorbance spectrum and MALDI MS for **21***

11 - References

1 D. M. Corsi, C. Platas-Iglesias, H. Van Bekkum and J. A. Peters, *Magn. Reson. Chem.*, 2001, **39**, 723–726.

2 N. G. Chabloz, H. L. Perry, I. C. Yoon, A. J. Coulson, A. J. P. White, G. J. Stasiuk, R. M. Botnar and J. D. E. T. Wilton-Ely, *Chem. Eur. J.*, 2020, **26**, 4552–4566.

3 M. Rohrer, H. Bauer, J. Mintorovitch, M. Requardt and H. J. Weinmann, *Invest. Radiol.*, 2005, **40**, 715–724.

4 B. Jagadish, G. L. Brickert-Albrecht, G. S. Nichol, E. A. Mash and N. Raghunand, *Tetrahedron Lett.*, 2011, **52**, 2058–2061.

5 J. Hernández-Gil, M. Braga, B. I. Harriss, L. S. Carroll, C. H. Leow, M. X. Tang, E. O. Aboagye and N. J. Long, *Chem. Sci.*, 2019, **10**, 5603–5615.

6 J. Yang, M. R. Karver, W. Li, S. Sahu and N. K. Devaraj, *Angew. Chem. Int. Ed.*, 2012, **51**, 5222–5225.

7 H. L. Evans, L. Carroll, E. O. Aboagye and A. C. Spivey, *J. Label. Compd. Radiopharm.*, 2014, **57**, 291–297.

8 A. Beeby, I. M. Clarkson, R. S. Dickins, S. Faulkner, D. Parker, L. Royle, A. S. De Sousa, J. A. G.

Williams and M. Woods, *J. Chem. Soc. Perkin Trans. 2*, 1999, **2**, 493–503.
